# Photosynthesis across African cassava germplasm is limited by Rubisco and mesophyll conductance at steady state, but by stomatal conductance in fluctuating light

**DOI:** 10.1111/nph.16142

**Published:** 2019-08-25

**Authors:** Amanda P. De Souza, Yu Wang, Douglas J. Orr, Elizabete Carmo-Silva, Stephen P. Long

**Affiliations:** 1 Carl R Woese Institute for Genomic Biology, University of Illinois at Urbana-Champaign, Urbana, IL 61801, USA; 2 Lancaster Environment Centre, Lancaster University, Lancaster, LA1 4YQ, UK

**Keywords:** cassava breeding, crop yield, food security, genetic engineering, *Manihot esculenta*, photosynthesis, Rubisco activase, sub-Saharan Africa

## Abstract

Summary

- Sub-Saharan Africa is projected to see a 55% increase in food demand by 2035, where cassava (*Manihot esculenta*) is the most widely planted crop and a major calorie source. Yet, cassava yield in this region has not increased significantly for 13 yr. Improvement of genetic yield potential, the basis of the first Green Revolution, could be realized by improving photosynthetic efficiency. First, the factors limiting photosynthesis and their genetic variability within extant germplasm must be understood.
- Biochemical and diffusive limitations to leaf photosynthetic CO_2_ uptake under steady state and fluctuating light in 13 farm-preferred and high-yielding African cultivars were analyzed. A cassava leaf metabolic model was developed to quantify the value of overcoming limitations to leaf photosynthesis.
- At steady state, *in vivo* Rubisco activity and mesophyll conductance accounted for 84% of the limitation. Under nonsteady-state conditions of shade to sun transition, stomatal conductance was the major limitation, resulting in an estimated 13% and 5% losses in CO_2_ uptake and water use efficiency, across a diurnal period. Triose phosphate utilization, although sufficient to support observed rates, would limit improvement in leaf photosynthesis to 33%, unless improved itself.
- The variation of carbon assimilation among cultivars was three times greater under non-steady state compared to steady state, pinpointing important overlooked breeding targets for improved photosynthetic efficiency in cassava.

## Introduction

Rising global population coupled with increased urbanization is predicted to increase food demand by 60% by 2050. Demand increase will be greatest in Sub-Saharan Africa where the population is expected to double by 2050 (van Ittersum *et al*., 2016; United Nations, 2017). In this region, where cassava (*Manihot esculenta* Crantz) is the most widely planted crop (FAOSTAT, 2019a), food demand is projected to rise by 55% within just 15 yr (World Bank, 2017). For a variety of cultural and pragmatic reasons, cassava is also the preferred staple food source for many smallholder farmers who constitute the bulk of the population. Dependence on cassava in Africa is underlined by the fact that it accounts for a higher proportion of food consumption per person than any staple in any part of the world (i.e. 0.4 kg per person d^−1^) (Henry *et al*., 2004). This makes cassava virtually irreplaceable in the fight against hunger in this key and most vulnerable region of the world (Nassar & Ortiz, 2010). Its importance as a cash crop has also increased with more widespread usage by industry (Kleih *et al*., 2013; Uchechykwu-Agua *et al*., 2015). For smallholder farmers, increased yields mean that when family needs are exceeded, the surpluses can be sold to provide other household needs. However, cassava yield in Sub-Saharan Africa has not increased over the last 13 yr (De Souza *et al*., 2017; FAOSTAT, 2019b). Moreover, the genetic progress achieved in breeding programs for increased yield has slowed significantly in recent years (Ceballos *et al*., 2016). In Africa, the focus of research and breeding programs has necessarily been on disease and pest resistance, as these are major threats to yield increase (Alene *et al*., 2018). Improved drought-tolerant plants can also enhance its productivity in African soils, despite the fact that cassava already has a relatively high yield under drought conditions (Okogbenin *et al*., 2013). However, increasing yield also depends on increasing genetic yield potential, that is the yield that can be achieved in the absence of pests, disease, water and nutrient limitations. While this might seem of limited value for a crop like cassava, which is often nutrient-, water- or disease-limited, experience with other crops has shown that raising the genetic yield potential not only increases the maximum yields achieved in a region but also increases the minimum yields, that is those achieved under limiting conditions (Koester *et al*., 2014).

Increased yield potential can be achieved by improving photo-synthetic efficiency (Long *et al*., 2006). Comparing the photosynthetic rates between landraces and improved lines, there is no evidence that photosynthesis in cassava has been improved through breeding (De Souza *et al*., 2017; De Souza & Long, 2018). Indeed, the conversion efficiency in cassava, which reflects its photosynthetic rates, is just one-seventh of the theoretical value for C_3_ plants (De Souza *et al*., 2017). The validation that increased photosynthetic efficiency can improve yield potential in cassava has been shown by Free Air CO_2_ Enrichment (FACE) experiments. Under open-air field CO_2_ concentration elevation, leaf photosynthesis was increased by 30%, resulting in a doubling in cassava yield (Rosenthal *et al*., 2012). This shows that, if photosynthetic efficiency can be genetically improved in cassava, yield potential will also be substantially increased.

Genetic improvements depend on an understanding of the pre-existing diversity for a particular desired trait within an available germplasm. For bioengineering strategies, it is also key to understand the limitations of the desirable trait to design suitable approaches to overcome identified limitations. In cassava, it is remarkable that the genetic variability in photosynthesis is barely known and limitations have not been analyzed (Ceballos *et al*., 2004). Although the diversity in steady-state photosynthesis of South American cassava cultivars has been evaluated (El-Sharkawy, 2006, 2016), very little is known about African germplasm (De Souza *et al*., 2017; De Souza & Long, 2018).

Under steady-state conditions, *in vivo* biochemical and diffusive limitations to leaf photosynthesis may be deduced from the response of net leaf CO_2_ uptake under saturating light (*A*_sat_) to intracellular CO_2_ concentrations (*c*_i_)(Long & Bernacchi, 2003).These limitations are the apparent maximum *in vivo* Rubisco activity (*V*_max_), maximum electron transport rate (*J*_max_) and the maximum rate of triose phosphate utilization (*V*_TPU_). Mesophyll conductance to CO_2_ diffusion (*g*_m_) is obtained by combining the *A/c*_i_ curves with modulated Chl fluorescence (Harley *et al*., 1992; Long & Bernacchi, 2003). In a previous study, steady-state photosynthesis in four African cassava cultivars was found to be limited by *V*_cmax_, which suggested that Rubisco activity and/or *g*_m_ were restricting CO_2_ uptake (De Souza & Long, 2018). While these results provided an indication that there was genotypic variation, they did not account for the full range of quantitative limitations of photosynthesis and indicated the need for evaluation of a larger number of farmer-preferred cultivars to provide a more realistic assessment of the photo-synthetic limitations under steady-state conditions.

Improvement of photosynthetic efficiency has focused almost entirely on steady-state and light-saturating conditions. However, in field crop canopies including that of cassava, lighting is almost never at steady-state due to continuous fluctuations in light (Pearcy, 1990). Although cassava is grown in tropical and subtropical environments where the intensity of sunlight is high, the amount of direct light received by a leaf reduces progressively with the depth into the canopy. A leaf in the shade of another receives about 1/10^th^ of the light of one in full sun (Zhu *et al*., 2004). Leaf area indices of cassava crops in Sub-Saharan Africa may average little more than 2 (Biratu *et al*., 2018), so does shading matter? Zhu *et al*. (2004) show, assuming a random distribution of leaves, that even on a clear sky day, a second layer of leaves will experience over 20 shade–sun transitions during the course of a day, simply due to intermittent shading by other leaves as the sun crosses the sky over the course of a day. Furthermore, cassava in Sub-Saharan Africa is often intercropped with grains that grow faster and mature earlier (Mutsaers *et al*., 1993), imposing more frequent shading. Additionally, in this region intermittent cloud cover is common during the wet growing seasons (Bourassa *et al*., 2005), promoting further incidence of shade–sun transitions. While there is limited information on steady-state photosynthesis and its limitations in cassava, to our knowledge there is none on photosynthetic limitations under fluctuating light conditions. Critically, when a leaf transitions from shade to full sunlight, there is a delay of minutes in achieving its maximum photosynthetic rates. This delay can be caused either by the rate of activation of Rubisco (Mott & Woodrow, 2000; Soleh *et al*., 2016), the rate of stomatal opening or both (Allen & Pearcy, 2000; McAusland *et al*., 2016). Depending on how slow this transition is, it adversely affects daily photosynthetic carbon gain resulting in lower biomass production. In wheat, for instance, the slow photosynthetic adjustment from shade to sun was calculated to result in a 21% loss of net canopy CO_2_ assimilation and productivity (Taylor & Long, 2017). Considering the converse situation, when a leaf transitions from light to shade, photosynthesis declines immediately while stomatal responses are much slower, lowering by *c*. 20% the intrinsic efficiency of water use (Lawson & Blatt, 2014). On such transitions, it also takes many minutes for photosynthesis to acclimate to the lower light conditions, and over the course of a growing season this can cost 20–40% of potential productivity (Zhu *et al*., 2004; Kromdijk *et al*., 2016). In cassava, there is no information on how photosynthesis and stomatal conductance respond to fluctuations in light, nor what limits the speed of adjustment and, in turn, efficiency. This information would be crucial for developing strategies to improve carbon gain and water-use efficiency in this crop.

In addition to the physiological measurements, mechanistic models of photosynthetic metabolism provide a means to test hypotheses related to different *in vivo* dynamic behaviors, and provide a broader guide to assess quantitatively the value of varying individual traits affecting photosynthetic efficiency (Zhu *et al*., 2007, 2013). Previous model predictions have determined potential routes for improvements in photosynthesis (Zhu *et al*., 2004; Long *et al*., 2006) that were later successfully translated to yield increases (Lefebvre *et al*., 2005; Kromdijk *et al*., 2016; South *et al*., 2019). This approach is used here, integrating physiological and biochemical measurements to then predict modifications that could improve photosynthetic efficiency, and by how much.

Here we quantified limitations to photosynthesis in 13 African farm-preferred and high-yielding cassava cultivars under steady-state and fluctuating light conditions, aiming to determine the potential for improving cassava photosynthetic efficiency. A metabolic model of photosynthesis in cassava was developed using the measurements to explore the underlying traits that could give the largest improvements in photosynthetic and water-use efficiencies, with a focus on nonsteady-state conditions.

## Materials and Methods

### Plant material and growth conditions

Thirteen farm-preferred cassava (*Manihot esculenta* Crantz) cultivars from Africa were chosen for this study, including five landraces (MBundumali, TME3, TME419, TME7 and TME693) and eight improved lines (TMS01/1412, TMS30001, TMS30572, TMS96/1632, TMS97/2205, TMS98/0002, TMS98/0505 and TMS98/0581). Measurements were taken in two independent experiments (from 23 May to 1 July 2017 and from 1 May to 15 June 2018) in a controlled environmental glasshouse at the University of Illinois at Urbana-Champaign; cultivars TMS97/2205 and TMS98/0505 were only evaluated in 2017. For both experiments, all cultivars were propagated *in vitro* and transferred to the glasshouse as previously described by De Souza & Long (2018). Plants were grown in 14-liter pots, which allowed a plant biomass : pot size ratio of 1 g (DM) dm^−3^, which is suggested to avoid any pot size limitation to growth (Poorter *et al*., 2011). Air temperature in the glasshouse was 28 ± 4°C, water vapor pressure deficit (VPD) was 1.5 ± 0.6 kPa and the average light intensity was 1200 ± 500 μmol m^−2^ s^−1^. In each experiment, three to four biological replicates (individual plants) of each cultivar were measured in a completely randomized experimental design. Pots were distributed with 25 cm spacing and their positions in the glasshouse were re-randomized every 4–5 d to circumvent confounding cultivar with any environmental variation within the glasshouse. Plants were watered to pot capacity every 2–3 d allowing the soil surface to dry between the watering. Measurements were taken on plants 40 d after transplantation. At that stage, plants had, on average, 16.3 g of total biomass (Supporting Information Fig. S1).

### Gas exchange and assessment of photosynthetic limitations under steady state

Leaf CO_2_ assimilation and transpiration of the central foliole of the youngest fully expanded leaf was measured on 40 d old plants with a portable gas exchange system integrated with a leaf cuvette including a modulated Chl fluorometer and light source (LI-6400XT and Li-6400-40; Li-Cor, Lincoln, NE, USA). To define the response of leaf net CO_2_ uptake to intracellular CO_2_ concentration (*A/c*_i_ curves), the leaf was acclimated to a saturating light intensity of 1500 μmol m^−2^ s^−1^ (*c.* 90% red and 10% blue light) and a CO_2_ concentration of 400 μmol mol^−1^ inside the cuvette. After steady-states for both *A* and stomatal conductance (*g*_s_) were obtained, the chamber inlet [CO_2_] was varied according to the following sequence: 400, 270, 150, 100, 75, 50, 400, 400, 600, 800, 1100, 1300 and 1500 μmol mol^−1^. The gas exchange measurements were recorded simultaneously with modulated Chl fluorescence as a 10 s average after the conditions inside the cuvette were stable at each [CO_2_]. The block temperature was set to 28°C, VPD inside the cuvette was maintained at 1.5 ± 0.3 kPa and the air flow at 300 μmol s^−1^.

The apparent maxima of Rubisco carboxylation rate (*V*_cmax_), regeneration of ribulose-1,5-biphosphate expressed as electron transport rate (*J*_max_) and triose phosphate utilization (*V*_TPU_) were calculated from the *A/c*_i_ curves using the equations from von Caemmerer (2000). Before fitting the curves, values for each individual curve were corrected for diffusive leaks between the cuvette and external environment (Bernacchi *et al*., 2001). Calculated values were adjusted to 25°C, following the equations for temperature response as described by Bernacchi *et al*. (2001, 2003) and McMurtrie & Wang (1993). Stomatal conductance and operating *c*_i_ were obtained from the data points collected at 400 μmol mol^−1^ [CO_2_]. The intrinsic water use efficiency (*i*WUE) was calculated by dividing *A* by *g*_s_ at this same CO_2_ concentration.

Mesophyll conductance (*g*_s_) and [CO_2_] inside the chloroplast (*c*_s_) were calculated for ambient [CO_2_] (*c*. 400 μmol mol^−1^) according to the variable *J* method (Harley *et al*., 1992). The CO_2_ compensation point (Γ*) and respiration (*R*_d_) values necessary for *g*_m_ calculation were estimated for each replicate according to Moualeu-Ngangue *et al*. (2017). *V*_cmax_ and *J*_max_, based on chloroplast [CO_2_] derived from measured *g*_m_, were obtained by using a nonlinear analysis with the Marquart method (Moualeu-Ngangue *et al*., 2017).

To determine photosynthetic limitations under steady-state, the stomatal, mesophyll and biochemical relative limitations were calculated following Grassi & Magnani (2005). Values for Rubisco Michaelis constants for CO_2_ (*K*_c_) and for O_2_ (*K*_o_) in these calculations were from Bernacchi *et al*. (2001).

### Gas exchange and quantification of diffusional and biochemical limitations under fluctuating light conditions

To evaluate the response of gas exchange in cassava under fluctuating light, two measurements were performed: photosynthetic response to the transition from deep shade to high light (i.e. induction curves), and photosynthetic response to the transition from high to low and back to high light (i.e. relaxation curves followed by induction curves). The measurements were performed on 35–40 d old plants using the same equipment described above for the steady-state measurements.

For the induction curves, plants were maintained in the dark overnight. Before the measurements, the central foliole of the youngest fully expanded leaf was acclimated to the conditions of the LI-6400 cuvette for 20 min, still in the dark. CO_2_ concentration inside the cuvette was 400 μmol mol^−1^, air temperature 28 ± 2°C and VPD 1.5 ± 0.3 kPa. After 20 min, leaves were pre-illuminated with 50 μmol m^−2^ s^−1^ (deep shade) of photosynthetic photon flux density (PPFD) for 5 min to induce photosynthesis. Then, the light was increased to PPFD of 1500 μmol m^−2^ s^−1^ for 30 min, simulating a shade–sun transition. Gas exchange parameters were recorded every 10 s. For each induction curve, the time to reach 50% of maximum photosynthesis (*T*_50A_), the time to reach 90% of maximum photosynthesis (*T*_90A_), the cumulative CO_2_ fixation in the first 5 min after photosynthetic induction (CCF) and the time to reach 50% of maximum stomatal conductance (*T*_50gs_) were calculated. Maximum light-saturated leaf CO_2_ uptake and maximum stomatal conductance in the induction curves were considered to be that obtained after 30 min under high light. Stomatal conductance at the beginning of induction (*g*_sT0_) was the last value obtained before increasing the light to 1500 μmol m^−2^ s^−1^ PPFD. To investigate the impact of the rate at which the stomata opened on the induction of photosynthesis, a similar induction curve was performed, using a low CO_2_ concentration of 100 ppm inside the chamber during the deep shade period to maintain stomatal opening (Taylor & Long, 2017).

The variation in induction rates of three cultivars with contrasting responses were further evaluated with induction curves at five CO_2_ concentrations (75, 150, 270, 400 and 600 μmol mol^−1^ CO_2_). From these curves, usually referred to as dynamic *A/c*_i_ curves (Soleh *et al*., 2016; Taylor & Long, 2017; Salter *et al*., 2019), *V*_cmax_ and stomatal limitation under nonsteady-state conditions were calculated using the equations described by Soleh *et al*.(2016).

Acclimation of photosynthesis to shade, on a sun-shade transition, was characterized after a steady-state rate of leaf CO_2_ uptake was obtained at 1500 μmol m^−2^ s^−1^ PPFD (*c*. 40 min). Once in steady-state, the light was decreased to 10% of the initial value (i.e. 150 μmolm^−2^ s^−1^ PPFD), and plants were kept under this light intensity for 40 min. Then, the light was increased to 1500 μmol m^−^ s^−1^ PPFD again, for an additional 40 min. Gas exchange was recorded every 10 s. Rate constants were calculated for the increase in *g*_s_ on transfer to 1500 μmol m^−2^ s^−1^ PPFD (*k*_i_), and again for the decrease in *g*_s_ on return to 150 μmol m^−2^ s^−1^ PPFD (*k*_d_). Measured time series for stomatal conductance changes were fit to the following equation (Vialet-Chabrand *et al*., 2017):

$$g_{\text{s}}=\left(g_{\text{max}}-g_{0}\right)e^{-kt}+g_{0}$$

where *g*_max_ is the maximum stomata conductance, *g*_0_ is the minimum stomata conductance, *t* is time and *k* (*k*_i_ or *k*_d_) is the value calculated by the curve fitting function (fit) in Matlab (The Mathworks, Natick, MA, USA).

### Rubisco and Rubisco activase contents, Rubisco activity, total soluble protein and Chl assays

Leaf samples of 4 cm^2^ were collected, snap frozen and stored at −80°C until analysis. Samples were homogenized using an ice-cold mortar and pestle in 0.6 ml of extraction buffer (50 mM Bicine-NaOH pH 8.2, 20 mM MgCl_2_, 1 mM EDTA, 2mM benzamidine, 5 mM ɛ-aminocaproic acid, 50 mM 2-mercap-toethanol, 10 mM dithiothreitol, 1% (v/v) protease inhibitor cocktail (Sigma-Aldrich), and 1 mM phenylmethylsulphonyl fluoride). After rapid (45–60 s) grinding, samples were clarified via centrifugation at 4°C, 14 700 ***g*** for 1 min. The supernatant was used immediately to determine the initial and total activity of Rubisco via incorporation of ^14^CO_2_ into acid-stable products at 25°C (Parry *et al*., 1997; Carmo-Silva *et al*., 2017). This involved a reaction mixture containing 100 mM Bicine-NaOH pH 8.2, 20 mM MgCl_2_, 10 mM NaH^14^CO_2_ (9.25 kBq μmol^−1^), 2mM KH_2_PO_4_ and 0.6 mM RuBP. Assays of initial activity were started by the addition of 25 μl supernatant to the complete assay mixture, whilst total activity assays were started by addition of RuBP to the mixture 3 min after adding 25 μl of the supernatant, to allow full carbamylation of Rubisco in the presence of CO_2_ and Mg^2+^ before the assay. All reactions were quenched after 30 s by adding 100 μl of 10 M formic acid. Assay mixtures were dried at 90°C and 0.4 ml de-ionized water was added to re-dissolve the residue. Acid-stable ^14^C was determined by scintillation counting (Packard Tri-Carb; PerkinElmer, Waltham, MA, USA) with the addition of 3.6 ml of scintillation cocktail (Gold Star Quanta, Meridian Biotechnologies, Epsom, UK). The incubation time for total activity was tested to ensure accurate determination of total activity (Sharwood *et al*., 2016), and 3 min was found to be sufficient. Rubisco activation state was calculated as the ratio of initial to total activity. A 100 μl aliquot of the same supernatant was incubated at room temperature for 30 min with 100 μl of buffer containing 100 mM Bicine-NaOH pH 8.2, 20 mM MgCl_2_, 20 mM NaHCO_3_ and 1.2 mM (37 kBq μmol^−1^) [^14^C]CABP (carboxyarabintol-1,5-bisphosphate), and Rubisco content was determined via [^14^C]CABP binding (Sharwood *et al*., 2016).

Total soluble protein (TSP) was determined via a Bradford assay (Bradford, 1976). Chl determination followed the method described by Wintermans & de Mots (1965). A 20 μl aliquot of the homogenate was rapidly taken in duplicate before centrifugation and added to 480 μl ethanol, inverted to mix, and kept in the dark until all extractions were complete (Carmo-Silva *et al*., 2017). Chl content was determined by measuring absorbance using a microplate reader (SPECTROstar Nano; BMG LabTech, Aylesbury, UK).

To determine relative Rubisco activase content, an aliquot of the supernatant resulting from Rubisco analysis was mixed 1 : 1 with SDS-PAGE loading buffer (62.5 mM Tris-HCl, pH 6.8, 2% (w/v) SDS, 25% (v/v) glycerol, 0.01% bromophenol blue), mixed by pipetting and heated at 95°C for 4 min. Proteins were separated via SDS-PAGE (12% TGX gels, Bio-Rad), and transferred to a nitrocellulose membrane using a dry blotting system (iBlot2, ThermoFisher Scientific, Waltham, MA, USA) (Perdomo *et al*., 2018). Rubisco activase was detected using an antibody with broad specificity for both isoforms of the protein in higher plants (Feller *et al*., 1998), and a secondary fluoro-tagged antibody (IRDye800CW, Li-Cor Biosciences). Images were taken and protein amounts were quantified using a fluorescence imaging and analysis system (Odyssey FC; Li-Cor Biosciences). Due to uncertainty regarding the exact binding affinity of this antibody to cassava Rubisco activase, after densitometry of all samples, signal intensities were compared relative to the mean signal intensity of the entire dataset to provide relative quantification of the panel of cultivars.

### Cassava photosynthesis model and photosynthetic simulations

To estimate the influence of stomata and Rubisco response on dynamic photosynthesis rate, a cassava photosynthesis metabolic model was developed. The model was constructed based on three pre-existing models: the C_3_ photosynthesis model (Zhu *et al*., 2007), a simplified light reaction model; a Rubisco activase model (Mate *et al*., 1996; Zhu *et al*., 2013); and a dynamic stomatal conductance model (Vialet-Chabrand *et al*., 2017). The cassava model was implemented in MATLAB. The description of the equations used in the model are presented in Notes S1, and the code for this model is available at https://github.com/long-lab/Cassava_model.

The model was parameterized using cassava values of *V*_cmax_, *J*_max_, *k*_i_, *k*_d_, Ball–Berry slope and intercept. Each one of these parameters was calculated from photosynthetic measurements obtained in different cultivars of cassava (Table S1). Ball-Berry slope and intercept were calculated from light curves (*A*/PPFD curves) obtained for each cultivar (Table S2). For these curves, temperature and VPD were as described for *A/c_i_* curves, and [CO_2_] inside the chamber was kept at 400 μmol mol^−1^. The measured *V*_cmax_ was used as the maximum Rubisco activity in the C_3_ photosynthesis model. *A*, transpiration (*T*), *c*_i_ and *g*_s_ were estimated under a fluctuating light cycle (see later Fig. S8a). The predicted water use efficiency (WUE) was calculated dividing *A* by *T*.

### Statistical analysis

Differences between cultivars were tested by ANOVA or non-parametric methods (JMP Pro, version 12.0.1; SAS Institute, Cary, NC, USA). For all measured variables, normality was tested using the Shapiro-Wilk’s test and the homoscedasticity using Brown-Forsythe’s and Levene’s tests. When the data met the criteria for normality and homoscedasticity assumptions, one-way ANOVA followed by a pairwise comparison (*t*-test) was applied. When those criteria were violated, Wilcoxon’s nonparametric comparison was used. The threshold for statistical significance was *P*≤ 0.05. The data were analyzed using a completely randomized block design, split over 2 yr. The extent of correlation between steady-state variables was evaluated using Pearson’s correlation using the data of all cultivars.

## Results

### Cassava photosynthetic limitations under steady state

**Fig. 1:**
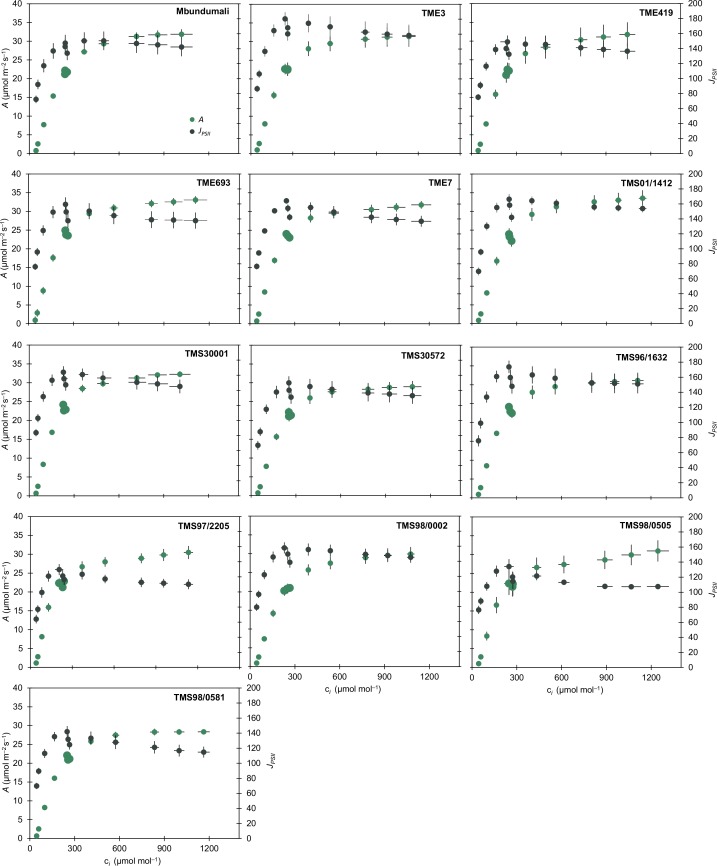
Response of light-saturated (*c*. 1500 μmol m^−2^ s^−1^) leaf carbon assimilation (*A*, μmol m^−2^ s^−1^; green) and of electron transport rate (*J*_PSII_, μmol m^−2^ s^−1^; black) to intracellular CO_2_ concentration (*c*_i_, μmol mol^−1^) in cassava cultivars. Symbols represent mean ± SE. *n* = 8, except for TMS98/0505 and TMS97/2205 where *n* = 4. Larger symbols indicate the operating point, which is the *c*_i_ achieved when the ambient [CO_2_] around the leaf is 400 μmol mol^−1^.

Light-saturated net leaf CO_2_ uptake (*A*_sat_) in cassava cultivars ranged from 20.3 to 24.8 μmol m^−2^ s^−1^, a total variation of 20% between cultivars (Table 1). A similar 20–24% range of variation was also observed for *V*_cmax_ and *J*_max_ calculated from the response of *A*_sat_ to *c*_i_, and *V*_cmax_ calculated from *c*_*c*_ (*V*_cmax,Cc_) (Table 1). Because estimation of *c*_c_ cannot be calculated by the variable *J* method when there is triose phosphate limitation due to the decrease in electron transport rate (Harley *et al*., 1992), values of *J*_max,Cc_ could not be calculated for cassava plants in this experiment. However, under high *c*_i_ the effect of *g*_m_ on *A*_sat_ is small (Harley *et al*, 1992). The operating efficiency of photosystem II (PSII) photochemistry (φPSII), which is usually correlated with the variation on *A*_sat_, varies *c*. 25% among cassava cultivars with an average of 0.22 across the cultivars (Fig. S2). The operating *c*_i_ values for all cultivars were below the transition in the *A/c*_i_ response from Rubisco limitation to electron transport limitation (Fig. 1), indicating that all of the cassava cultivars are Rubisco-limited at current atmospheric [CO_2_]. Stomatal conductance (*g*_s_) varied from 0.25 to 0.34 mol m^−2^ s^−1^ leading to a 26.5% of variation in intrinsic water use efficiency (*i*WUE) among cultivars (Table 1). Cultivar TMS97/2205 had the highest *i*WUE whereas TMS96/1632 and TMS01/1412 had the lowest *i*WUE values out of the cultivars surveyed (Table 1).

**Table 1:** Light-saturated leaf carbon assimilation (*A*_sat_, μmol m^−2^s^−1^), apparent maximum *in vivo* carboxylation rate at Rubisco (*V*_cmax_, μmol m^−2^ s^−1^), maximum carboxylation rate by Rubisco estimated from the partial pressure of CO_2_ inside the chloroplast (*V*_cmax,Cc_, μmol m^−2^ s^−1^), regeneration of ribulose-1,5-bisphosphate represented by electron transport rate (*J*_max_, μmol m^−2^ s^−1^), triose phosphate utilization (*V*_TPU_, μmol m^−2^ s^−1^), stomatal conductance (*g*_s_, mol m^−2^ s^−1^), intrinsic water use efficiency (*i*WUE, μmol CO_2_ mol H_2_O^−1^) and intracellular CO_2_ concentration at ambient [CO_2_] around the leaf, 400 μmol mol^−1^ (operating *c*_i_, μmol mol^−1^) in cassava cultivars.

Cultivar	*A*_sat_	*V*_cmax_	*V*_cmax, Cc_	*J*_max_	*V*_TPU_	*g*_s_	*i*WUE	Operating *c*_i_
Mbundumali	20.32 ± 1.05 b	100.12 ± 3.96b	124.81 ± 12.47 ab	169.41 ± 6.01 a	11.03 ± 0.43 ab	0.28 ± 0.02 abc	81.63 ± 5.84 ab	244.1 ± 9.45 ab
TME3	21.49 ± 1.78 abcd	101.83 ± 10.75 ab	156.73 ± 8.51 a	165.39 ± 14.52 ab	10.85 ± 0.89 ab	0.34 ± 0.02 a	71.66 ± 7.15 ab	257.43 ± 11.08 ab
TME419	22.17 ± 1.36 abcd	118.18 ± 6.90a	128.28 ± 5.98 b	183.86 ± 14.78 a	11.65 ± 0.81 ab	0.27 ± 0.02 bc	83.07 ± 4.48 ab	241.21 ± 7.69 ab
TME693	23.22 ± 1.27 abcd	110.29 ± 7.42 ab	133.19 ± 13.71 ab	171.3 ± 11.08 ab	11.43 ± 0.7 ab	0.33 ± 0.02 abc	75.41 ± 4.01 ab	252.96 ± 6.77 ab
TME7	24.61 ± 1.60 ac	104.82 ± 2.72 ab	140.44 ± 8.79 b	163.45 ± 7.47 ab	10.9 ± 0.36 a	0.34 ± 0.03 abc	74.22 ± 4.88 ab	254.91 ± 7.70 ab
TMS01/1412	24.81 ± 1.22 a	113.48 ± 3.83 a	135.62 ± 6.05 ab	175.88 ± 11.59 a	11.46 ± 0.68 a	0.32 ± 0.01 a	73.35 ± 4.85 b	255.77 ± 7.73 ab
TMS30001	22.95 ± 1.27 abcd	117.16 ± 6.87a	136.24 ± 7.21 ab	169.67 ± 5.73 a	11.13 ± 0.26a	0.28 ± 0.02 c	86.57 ± 4.52 ab	235.19 ± 7.33 b
TMS30572	20.81 ± 0.95 bd	95.24 ± 4.83 ab	120.63 ± 9.86 b	154.5 ± 10.56 ab	9.97 ± 0.50 ab	0.32 ± 0.04 abc	72.92 ± 6.13 ab	258.15 ± 9.35 ab
TMS96/1632	24.21 ± 1.23 ac	102.65 ± 6.75 ab	141.65 ± 9.45 ab	163.24 ± 14.20 ab	10.83 ± 0.70 b	0.33 ± 0.01 a	71.49 ± 3.38 b	258.63 ± 5.12 a
TMS97/2205	22.12 ± 0.37 b	100.33 ± 2.70 ab	122.47 ± 10.8 b	157.68 ± 7.50 ab	10.77 ± 0.51 ab	0.25 ± 0.02 c	93.48 ± 6.91 a	226.83 ± 10.84 b
TMS98/0002	21.92 ± 1.26 abcd	96.28 ± 2.23 b	119.68 ± 8.7 b	149.36 ± 10.15ab	10.5 ± 0.69 ab	0.29 ± 0.03 abc	78.96 ± 8.99 ab	249.02 ± 13.49 ab
TMS98/0505	21.49 ± 0.62 bc	97.06 ± 11.21 ab	132.68 ± 9.55 ab	161.15 ± 13.25 ab	10.45 ± 0.70 ab	0.3 ± 0.01 abc	78.7 ± 1.32 ab	250.41 ± 2.23 ab
TMS98/0581	23.11 ± 1.12 abcd	99.33 ± 4.36 ab	138.67 ± 7.6 ab	148.69 ± 4.63 b	9.88 ± 0.24 b	0.31 ± 0.02 abc	73.47 ± 5.31 ab	257.31 ± 8.53 ab

Values represent mean ± SE. *n* = 8. Different letters represent statistically significant differences (*P* < 0.05) among the cultivars.

**Fig. 2:**
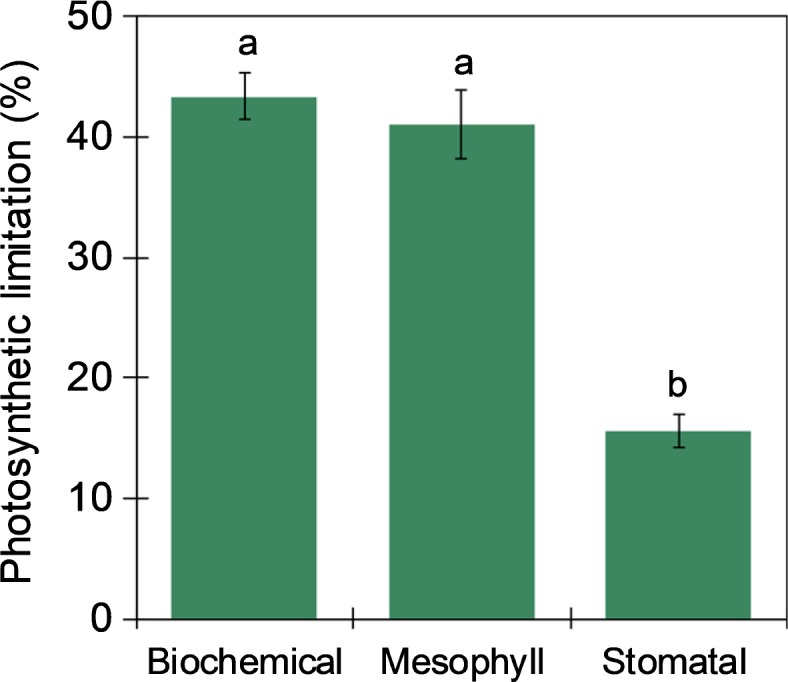
Relative biochemical, mesophyll and stomatal limitations at steady state in cassava. Total limitation is equal to 100%. Bars represent mean ± SE of all cultivars. Different letters represent statistically significant differences (*P*< 0.05) between different limitations.

Corroborating the data presented above, calculation of relative photosynthetic limitation by the method of Grassi & Magnani (2005) showed that, despite no significant differences among cultivars (Fig. S3), at current atmospheric [CO_2_] *in vivo* Rubisco activity accounted for about 43% of the total limitation across all cultivars, while stomatal conductance accounted for 16% (Fig. 2). Mesophyll conductance (*g*_m_) did not vary significantly among cultivars (Fig. S4). However, it did account for a similar proportion (i.e. 41%) of the total limitation to photosynthesis across cultivars in cassava (Fig. 2). Additionally, *g*_m_ was positively correlated to *A*_sat_ (*r* = 0.27, *P* = 0.042; Table S3).

For most cultivars, *A* did not increase significantly when measured at *c*_i_ higher than 700 μmol m^−2^ s^−1^ (Fig. 1). Except for TMS98/0505 and TMS97/2205, which increased photosynthesis by 7.7% and 5.1%, respectively, from a *c*_i_ of *c*. 800 μmol mol^−1^ to a *c*_i_ of *c*. 1250 μmol mol^−1^, all other cultivars showed, on average, only a 2.6% increase in photosynthesis under *c*_i_ higher than 700 μmol mol^−1^. The lack of increase in photosynthesis with an increase in *c*_i_ suggests that a TPU limitation is present in the majority of cassava cultivars evaluated in this study. This is shown by the observed concomitant reduction in *J*_PSII_ (6–16%) with increasing *c*_i_ (Fig. 1). There was a significant 15% variation in *V*_TPU_, which ranged from 9.9 to 11.65 μmol m^−2^ s^−1^ (Table 1). On average, *V*_TPU_ for cassava was 10.8 μmol m^−2^ s^−1^, suggesting a TPU utilization 44% above the average *A*_sat_ observed across the cassava cultivars.

Rubis cocontent, Rubisco initial, total and specific activity, and Rubisco activation state varied significantly among cultivars (Table 2). The variation in Rubisco content, and initial and total activity was positively correlated to *A*_sat_ (*r* = 0.46, *P* = 0.001; *r* = 0.36, *P* = 0.012; and *r* = 0.36, *P* = 0.011, respectively; Table S3). Rubisco content also correlated with *V*_cmax_ (*r* = 0.37, *P* = 0.009). Total Rubisco activase and fractions of α and β Rubisco activase iso-forms did not vary significantly (Table 2). Chl *a* (Chl*a*), *b* (Chl*b*), total Chl and the ratio of Chl*a*/Chl*b* showed significant differences among cultivars (Table S4). Of these, Chl*a*/Chl*b* ratio showed a significant correlation with *A*_sat_ (*r* = 0.30, *P* = 0.029; Table S1). Variation in total soluble protein content (TSP) and in the ratio of TSP to Chl (TSP/Chl) content between cultivars (Table S4) did not correlate with variation in *A*_sat_ (Table S3).

**Table 2:** Rubisco content (g m^−2^), Rubisco initial activity μmol CO_2_ m^−2^ s^−1^), Rubisco total activity μmol CO_2_ m^−2^ s^−1^), Rubisco activation state (%), Rubisco specific activity (μmol min^−1^ mg^−1^ rubisco), total Rubisco activase (Total Rca, relative signal intensity), fraction of α isoform of Rubisco activase (Rca % α, % of total) and fraction of β isoform of Rubisco activase (Rca % β, % of total) of cassava cultivars determined *in vitro*.

Cultivar	Rubisco content	Rubisco initial activity	Rubisco total activity	Rubisco activation state	Rubisco specific activity	Total Rca	Rca % α	Rca % β
Mbundumali	1.28 ± 0.1 cd	24.66 ± 3.15 bc	29.53 ± 2.74 bc	83 ± 3.5 ab	1.39 ± 0.06 ab	1.03 ± 0.03 a	0.41 ± 0.02 a	0.59 ± 0.02 a
TME3	1.85 ± 0.06 ab	39.78 ± 0.73 a	42.73 ± 0.55 a	93.2 ± 2.4 a	1.39 ± 0.03 a	1.02 ± 0.05 a	0.4 ± 0.02 a	0.6 ± 0.02 a
TME419	1.68 ± 0.07 abcd	35.78 ± 1.26 ab	40.24 ± 2.03 ab	89.2 ± 2.9 a	1.43 ± 0.03 a	1.02 ± 0.04a	0.4 ± 0.01 a	0.6 ± 0.01 a
TME693	1.72 ± 0.1 abc	35.94 ± 3.21 ab	42.43 ± 3.46 a	84.7 ± 3.6 ab	1.47± 0.04a	1.06 ± 0.03 a	0.41 ± 0.01 a	0.59 ± 0.01 a
TME7	1.79 ± 0.11 abc	33.16 ± 3.66 ab	37.11 ± 3.38 ab	88.9 ± 1.9 a	1.33 ± 0.02 ab	0.99 ± 0.05 a	0.4 ± 0.01 a	0.6 ± 0.01 a
TMS01/1412	1.59 ± 0.08 abcd	30.6 ± 1.93 ab	38.22 ± 1.1 ab	80.1 ± 4.6 ab	1.45 ± 0.03 a	0.98 ± 0.03 a	0.39 ± 0.02 a	0.61 ± 0.02 a
TMS30001	1.79 ± 0.08 ab	34.07 ± 3.17 ab	45.21 ± 3.14a	79.4 ± 2.3 ab	1.51 ± 0.07 a	0.98 ± 0.05 a	0.4 ± 0.02 a	0.6 ± 0.02 a
TMS30572	1.41 ± 0.11 acd	28.12 ± 3.09 abc	34.83 ± 2.78 abc	80.2 ± 3.2 ab	1.48 ± 0.02 a	0.99 ± 0.04 a	0.39 ± 0.01 a	0.61 ± 0.01 a
TMS96/1632	1.88 ± 0.13 b	38.5 ± 2.29 ab	43.28±1.38a	88.8 ± 2.4 ab	1.46 ± 0.05 a	0.97 ± 0.02 a	0.42 ± 0.02 a	0.58 ± 0.02 a
TMS97/2205	1.2 ± 0.12d	16.44 ± 2.39 c	23.17 ± 1.75c	70.5 ± 6.6 b	1.16 ± 0.03 b	0.96 ± 0.08 a	0.37 ± 0.01 a	0.63 ± 0.01 a
TMS98/0002	1.64 ± 0.13 abcd	33.4 ± 1.85 ab	39.36 ± 2.12 ab	85 ± 2.8 ab	1.45 ± 0.06 a	0.98 ± 0.05 a	0.36 ± 0.01 a	0.64 ± 0.01 a
TMS98/0505	1.46 ± 0.03 abcd	30.92 ± 2.25 ab	36.4 ± 0.81 abc	84.7 ± 4.2 ab	1.5 ± 0.01 a	1.02 ± 0.03 a	0.42 ± 0.02 a	0.58 ± 0.02 a
TMS98/0581	1.42 ± 0.08 acd	29.36 ± 2.1 abc	35.34 ± 1.86 abc	82.9 ± 2.7 ab	1.5 ± 0.04 a	1.03 ± 0.02 a	0.38 ± 0.01 a	0.62 ± 0.01 a

Values represent mean ± SE. *n* = 3−4. Different letters represent statistically significant differences (*P* < 0.05) among the cultivars. Total Rca, Rca % α and Rca % β did not present statistically significant differences.

### Dynamic photosynthesis and its limitations in cassava

**Fig. 3:**
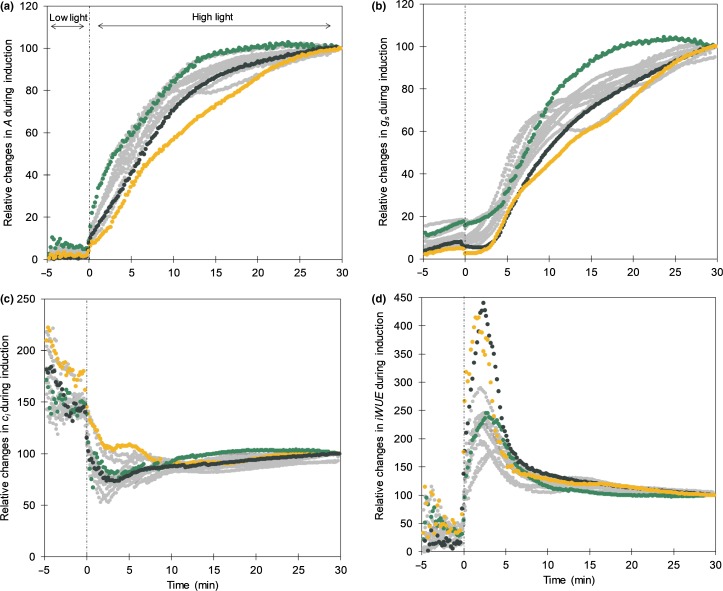
Changes in leaf carbon assimilation (*A*, μmol m^−2^ s^−1^) (a), stomatal conductance (*g*_s_ mol m^−2^ s^−1^) (b), internal CO_2_ concentration (*c*_i_, μmol m^−2^ s^−1^) (c) and intrinsic water use efficiency (*i*WUE, μmol CO_2_ mol H_2_O^−1^) (d) in cassava cultivars during photosynthetic induction. Relative values were calculated as the percentage of the value obtained after 30 min under high light. Low light was 50 μmol m^−2^ s^−1^ and high light 1500 μmol m^−2^ s^−1^ PPFD. Colored lines indicate the cultivars with contrasting responses: TME693 (yellow) and TMS98/0505 (green)) and cultivar TME7 (black), which were selected for further investigation. Gray lines represent the other 10 cultivars. Data represent means; *n* = 6 except for genotypes TMS98/0505 and TMS97/2205 where *n* = 3.

Induction of photosynthesis on transfer from deep shade (50 μmol m^−2^ s^−1^ PPFD) to high light (1500 μmol m^−2^ s^−1^ PPFD) was at significantly different rates across the cassava cultivars (*P*< 0.0001; Fig. 3a). TMS98/0505 showed the fastest induction, reaching 50% and 90% of the steady-state *A*_sat_ after 3 and 11 min, respectively. TME693 had the slowest induction rates with more than 10 and 21 min to reach, respectively, 50% and 90% of steady-state *A*_sat_ (Fig. 3a; Table 3). These differences in photosynthetic induction rates translated to a variation of 65% in CCF (Table 3), which correspond closely to stomatal opening, as represented by *g*_s_ (Fig. 3b; Table 3). Both stomatal conductance at the beginning of the induction (*g*_sT0_) and time to reach 50% of the final steady-state *g*_s_ (*T*_50gs_) had a significant correlation with CCF (*r* = −0.60, *P*< 0.0001 and *r* = 0.52, *P* < 0.0001). Despite the differences in induction rates, after 30 min the photosynthetic rates of all cultivars reached similar values to those obtained at steady-state (Fig. S5; Table 1). During photosynthetic induction, *i*WUE also varied among cultivars (Fig. 3d). During the first 5 min of induction, *i*WUE in TME7 was two-fold greater than in TMS 98/0505.

**Table 3:** Time to reach 50% of light-saturated leaf carbon assimilation (*T*_50A_, min), time to reach 90% of light-saturated leaf carbon assimilation (*T*_90A_, min), cumulative CO_2_ fixation in the first 5 min after photosynthesis induction (CCF, μmol CO_2_), stomatal conductance at the point of initiation of induction (*g*_s_*T*_0_, mol m^2^ s^−1^), and time to reach 50% of maximum stomatal conductance (*T*_50gs_, min) in cassava cultivars.

Cultivar	*T*_50A_	*T*_90A_	CCF	*g*_s_*T*_0_	*T*_50gs_
Mbundumali	4.2 ± 0.3 d	13.8 ± 0.6 bed	272 ± 20.7abcde	0.032 ± 0.006 abcd	8.08 ± 0.52 abc
TME3	6.1 ± 0.4 bc	15.5 ± 1.2 bed	187 ± 22.7 def	0.016 ± 0.003 de	7.7 ± 0.58 abc
TME419	4.6 ± 0.7 cd	14 ± 1.5 bed	291 ± 24.3 abc	0.027 ± 0.006 abcde	7.38 ± 1.20 bc
TME693	10.6 ± 1.4a	21.2 ± 1.1 a	122 ± 27.2 f	0.005 ± 0.004 e	9.48 ± 2.11 ab
TME7	6.4 ± 0.5 b	17.0 ± 1.6 abc	201 ± 35.4 cdef	0.019 ± 0.003 cde	10.58 ± 1.43 a
TMS01/1412	3.5 ± 0.5d	17.1 ± 1.5 abc	179 ± 31.6 ef	0.025 ± 0.006 bcde	5.75 ± 0.96 c
TMS30001	4.1 ± 0.5 d	17.1 ± 2.2 abc	280 ± 46.2 abcd	0.028 ± 0.006 abcde	6.21 ± 0.55 c
TMS30572	5.1 ± 0.7 bed	13.3 ± 1.6cd	262 ± 40.5abcde	0.020 ± 0.005 cde	7.67 ± 0.55 abc
TMS96/1632	4.5 ± 0.8 cd	17.8 ± 1.3 ab	276 ± 45.8abcde	0.045 ± 0.008 ab	10.33 ± 1.32 ab
TMS97/2205	3.1 ± 1.0 d	11.3 ± 0.5 d	333 ± 46.1 ab	0.054 ± 0.013 a	7.4 ± 0.92 abc
TMS98/0002	4.0 ± 0.7 d	16.4 ± 2.2 bcd	279 ± 41.2 abcd	0.032 ± 0.013 abcd	5.73 ± 0.67 c
TMS98/0505	3.1 ± 0.2 d	11.6 ± 0.7 d	349 ± 16.1 a	0.047 ± 0.003 abc	7.18 ± 1.78 abc
TMS98/0581	4.2 ± 0.6 d	17.6 ± 1.6ab	226 ± 33.9 bcde	0.034 ± 0.015 abcd	7.36 ± 0.69 bc

Values represent mean ± SE. *n* = 6 except for cultivars TMS98/0505 and TMS97/2205 where *n* = 3. Different letters represent statistically significant differences (*P* < 0.05) among the cultivars.

**Fig. 4:**
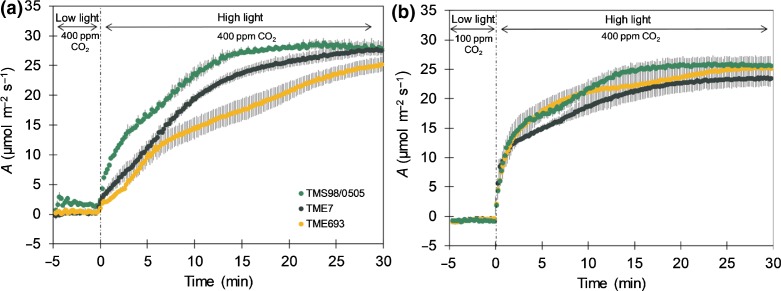
Leaf carbon assimilation (*A*, μmol m^−2^ s^−1^) in cassava during induction with CO_2_ concentration during low light phase set at 400 μmol mol^−1^ (a) or 100 μmol mol^−1^ (b). During the high light phase of the induction, CO_2_ concentration was maintained at 400 μmol mol^−1^ in both measurements. Comparison among cassava cultivars was based on the time to reach 50% of light-saturated leaf carbon assimilation (*T*_50A_, min), time to reach 90% of light-saturated leaf carbon assimilation (*T*_90A_, min), cumulative CO_2_ concentration in the first 5 min after photosynthesis induction (CCF) and stomatal conductance at the beginning of photosynthesis induction (*g*_sT0_, mol m^−2^ s^−1^) in both CO_2_ concentrations during the low light phase (c). Symbols in (a) and (b) represent mean ± SE. Values in (c) represent mean ± SE. *n* = 6 for TME693 and TME7; *n* = 3 for TMS98/0505. Different letters represent statistically significant differences (*P* < 0.05) among the cultivars.

The role of *g*_s_ on the speed of photosynthetic induction was investigated on the three selected cultivars by keeping the stomata open in low light, by reducing the chamber [CO_2_] to 100 μmol mol^−1^ during the low light phase. Here, induction in high light was far more rapid and did not differ between cultivars (Fig. 4c). Differences in the speed of induction were therefore due to differences in the speed of stomatal opening.

**Fig. 5:**
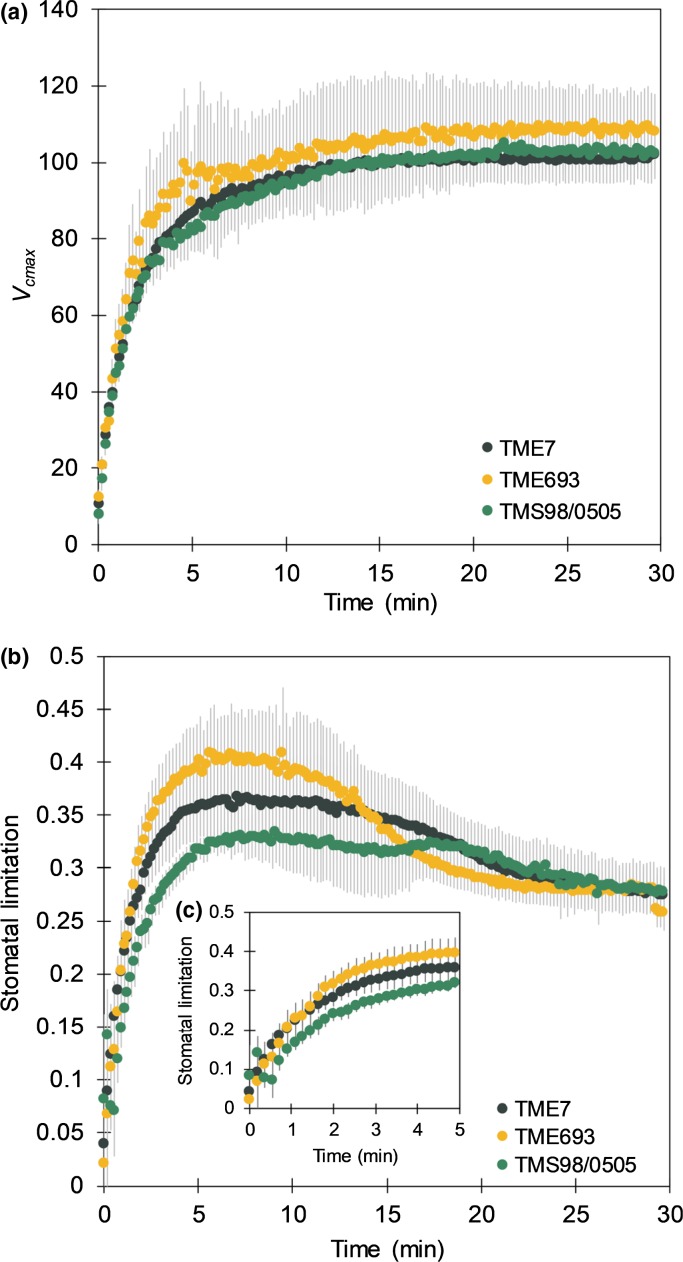
Maximum *in vivo* carboxylation rate by Rubisco (*V*_cmax_, μmol m^−2^ s^−1^) (a) and stomatal limitation during photosynthesis induction (b, c) in three cassava cultivars. Data represent mean ± SE. *n* = 3–4.

Biochemical and stomatal limitations during induction in cassava were further estimated by measuring photosynthetic induction under different CO_2_ concentrations. With these data, *A/c*_i_ curves were fit for different time points during the inductions (Fig. S6), and *V*_cmax_ and stomatal limitation were calculated (Fig. 5). The initial phase of the *A/c*_i_ curves increased with induction for the three cultivars, and no significant differences were observed (Fig. S6). This was reflected in a nonsignificant difference in *V*_cmax_ calculated for this phase across these cultivars (Fig. 5a), suggesting that Rubisco activity is not responsible for the differences observed during the induction. Nevertheless, the operating *c*_i_ in all three cultivars is in the Rubisco-limited part of the *A/c*_i_ curve throughout induction (Fig. S5), indicating that the induction response in cassava cultivars is overall Rubisco-limited. Stomatal limitation during induction is higher in TME693 than in TMS98/0505 (Fig. 5b), especially during the first 5 min (Fig. 5c) where there is a 20% difference (*P* = 0.034) between the two cultivars. Corroborating this, *c*_i_ during the first 5 min of induction under ambient [CO_2_] was 15.5% lower than the *c*_i_ at steady-state (Fig. 3c). Stomatal limitation in TME693 decreased after *c*. 15 min of induction and, after this period, it was similar to that of the other two cultivars (Fig. 5b).

On transfer from high light to shade, *A* decreased instantaneously but *g*_s_ required more than 20 min to reach steady state in all cassava cultivars (Fig. S7). Consistent with the differences in induction described above, TME693 showed low values of both rate constants for *g*_s_: the rate constant controlling increase on shade to sun transition (*k*_d_) and that controlling decrease on sun to shade transition (*k*_d_) (Table S1). By contrast, TMS01/1412, which had similar rates of photosynthesis induction to TMS98/0505 (Table 3; Fig. S5), showed the highest *k*_i_ and a high *k*_d_ (Table S1). However, a correspondence between *k*_d_ and *k*_i_ was not apparent across all cultivars.

### Model simulations

**Fig. 6:**
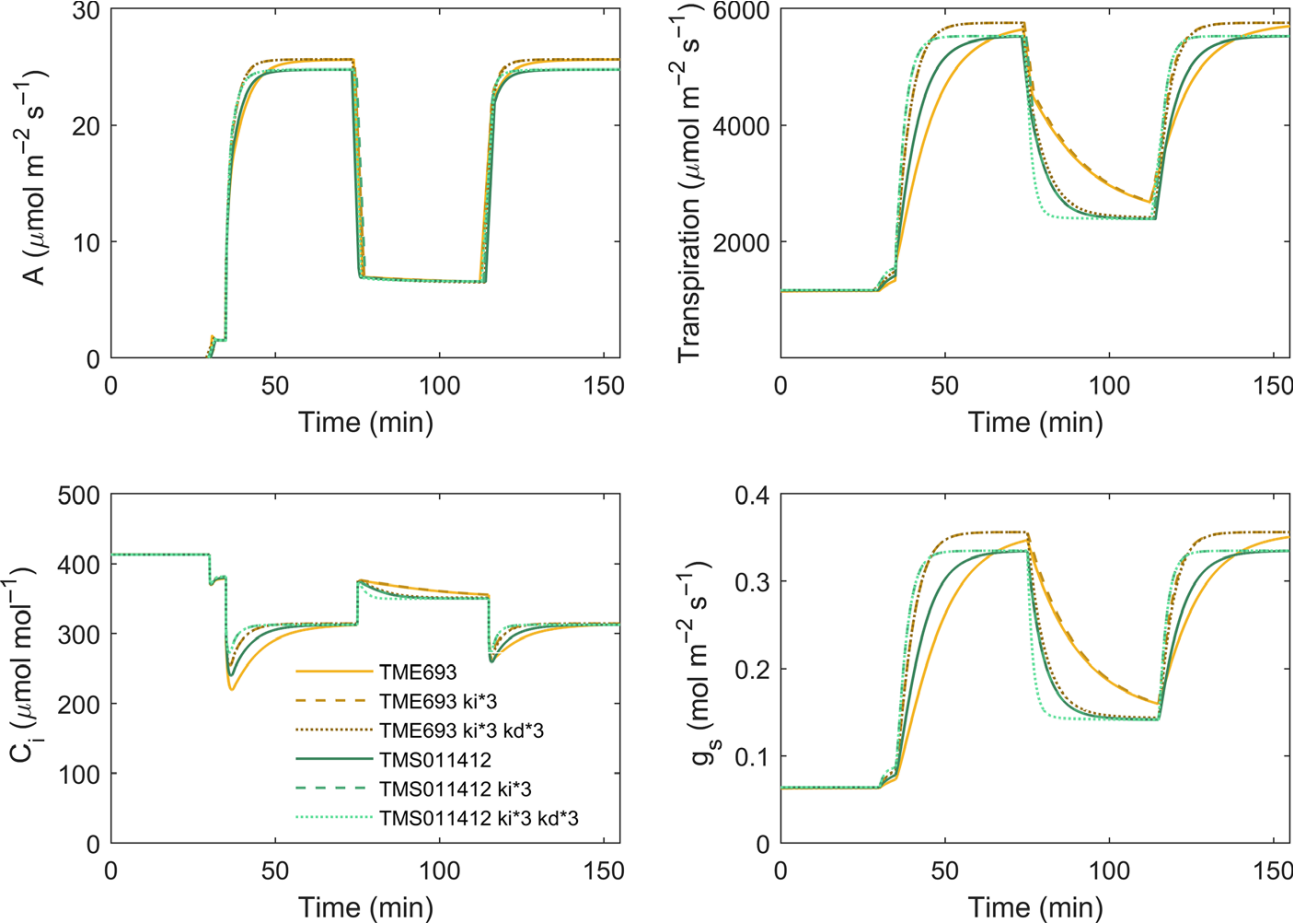
Model simulated carbon assimilation rate (*A*), transpiration rate (*T*), intercellular CO_2_ concentration (*c*_i_) and stomata conductance (*g*_s_) of cassava cultivars TME693 and TMS01/1412. Light in PPFD input is: 0 μmol m^2^ s^−1^ in the first 30 min, 50 μmol m^2^ s^−1^ from 30 to 35 min, 1500 μmol m^2^ s^−1^ from 35 to 75 min, 150μmol m^2^ s^−1^ from 75 to 115 min, and 1500 μmol m^−2^ s^−1^ from 115 to 155 min. Cultivar names followed by *k*_i_*3 represent the simulation with a three-fold increase in the rate of stomatal opening and *k*_i_*3 *k*_d_*3 a three-fold increase in rates of both stomatal opening and closure.

Values of *V*_cmax_, *J*_max_, *k*_i_, *k*_d_ and Ball–Berry parameters calculated from each cassava cultivar (Table S1) were used to simulate carbon assimilation and stomatal response in two contrasting cultivars, TME693 and TMS01/1412 (Fig. 6). These simulations were done considering the dynamic changes in Rubisco activation (DyRac) and dynamic stomatal conductance response (DyGs). Incorporation of these two variables improved the model performance as judged by an improved match to the measured induction curves (Fig. S8). The model showed that accelerating stomatal response three times would increase average *A* 11% for TME693 and 7% for TMS01/1412, during the first 10 min of induction (Fig. 6; Table S5). After 10 min of induction, and during low- and high-light phases, there was no significant impact (i.e. < 3%) of acceleration of stomatal response on *A*. However, acceleration in stomatal response decreased WUE *c*. 15% in TME693 over the first 30 min of photosynthesis induction. For TMS01/1412, this reduction was *c*. 12% during the first 20 min of induction. There was also a decrease in WUE by 8% during the first 20 min of high light for both cultivars. However, WUE increased by 20% in TME693 and by 13% in TMS01/1412 during the first 20–30 min of low light, by accelerating the speed of decline in *g*_s_ three-fold (Fig. 6; Table S5).

In a simulated cycle of low and high light applied to all cultivars (Fig. S9) there was an average of 13% loss of carbon assimilation and 5% of WUE resulting from the lags in stomatal response. Accelerating stomata opening and closure speed three times offset 6% of this carbon loss, and 2% of WUE (Fig. S9b).

## Discussion

Overcoming photosynthetic limitations to improve photosynthetic efficiency at the leaf level has resulted in some large demonstrated increases in field crop productivity and WUE (Kromdijk *et al*., 2016; Glowacka *et al*., 2018; Simkin *et al*., 2019; South *et al*., 2019). Previous focus has been overwhelmingly on light-saturated steady-state photosynthesis. However, in field crop canopies, half of carbon gain is under conditions where photosynthesis is light-limited and most leaves are rarely under steady-state light (Zhu *et al*., 2004; Taylor & Long, 2017; Papanatsiou *et al*., 2019). While steady-state measurements are valuable for quantification of biochemical limitations *in vivo* (Long & Bernacchi, 2003), dynamic measurements provide insight into the more frequent field condition, particularly in crops canopies, of how leaves respond to fluctuating light (Way & Pearcy, 2012). Indeed, variation between cassava cultivars in carbon assimilation under nonsteadystate conditions was three times that under steady-state conditions (Tables 1, 3), identifying important new traits and therefore opportunities for selection in improving cassava photosynthetic efficiency and yield potential. With the recent advances in genomic resources for cassava (Bredeson *et al*., 2016) and the development of large-scale breeding efforts (Maxmen, 2019), the incorporation of such traits into new cassava varieties may be accelerated to increase yield potential.

### Biochemical and mesophyll limitations play a major role in photosynthesis under steady state

Similar to other C_3_ crops (Xiong *et al*., 2018), in cassava biochemical limitation at steady-state was 43% of the total photosynthetic limitation (Fig. 2). *In vivo* Rubisco activity, not regeneration of RuBP, accounted for this biochemical limitation under the current atmospheric [CO_2_], as operating *c*_i_ for all culti-vars was below the transition from Rubisco to electron transport limitation, representing RubP regeneration limitation (Fig. 1). On average, Rubisco content in cassava was 1.6 g m^−2^ (Table 2). This is low compared to 3 g m^−2^ for wheat and 2.6 g m^−2^ for rice, under similar conditions of good nutrition (Theobald *et al*., 1998; Masumoto *et al*., 2005). Although the CO_2_ specificity of Rubisco in cassava is slightly higher (*S*_c/o_ at 25°C = 105.4 ± 1.8) than in both rice and wheat (*S*_c/o_ at 25°C = 101 ± 2 and 100 ± 1.1, respectively), its carboxylation efficiency of Rubisco (*k*_cat_^c^/*k*_c_^air^) is *c*. 30% lower (Orr *et al*., 2016). Lower content and efficiency would explain the lower *V*_cmax_ in cassava (Table 1) compared to elite cultivars of soybean, wheat and rice (Masumoto *et al*., 2005; Driever *et al*., 2014; Koester *et al*., 2014). This difference between cassava and these other C_3_ crops suggests that strategies proposed to improve Rubisco efficiency and quantity would have particular value with this crop (Parry *et al*., 2007; Whitney *et al*., 2011; Carmo-Silva *et al*., 2015). The 20% between-cultivar variation in *V*_cmax_ found here, although less than the 35% and 55% observed in rice and soybean, respectively (Gu *et al*., 2012; Koester *et al*., 2014), still provides a basis for breeding a significant improvement in photosynthetic efficiency. Additionally, the advance in genomic resources can help to target overcoming the low genetic variation in cassava in Sub-Saharan Africa, which has been a consequence of the limited introductions into Africa (Bredeson *et al*., 2016).

Despite some uncertainties regarding the methods for *g*_m_ estimation, the limitation to steady-state photosynthesis imposed by mesophyll conductance in this study approached that imposed by assimilation within the chloroplast (*c*. 41%, Fig. 2). This is more than double the limitation imposed by stomata (Fig. 2). Increasing *g*_m_ is an attractive target for breeding or bioengineering, because it can increase photosynthesis without increasing transpiration (Flexas *et al*., 2008; Zhu *et al*., 2010). An extensive survey of South American cultivars showed that differences in photosynthesis, biomass and yield were closely associated with variation in *g*_m_ (El-Sharkawy & Cock, 1990; El-Sharkawy *et al*., 1990, 2008). This is consistent with the correlation between *g*_m_ and *A*_sat_ found here for African cultivars (Table S3). However, there is no evidence that g_m_ has been increased with breeding, with no significant difference between g_m_ in landraces and improved lines (*F* = 0.02; *P* = 0.889) suggesting that efforts to increase *g*_m_ in cassava might lead to a significant improvement in photosynthetic rate in this crop.

Simulations have shown that increasing either *V*_cmax_ or *g*_m_ could compensate for up to a 40% decrease in stomatal conductance to water vapor (*g*_sw_) (Flexas *et al*., 2016). This would allow a cultivar to maintain the same *A*_sat_ while using 40% less water, that is a 40% increase in *i*WUE. Although manipulations in *g*_m_ have been found to affect *g*_s_ negatively in some other species (Hanba *et al*., 2004; Flexas *et al*., 2006), and *g*_m_ showed a strong positive correlation with *g*_s_ in soybean (Tomeo & Rosenthal, 2017), these two parameters were not significantly correlated in cassava (*r* = 0.14, *P* = 0.280; Table S3). A similar lack of correlation was also found across cultivars of wheat, supporting the contention that improved *g*_m_ may be selected without impacting *g*_s_ (Jahan *et al*., 2014; Barbour *et al*., 2016). In cassava this would not only increase *A*_sat_ under optimal conditions, but increase its resilience to the frequent and increasing droughts affecting the major growing regions of Sub-Saharan Africa (Tadele, 2018).

### Low capacity of TPU may limit photosynthetic improvements

While Rubisco and mesophyll conductance are the major limitations found in cassava under current atmospheric conditions, TPU limitation, which reflects the plant’s ability to convert triose phosphates into sucrose and starch (Sharkey, 1985), can represent a major hurdle for improving photosynthesis in this crop. Eleven of the 13 cassava cultivars evaluated showed TPU limitation, at an *A*_sat_ only slightly higher than the measured *A*_sat_ at the current ambient [CO_2_]. This was evident as a lack of any increase in *A*_sat_ when *c*_i_ exceeded 700 μmol m^−2^ s^−1^ and an observed decline in *J*_PSII_ with increasing *c*_i_ (Fig. 1) (Sharkey, 1985; Long & Bernacchi, 2003). The average *V*_TPU_ across the cassava cultivars was 10.8 μmol m^−2^ s^−1^ and only sufficient to support a maximum *A*_sat_ of 32 μmol m^−2^ s^−1^. Therefore, the maximum improvement in photosynthesis that could be bred or bioengineered could not exceed 33% without simultaneous improvement of *V*_TPU_. *V*_TPU_ values here were similar to those found in a more limited subset of African cassava cultivars (De Souza & Long, 2018), and 25.542% lower than in rice, wheat and rye (Wullschleger, 1993; Jaikumar *et al*., 2013). Low rates of *V*_TPU_ can be associated with reduced sink strength for growth or storage, or with insufficient capacity to synthesize sucrose and starch in the leaf (Long & Bernacchi, 2003; Sharkey *et al*., 2007). Cassava produces large tuberous roots. Thus, it is not expected that a reduced sink strength would cause its low *V*_TPU_. However, tuberous roots start to develop only after 2–3 months of planting (De Souza *et al*., 2017), and it is known that the response of cassava varies with age, especially between pretuberous and tuberous growing phases (Gleadow *et al*., 2016). Our measurements were performed before 2 months, which would indicate a limitation during the plant’s establishment phase (De Souza & Long, 2018). Nevertheless, failure to fully utilize photosynthetic potential, even before storage roots form, will be at the cost of canopy and root expansion during the critical establishment phase of the crop. Suggested strategies would be upregulation of ADPglucose pyrophosporylase in roots, and ADPglucose pyrophosphatase in leaves to enhance sucrose and starch synthesis (Ihemere *et al*., 2006; Jonik *et al*., 2012; Yang *et al*., 2016; Sonnewald & Fernie, 2018). These strategies may increase *V*_TPU_ in cassava, and allow greater bioengineered or bred increases in photosynthesis.

### Slow stomatal conductance limits carbon fixation during light fluctuations

After the transition from deep shade or low light to high light, cassava takes *c*. 20 min to reach photosynthetic rates comparable to steady state (Figs 3a, S5, S7). CCF over first 5 min varied by 286%, from 122 μmol CO_2_ assimilated for TME693 to 349 μmol for TMS98/0505 (Table 3). What limits CCF in cassava? In tobacco, rice, soybean and wheat, Rubisco activation is the major limitation to induction (Hammond *et al*., 1998; Yamori *et al*., 2012; Soleh *et al*., 2016; Taylor & Long, 2017; Salter *et al*., 2019), whereas in cassava, it is the rate of stomatal opening (Figs 3, 5). While *V*_cmax_ during induction was similar between the contrasting cultivars, stomatal limitation in the first 5 min varied substantially (Fig. 5). When stomatal limitation was effectively removed by artificially lowering the chamber [CO_2_] during shade, differences between cultivars in the speed of induction were eliminated (Fig. 4).

The rate constant for *g*_s_ increase varied 47% between cultivars with an average value of 9.8 min (Table S1). By definition, the higher the *k*_i_ the slower the rise in *g*_s_. The measured *k*_i_ values for cassava were similar to those reported for tomato, wheat and common bean, but were 11 times higher than for rice, and three times higher than for maize (McAusland *et al*., 2016). Slow stomatal opening during induction can significantly affect CO_2_ uptake and have a cumulative effect over a day and over a growing season, lowering yields (Reynolds *et al*., 1994; Fisher *et al*., 1998; Lawson & Blatt, 2014; Taylor & Long, 2017). Therefore, cultivars with an increased *k*_i_, or any genetic manipulation that would allow acceleration of opening would benefit photosynthesis in cassava. Our simulations showed that with a three-fold acceleration of *k*_i_, it is possible to increase photosynthetic carbon gain by 7–11% during the first 10 min after induction from deep shade (Fig. 6; Table S5). The large, almost three-fold, differences found between cultivars during induction (Table 3) could therefore be exploited to improve cassava yield potential. Compared to just a 20% variation in steady-state photosynthesis (Table 1), this emphasizes nonsteady-state photosynthesis as an overlooked trait for improving cassava productivity.

Accelerating stomatal opening can cause a pronounced decrease in WUE. This is because rate of increase in transpiration through the stomata is higher than the rate of increase in CO_2_ assimilation due to the intrinsic differences in water and CO_2_ concentration gradients between the intracellular spaces and the external atmosphere (Lawson & Blatt, 2014). To counterbalance the decrease of WUE when *k*_i_ is accelerated (Fig. 6; Table S5), it is also necessary to accelerate the rate of stomatal closing on sun to shade transitions. For the majority of cassava cultivars, the rate constants for *g*_s_ decrease (*k*_d_) were lower than for *k*_i_ (Table S1), indicating that cassava stomata are faster to close than to open. Even so, the average value of *k*_d_ in cassava is higher than for many other crops such as rice, maize, common beans, oat, tomato, sorghum and wheat (McAusland *et al*., 2016). Our modeling showed that a three-fold increase in *k*_i_ and *k*_d_ would increase WUE by 16–20% during the transition from high to low light depending on genotype (Fig. 6; Table S5). Given a cycle of fluctuations in light similar to that observed in lower layers of the canopy, this increase in *k*_i_ and *k*_d_ would increase daily carbon assimilation by 6% without a significant change in WUE (Fig. S9). Importantly, 6% would be the minimum gain in productivity, as before canopy closure this would have a positive feedback by creating more leaf and, in turn, more canopy carbon gain. Thus, over the full growth cycle of cassava of 10–12 months (Lebot, 2009), a substantially higher gain in carbon would be expected while maintaining the current WUE.

Despite low genetic variability in the cassava of Sub-Saharan Africa, this study has identified opportunities to substantially improve photosynthetic carbon gain and increase WUE, particularly by giving attention to nonsteady-state photosynthetic traits.

## Supporting Information

**Fig. S1** Leaf, stem tuberous roots and total biomass of 45 d old cassava cultivars.

**Fig. S2** Operating efficiency of PSII photochemistry at ambient [CO_2_] in cassava cultivars.

**Fig. S3** Relative biochemical, mesophyll and stomatal limitations under steady state in cassava cultivars.

**Fig. S4** Mesophyll conductance in cassava cultivars.

**Fig. S5** Changes in carbon assimilation during photosynthesis induction in cassava.

**Fig. S6** Dynamic *A/ci* curves for three cassava cultivars.

**Fig. S7** Changes in leaf carbon assimilation, stomatal conductance and intrinsic water use efficiency during light fluctuation in cassava.

**Fig. S8** Simulated carbon assimilation rate, transpiration rate, intercellular CO_2_ concentration and stomata conductance in cassava.

**Fig. S9** Light input used in the cassava model simulations and results from simulated influence of dynamic stomata and dynamic Rubisco on carbon assimilation and water use efficiency.

**Notes S1** Cassava leaf photosynthesis and transpiration model description.

**Table S1** Input parameters of cassava model of leaf photo synthesis and transpiration.

**Table S2** Dataset of light curves from cassava used for calculations of Ball-Berry parameters.

**Table S3** Matrix of Pearson’s correlation coefficients and their *P*-values.

**Table S4** Chl contents, total soluble protein content, fraction of total soluble protein present as Rubisco, and ratio of total soluble protein to Chl content in cassava cultivars.

**Table S5** The effect of a three-fold acceleration of stomatal response on carbon assimilation rate and water use efficiency in cassava cultivars.
