# Supplementary material for: Photosynthesis across African cassava germplasm is limited by Rubisco and mesophyll conductance at steady state, but by stomatal conductance in fluctuating light
Source: New Phytol. 2019 Aug 25;225(6):2498–512. doi: 10.1111/nph.16142 (PMC7065220; doi:10.1111/nph.16142)
Supplement: Supplementary file 1 [file NP-2019-NPH-16142-s1.pdf]

New Phytologist

SUPPORTING INFORMATION FOR

PHOTOSYNTHESIS ACROSS AFRICAN CASSAVA GERMPLASM IS LIMITED BY RUBISCO AND MESOPHYLL CONDUCTANCE AT STEADY-STATE, BUT BY STOMATAL CONDUCTANCE IN FLUCTUATING LIGHT

Amanda P. De Souza[1], Yu Wang[1], Douglas Orr[2], Elizabete Carmo-Silva[2], Stephen P. Long[1,2*]

[1] Carl R Woese Institute for Genomic Biology, University of Illinois at Urbana-Champaign, Urbana, IL, 61801, USA

[2] Lancaster Environment Centre, Lancaster University, Lancaster, LA1 4YQ, UK

The following Supporting Information is available for this article:

Figures S1-S9

Tables S1- S5

Notes S1

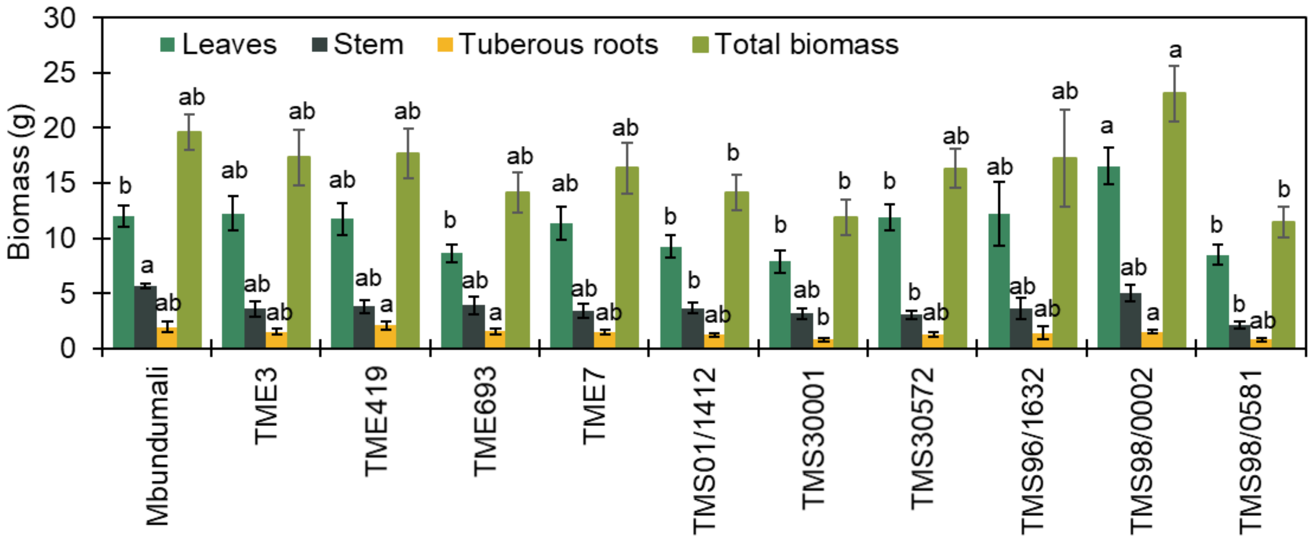

Figure S1. Leaf, stem, tuberous roots and total biomass of cassava plants. Bars represent mean ± SE. *n* = 4. Different letters represent statistically significant differences (*P*<0.05) among the cultivars. Biomass was measured at 45 days-old plants. Material was collected and oven dried at 60°C until constant weight.

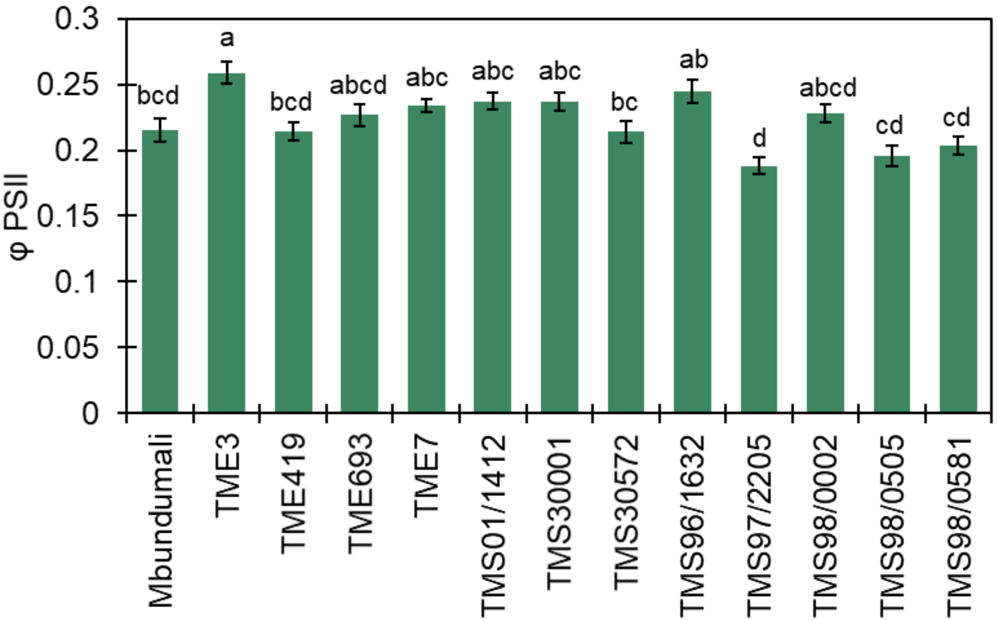

Figure S2. Operating efficiency of PSII photochemistry (φPSII) in cassava cultivars at ambient [CO2] of 400 μmol mol^{-1}. Bars represent mean ± SE. *n* = 4-8. Different letters represent statistically significant differences (*P*<0.05) among the cultivars.

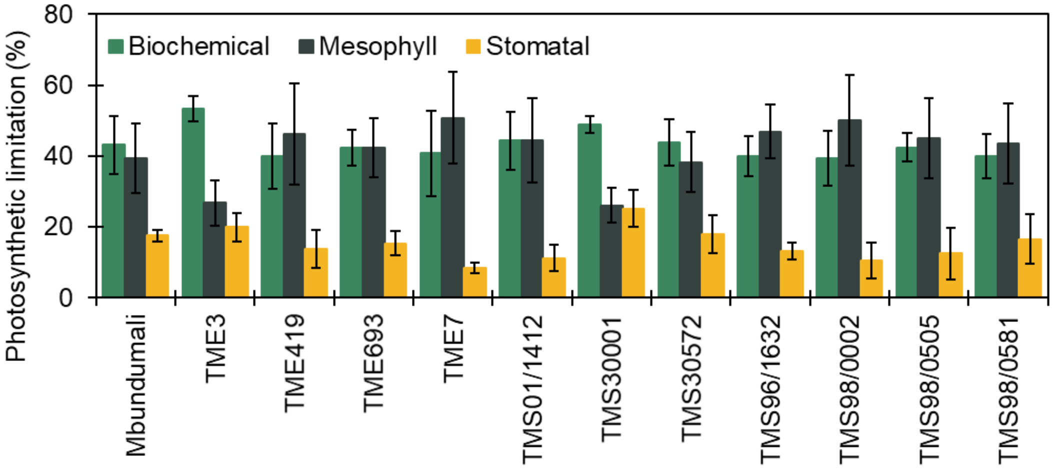

Figure S3. Relative biochemical, mesophyll and stomatal limitations under steady state in cassava cultivars. Bars represent mean ± SE. *n* = 4-8. There is no statistically significant differences (*P*<0.05) among cultivars.

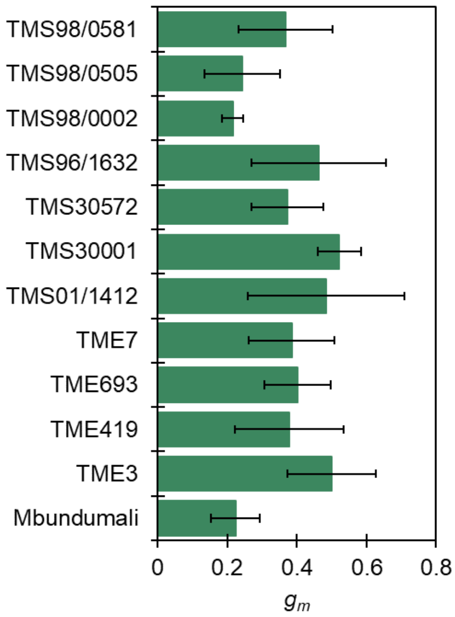

Figure S4. Mesophyll conductance (g_m, mol $m^{-2}s^{-1}$ bar^{-1}) in cassava cultivars. Bars represent mean ± SE. n = 4-8. There is no statistically significant differences (P<0.05) among cultivars.

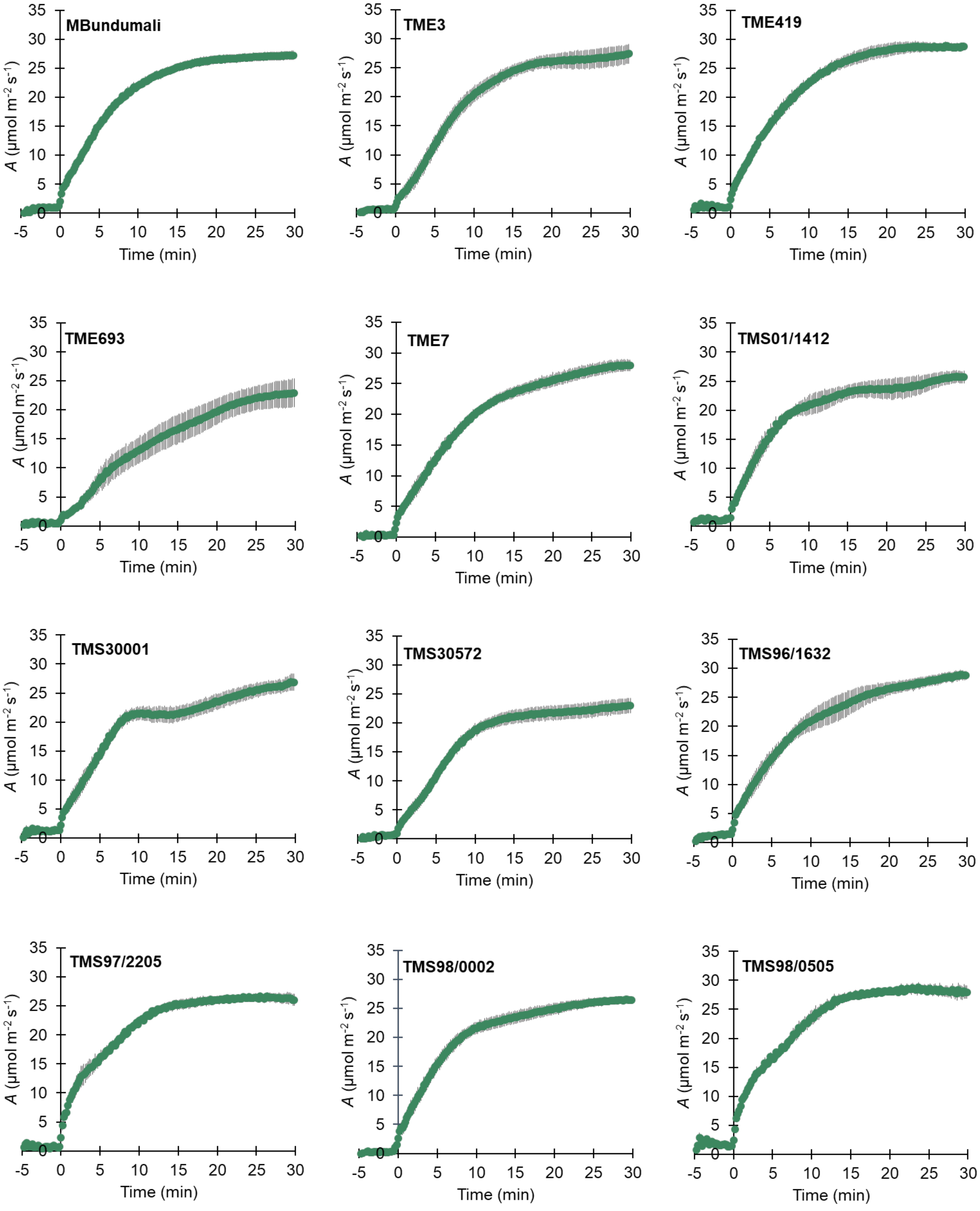

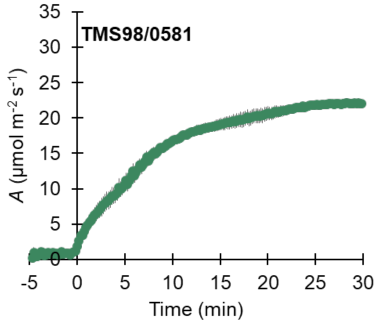

Figure S5. Changes in carbon assimilation (*A*, µmol m^{-2} s^{-1}) during photosynthesis induction for cassava cultivars. Data represent mean ± SE. *n*=6, except for TMS 98/0505 and TMS97/2205 where *n*=3.

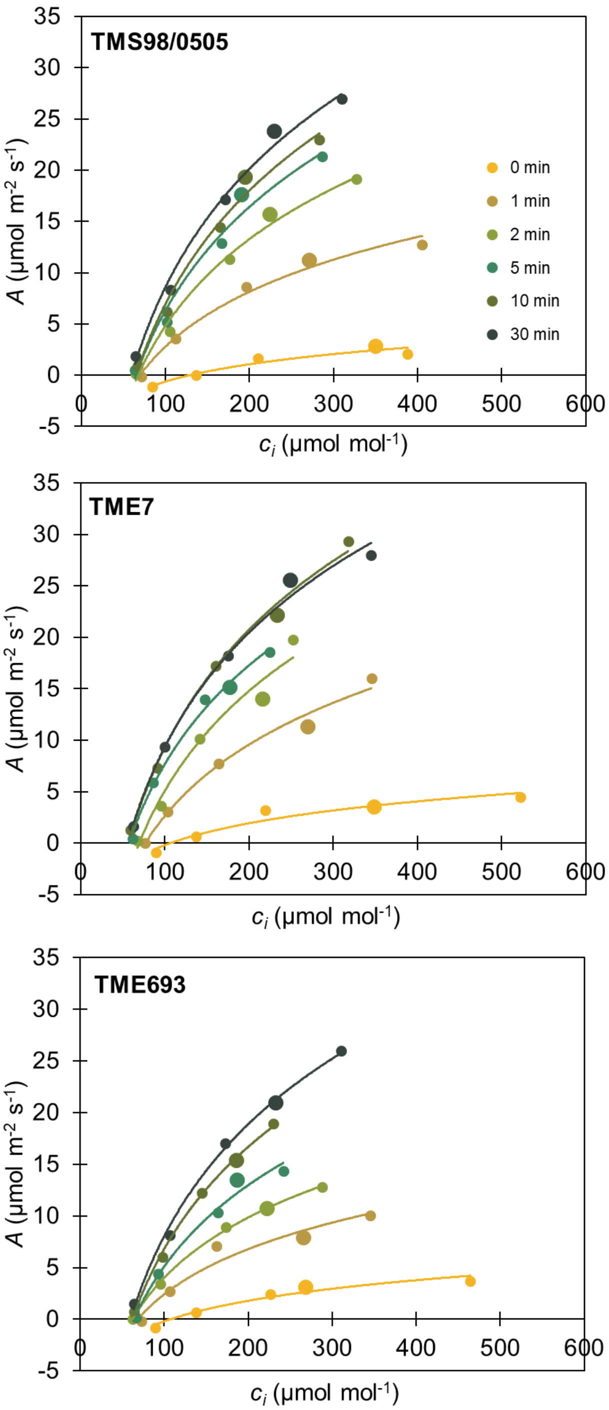

Figure S6. Response of leaf carbon assimilation (*A*, μmol m^{-2} s^{-1}) to internal CO_2 concentration (c_i, μmol mol^{-1}) in six time points during photosynthesis induction for three cassava cultivars (TMS98/0505, TME7 and TME693). These curves are typically referred as 'dynamic A/c_i curves'.*n*=4. Larger symbols indicate the operating c_i.

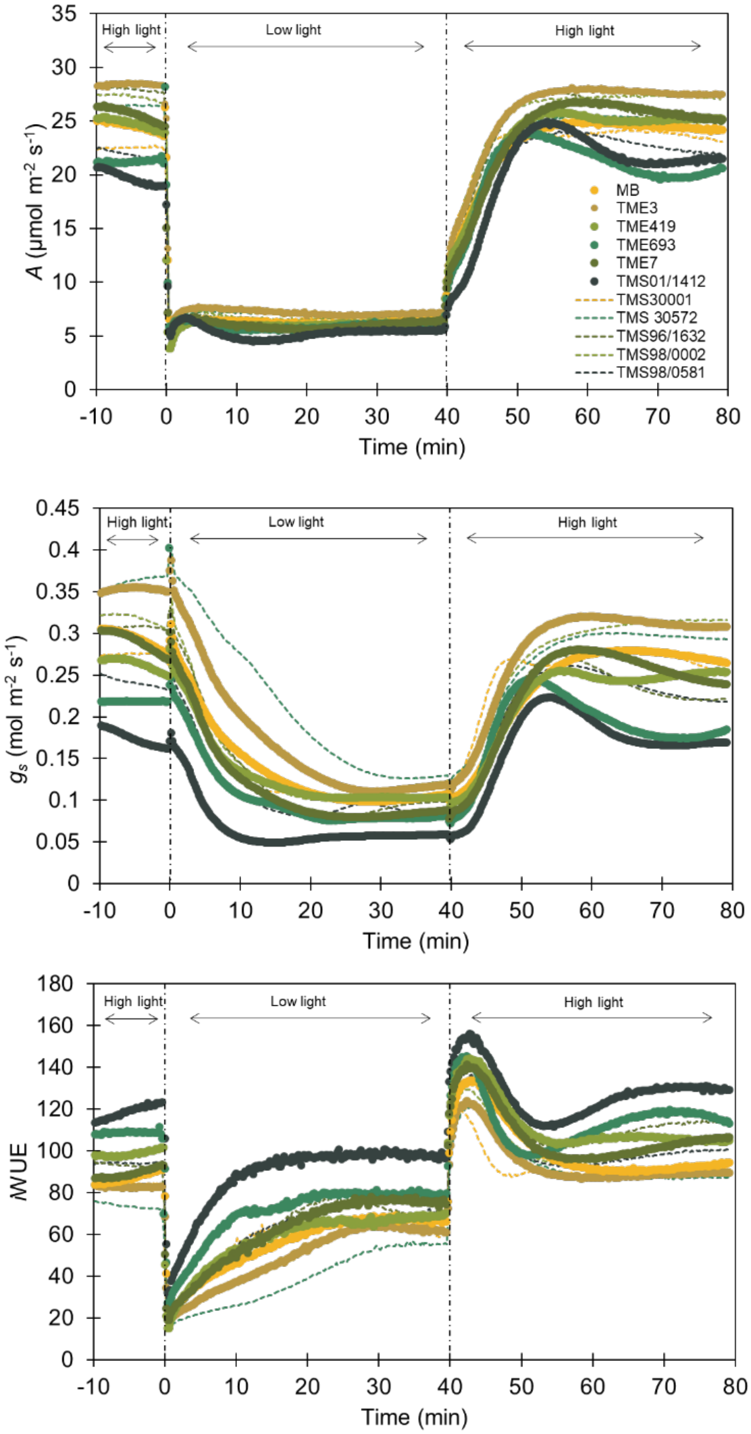

Figure S7. Changes in leaf carbon assimilation (*A*, µmol m^{-2} s^{-1}), stomatal conductance (g_s, mol m^{-2}s^{-1}), and intrinsic water efficiency (*i*WUE, µmol CO_2 mol H_2O^{-1}) during photosynthesis relaxation (from high to low light) followed by photosynthesis induction (from low to high light) in cassava cultivars. During low light phase, light was set to 150 µmol m^{-2}s^{-1} PPFD, and during high light phase, it was set to 1500 µmol m^{-2}s^{-1} PPFD. Data represent mean. *n*= 3-4.

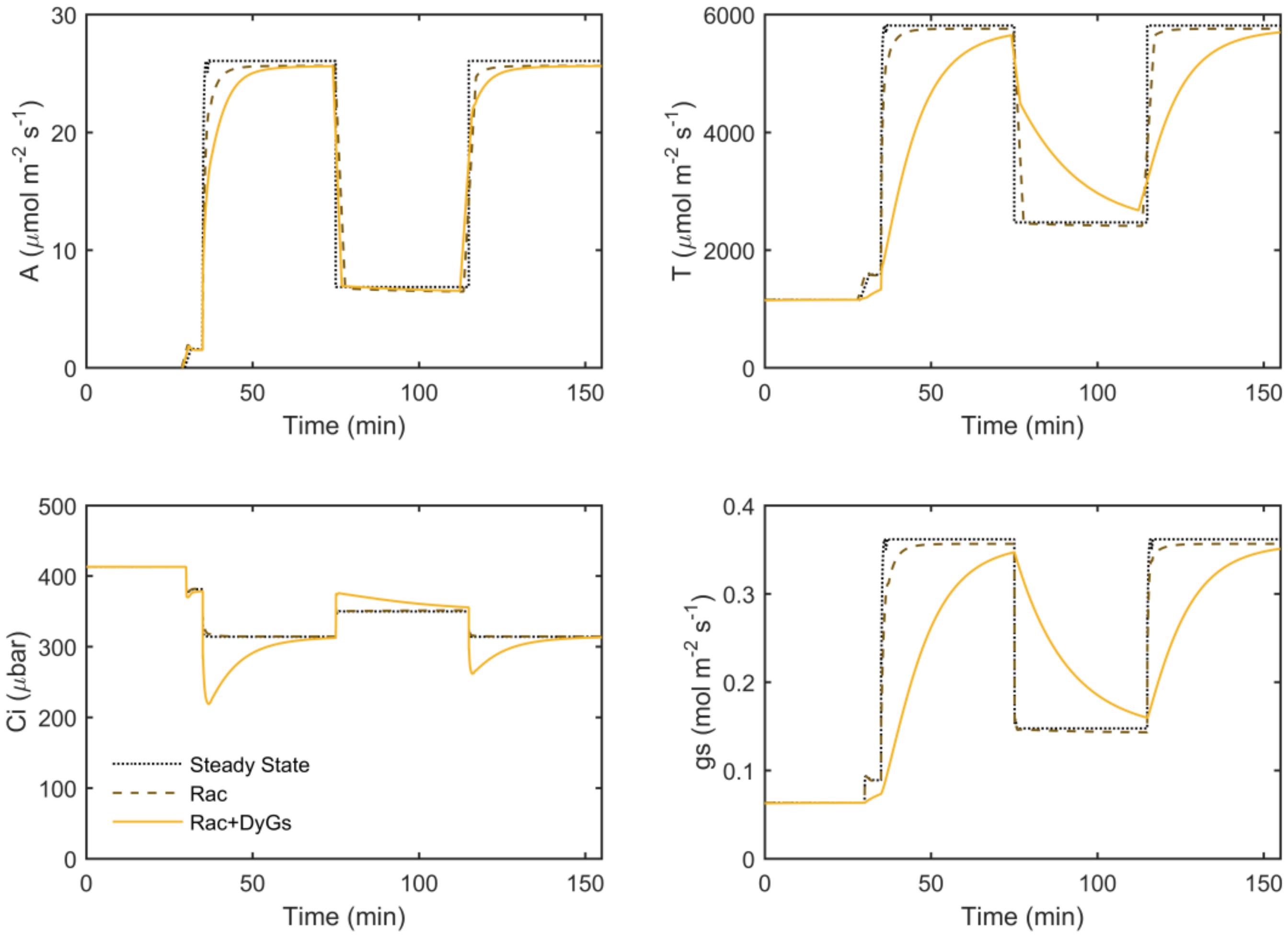

Figure S8. Simulated carbon assimilation rate (*A*), transpiration rate (*T*), intercellular CO_2 concentration (c_i), and stomata conductance (g_s) of cassava. Black dot line assumes that the Rubisco activity is constant and stomata conductance is in steady state. Dashed line considers that Rubisco activation changes under varying light condition (DyRac). Yellow line considers Rubisco activation changes and dynamic stomata response (DyRac+DyGs).

(a)

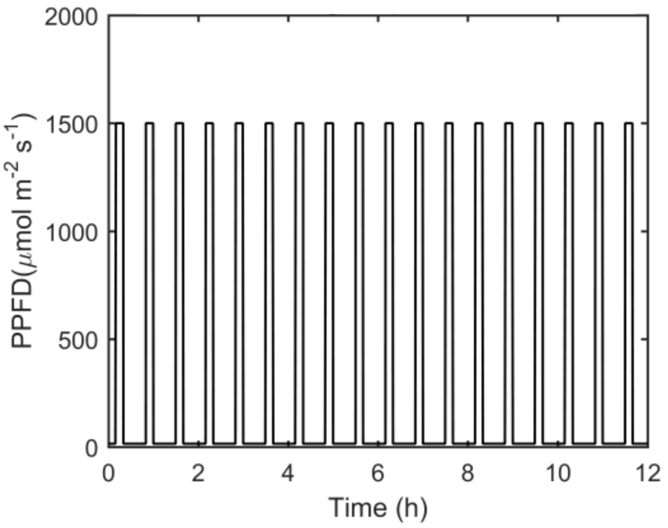

(b)

Cultivar	with DyRac		with DyRac and DyGs		with DyRac and DyGs + k_i*3k_d*3	
	A	*WUE*	*A*	*WUE*	*A*	*WUE*
**Mbundumali**	0.97	0.98	0.86	0.96	0.93	0.98
**TME3**	0.97	0.98	0.89	0.91	0.94	0.95
**TME419**	0.97	0.98	0.86	0.96	0.93	0.98
**TME693**	0.97	0.98	0.87	0.82	0.92	0.89
**TME7**	0.97	0.98	0.85	0.96	0.93	0.98
**TMS01/1412**	0.97	0.98	0.89	0.95	0.95	0.97
**TMS30001**	0.97	0.99	0.76	1.00	0.90	1.01
**TMS30572**	0.97	0.98	0.89	0.82	0.93	0.89
**TMS96/1632**	0.97	0.98	0.91	0.98	0.95	0.99
**TMS98/0002**	0.97	0.98	0.89	1.02	0.94	1.01
**TMS98/0581**	0.97	0.98	0.88	1.01	0.94	1.00
**AVERAGE**	0.97	0.98	0.87	0.95	0.93	0.97

Figure S9. Light input used in the cassava model simulations and its results. **(a)** a high-low light cycle considering low light as 15 μmol m^{-2} s^{-1} PPDF for 30 min, and high light as 1500 μmol m^{-2} s^{-1} PPFD for 10 min; **(b)** Results from simulated influence of dynamic stomata (DyGs) and dynamic Rubisco (DyRac) on carbon assimilation rate (*A*, μmol m^{-2}s^{-1}) and water use efficiency (*WUE*, μmol CO_2 mol H_2O^{-1}). Results were normalized by steady state predictions.

Table S1. Input parameters of cassava model of leaf photosynthesis and transpiration. Maximum carboxylation rate by Rubisco (V_{cmax}, μmol $m^{-2}s^{-1}$), regeneration of ribulose-1,5-bisphosphate represented by electron transport rate (J_{max}, μmol $m^{-2}s^{-1}$), rate constant for g_s decrease to steady-state (k_d, min), rate constant for g_s increase to maximum (k_i, min), and Ball-Berry slope and intercept. Values represent mean ± SE. n=8 for V_{cmax} and J_{max}, and n= 3-4 for other parameters. Different letters represent statistically significant differences (P<0.05) among the cultivars. For V_{cmax} and J_{max}, the values represent the mean used in the model. Standard error and statistics for these two parameters are shown in Table 1.

Cultivar	V_{cmax}	J_{max}	k_d	k_i	Ball-Berry slope	Ball-Berry intercept
Mbundumali	100.1	169.4	7.4 ± 1.59abc	10.8 ± 2.42b	5.50 ± 0.61a	0.079 ± 0.019a
TME3	101.8	165.4	7.2 ± 1.89bc	9.7 ± 1.63b	6.36 ± 0.94a	0.087 ± 0.016a
TME419	107.8	167.6	5.0 ± 0.83ab	9.4 ± 3.07ab	5.32 ± 0.73a	0.078 ± 0.013a
TME693	110.3	171.3	6.5 ± 1.03c	10.6 ± 2.02b	6.50 ± 0.22a	0.072 ± 0.033a
TME7	104.8	163.4	6.8 ± 1.73ab	9.1 ± 0.99b	5.80 ± 0.77a	0.070 ± 0.009a
TMS01/1412	106.3	175.9	4.8 ± 1.06ab	6.6 ± 1.79a	6.24 ± 0.48a	0.072 ± 0.014a
TMS30001	117.2	169.7	16.1 ± 1.46ab	12.5 ± 0.54b	6.43 ± 0.49a	0.047 ± 0.005a
TMS30572	95.2	154.5	16.2 ± 2.46c	11.5 ± 1.21b	6.63 ± 1.03a	0.069 ± 0.017a
TMS96/1632	102.7	163.2	5.8 ± 1.28ab	8.8 ± 0.42b	5.48 ± 0.80a	0.110 ± 0.033a
TMS98/0002	91.7	149.1	9.9 ± 0.66a	10.8 ± 0.82b	5.00 ± 0.49a	0.096 ± 0.015a
TMS98/0581	99.3	148.7	6.4 ± 2.2a	8.8 ± 1.50ab	7.29 ± 0.87a	0.068 ± 0.012a

Table S2. Dataset from responses of leaf CO_2 uptake rate to photosynthetic photon flux density (A/PPFD curves) for each replicate of cassava cultivars. This data was used for the calculations of Ball-Berry parameters showed in Table S3. PARi = light inside the chamber; Photo = leaf CO_2 uptake rate; Cond = stomatal conductance; RH_S = relative humidity inside the chamber during the measurement; CO2S = CO_2 concentration inside the chamber during the measurement.

Cultivar	Rep#	PARi	Photo	Cond	RH_S	CO2S
Mbundumali	1	1798.49	18.2368	0.15826	58.1273	390.406
Mbundumali	1	1498.02	17.8552	0.15625	53.5131	390.592
Mbundumali	1	1201.06	16.9277	0.14695	48.9499	390.878
Mbundumali	1	1000.57	15.6103	0.12739	47.6702	391.7
Mbundumali	1	799.055	14.0945	0.11092	46.1386	392.579
Mbundumali	1	602.233	12.1108	0.09871	44.83	393.589
Mbundumali	1	398.599	9.88234	0.08476	42.8504	394.625
Mbundumali	1	200.678	5.76019	0.06573	40.946	396.796
Mbundumali	1	148.187	3.9815	0.05325	40.5488	397.79
Mbundumali	1	100.584	2.74285	0.04479	40.3451	398.518
Mbundumali	1	73.4568	1.45875	0.03868	40.4724	399.104
Mbundumali	1	49.9163	0.52088	0.03455	40.4352	399.528
Mbundumali	1	25.7114	-0.4212	0.03138	40.4239	400.064
Mbundumali	1	-0.0817	-2.3749	0.02971	40.4894	401.255
Mbundumali	2	1801.83	26.0069	0.29963	61.4982	385.938
Mbundumali	2	1499.6	27.0658	0.35414	59.1778	385.33
Mbundumali	2	1199.45	26.6006	0.36185	55.8175	385.775
Mbundumali	2	998.13	25.4273	0.32882	52.9913	386.254
Mbundumali	2	801.856	23.5958	0.2869	51.7608	387.342
Mbundumali	2	598.793	20.8977	0.25553	50.3627	388.813
Mbundumali	2	398.952	17.075	0.22229	48.2683	390.743
Mbundumali	2	200.402	10.0627	0.18869	46.7215	394.229
Mbundumali	2	151.771	7.76693	0.15571	46.1562	395.653
Mbundumali	2	99.6793	4.81875	0.12362	46.496	397.164
Mbundumali	2	75.5804	3.25856	0.09642	46.4029	397.983
Mbundumali	2	51.1945	2.15513	0.07462	47.01	398.605
Mbundumali	2	26.1276	0.25155	0.06016	47.4174	399.775
Mbundumali	2	-0.3755	-1.9329	0.05136	46.6218	400.762
Mbundumali	3	1799.76	20.7133	0.20193	70.3032	388.876
Mbundumali	3	1499.79	19.8174	0.22868	58.4993	389.24
Mbundumali	3	1197.77	20.0289	0.23638	53.5993	389.309
Mbundumali	3	1001.72	19.5088	0.24467	51.413	389.54
Mbundumali	3	800.041	18.3106	0.24656	50.1726	389.953

Mbundumali	3	598.513	16.1613	0.24309	46.7457	390.982
Mbundumali	3	399.173	12.9897	0.23349	45.0553	392.784
Mbundumali	3	200.991	6.66471	0.20498	43.4705	395.994
Mbundumali	3	148.397	4.73897	0.17533	43.7059	396.985
Mbundumali	3	100.741	2.38995	0.15221	43.244	398.189
Mbundumali	3	73.543	1.57258	0.1342	43.8254	398.689
Mbundumali	3	50.0216	0.28199	0.12081	43.5225	399.5
Mbundumali	3	25.8323	-0.7251	0.10996	43.5897	399.929
Mbundumali	3	-0.0654	-2.5134	0.10049	43.4009	400.832
Mbundumali	4	1802.15	21.5226	0.1984	52.2812	388.391
Mbundumali	4	1500.74	21.2271	0.20296	49.973	388.691
Mbundumali	4	1199.88	20.495	0.20252	46.1536	388.852
Mbundumali	4	998.518	19.5063	0.19649	43.9968	389.507
Mbundumali	4	799.967	17.9211	0.1877	42.1959	390.156
Mbundumali	4	598.186	15.2092	0.17204	40.0945	391.696
Mbundumali	4	398.978	11.8977	0.15571	39.1356	393.45
Mbundumali	4	200.618	6.68413	0.13777	37.4435	396.016
Mbundumali	4	151.991	4.76714	0.12212	36.9429	397.053
Mbundumali	4	99.7556	2.75838	0.10973	36.5871	398.172
Mbundumali	4	75.8663	1.72974	0.09999	36.2834	398.622
Mbundumali	4	51.5676	0.77929	0.09145	36.007	399.294
Mbundumali	4	24.3265	-1.2305	0.08428	35.7083	400.201
Mbundumali	4	1.40119	-2.7587	0.07649	35.4276	401.117
TME3	1	1802.15	17.6665	0.14617	66.2643	390.608
TME3	1	1500.74	16.4575	0.13653	63.3863	391.121
TME3	1	1199.88	15.8446	0.13411	56.9418	391.52
TME3	1	998.518	15.7088	0.1344	53.5758	391.674
TME3	1	799.967	15.2794	0.13442	50.1243	391.892
TME3	1	598.186	14.36	0.1296	47.7245	392.409
TME3	1	398.978	11.6556	0.11312	44.8677	393.546
TME3	1	200.618	6.88432	0.08329	43.3769	396.147
TME3	1	151.991	4.59401	0.06451	43.352	397.515
TME3	1	99.7556	3.35242	0.05511	43.0096	398.023
TME3	1	75.8663	2.14952	0.05022	43.5167	398.61
TME3	1	51.5676	1.45385	0.04759	43.1472	398.968
TME3	1	24.3265	0.09196	0.04631	42.7094	399.75
TME3	1	1.40119	-1.7503	0.04312	42.2376	400.792
TME3	2	1801.83	19.8064	0.1576	50.4211	389.413
TME3	2	1499.6	27.3976	0.27432	50.2515	385.387
TME3	2	1199.45	31.3504	0.38569	45.6887	383.146
TME3	2	998.13	29.3724	0.32905	43.246	384.106
TME3	2	801.856	25.9543	0.26943	42.0819	386.09
TME3	2	598.793	23.1462	0.24416	41.4537	387.569

TME3	2	398.952	18.4483	0.24668	39.5521	389.723
TME3	2	200.402	9.38349	0.18209	38.6662	394.525
TME3	2	151.771	6.91213	0.13228	38.5612	396.016
TME3	2	99.6793	4.08606	0.11209	38.1687	397.57
TME3	2	75.5804	2.34991	0.10008	37.8804	398.313
TME3	2	51.1945	0.88491	0.08993	37.6501	399.007
TME3	2	26.1276	-1.1319	0.08316	37.4891	400.243
TME3	2	-0.3755	-3.2324	0.07315	37.2705	401.3
TME3	3	1799.76	24.0083	0.23071	54.4992	387.176
TME3	3	1499.79	25.5285	0.26626	52.9832	386.325
TME3	3	1197.77	25.6302	0.28507	48.9179	386.328
TME3	3	1001.72	24.8892	0.28653	46.8938	386.507
TME3	3	800.041	23.4273	0.26959	45.5862	387.415
TME3	3	598.513	20.3857	0.23921	43.994	388.823
TME3	3	399.173	15.3764	0.20308	42.8375	391.754
TME3	3	200.991	8.20263	0.16641	41.2927	395.335
TME3	3	148.397	5.84619	0.13085	40.9731	396.628
TME3	3	100.741	3.38332	0.10985	40.5085	397.912
TME3	3	73.543	1.71863	0.09293	40.071	398.682
TME3	3	50.0216	0.08159	0.07929	39.9643	399.664
TME3	3	25.8323	-1.5095	0.07058	39.9709	400.562
TME3	3	-0.0654	-3.4168	0.06293	39.7563	401.467
TME3	4	1799.76	29.3568	0.44674	53.0845	383.673
TME3	4	1499.79	28.5867	0.44514	52.5922	384.316
TME3	4	1197.77	27.8186	0.43295	52.0685	384.645
TME3	4	1001.72	26.657	0.4094	51.5305	385.534
TME3	4	800.041	25.1429	0.39217	51.0552	386.426
TME3	4	598.513	22.5156	0.37602	50.5986	387.804
TME3	4	399.173	18.7994	0.3522	50.077	389.693
TME3	4	200.991	11.0352	0.31647	49.3974	393.771
TME3	4	148.397	8.46613	0.27394	48.7646	395.022
TME3	4	100.741	5.27728	0.2327	48.1046	396.557
TME3	4	73.543	3.58213	0.18689	47.4031	397.677
TME3	4	50.0216	2.08007	0.14153	46.6237	398.699
TME3	4	25.8323	0.46683	0.10312	45.9126	399.244
TME3	4	-0.0654	-1.4632	0.0729	45.2846	400.553
TME419	1	1802.15	21.8644	0.23331	63.9407	388.156
TME419	1	1500.74	21.3541	0.2361	58.451	388.613
TME419	1	1199.88	20.5334	0.2185	52.5461	389.049
TME419	1	998.518	18.7362	0.19337	49.649	389.919
TME419	1	799.967	16.2379	0.16349	47.2403	391.029
TME419	1	598.186	13.9946	0.14354	45.3246	392.436
TME419	1	398.978	10.6888	0.12301	43.1505	394.067

TME419	1	200.618	5.9996	0.10377	40.8765	396.441
TME419	1	151.991	4.29309	0.08553	40.2321	397.617
TME419	1	99.7556	2.17698	0.07254	39.8433	398.707
TME419	1	75.8663	1.11429	0.06293	39.5585	399.159
TME419	1	51.5676	0.17503	0.05701	39.3902	399.608
TME419	1	24.3265	-1.2931	0.05172	39.2436	400.465
TME419	1	1.40119	-3.2772	0.0476	39.1567	401.369
TME419	2	1798.49	22.9737	0.2462	60.2865	387.472
TME419	2	1498.02	22.1906	0.23225	55.4608	388.127
TME419	2	1201.06	21.5931	0.22714	50.0383	388.437
TME419	2	1000.57	21.821	0.23396	47.7505	388.311
TME419	2	799.055	20.3895	0.21702	45.9465	389.029
TME419	2	602.233	17.5649	0.18914	43.7138	390.469
TME419	2	398.599	13.0788	0.15822	42.3894	392.763
TME419	2	200.678	6.66483	0.12674	40.2755	396.079
TME419	2	148.187	4.71133	0.10487	42.5088	397.324
TME419	2	100.584	2.55717	0.09507	40.0704	398.334
TME419	2	73.4568	0.69732	0.08463	40.6816	399.245
TME419	2	49.9163	0.26611	0.08248	40.0135	399.747
TME419	2	25.7114	-1.0972	0.07709	39.3751	400.332
TME419	2	-0.0817	-2.7604	0.07566	39.5086	401.233
TME419	3	1801.83	25.89	0.32441	55.3645	386.145
TME419	3	1499.6	25.1691	0.31458	54.8205	386.544
TME419	3	1199.45	24.2515	0.30308	54.2835	386.85
TME419	3	998.13	23.3184	0.28904	53.882	387.621
TME419	3	801.856	21.9559	0.27155	53.5307	388.33
TME419	3	598.793	19.7764	0.25135	53.0842	389.333
TME419	3	398.952	16.2759	0.22446	52.6719	391.117
TME419	3	200.402	9.99445	0.18612	51.9853	394.495
TME419	3	151.771	7.54429	0.14115	51.1979	395.736
TME419	3	99.6793	5.21124	0.11013	50.5396	397.069
TME419	3	75.5804	3.86938	0.08633	50.1044	397.733
TME419	3	51.1945	2.78258	0.07265	49.8944	398.202
TME419	3	26.1276	1.63863	0.06342	49.7184	398.913
TME419	3	-0.3755	-0.7706	0.05537	49.4667	400.105
TME419	4	1802.15	20.9721	0.22403	60.7803	388.699
TME419	4	1500.74	20.6249	0.22421	60.5018	388.985
TME419	4	1199.88	20.0443	0.22544	60.2329	389.39
TME419	4	998.518	19.3369	0.22448	60.0578	389.604
TME419	4	799.967	18.5365	0.22119	59.8296	390.092
TME419	4	598.186	17.5258	0.21601	59.5878	390.704
TME419	4	398.978	14.9182	0.20885	59.3119	392.068
TME419	4	200.618	9.37815	0.19817	59.0258	394.893

TME419	4	151.991	7.34569	0.18059	58.7373	396.022
TME419	4	99.7556	5.00668	0.16547	58.4878	397.202
TME419	4	75.8663	3.88241	0.14638	58.1996	397.661
TME419	4	51.5676	2.77157	0.12732	57.9591	398.186
TME419	4	24.3265	0.32	0.11223	57.714	399.507
TME419	4	1.40119	-1.0771	0.09851	57.4591	400.363
TME693	1	1798.49	16.0914	0.13425	63.639	391.611
TME693	1	1498.02	16.9402	0.15821	60.3018	390.95
TME693	1	1201.06	17.3139	0.18267	52.0541	390.595
TME693	1	1000.57	17.1698	0.17178	49.302	390.84
TME693	1	799.055	15.7475	0.14859	47.6532	391.589
TME693	1	602.233	13.806	0.1272	44.3922	392.668
TME693	1	398.599	11.8022	0.11504	42.7227	393.685
TME693	1	200.678	6.87811	0.08758	40.8035	396.135
TME693	1	148.187	5.06603	0.06527	40.8652	397.104
TME693	1	100.584	3.36533	0.05726	40.6455	397.87
TME693	1	73.4568	2.3145	0.05066	40.4318	398.585
TME693	1	49.9163	1.14657	0.04229	40.1872	399.16
TME693	1	25.7114	0.19567	0.03603	40.0421	399.675
TME693	1	-0.0817	-1.8067	0.03311	39.9904	400.85
TME693	2	1798.49	29.8267	0.43178	54.997	383.743
TME693	2	1498.02	28.8094	0.42341	54.3937	384.462
TME693	2	1201.06	27.3429	0.4118	53.7887	385.051
TME693	2	1000.57	26.1412	0.39816	53.309	385.762
TME693	2	799.055	24.315	0.38399	52.8529	386.536
TME693	2	602.233	22.2075	0.36566	52.4226	387.842
TME693	2	398.599	18.1909	0.34802	52.0151	390.073
TME693	2	200.678	11.0639	0.3274	51.5845	393.638
TME693	2	148.187	8.47092	0.30422	51.2418	394.992
TME693	2	100.584	5.75618	0.275	50.7794	396.37
TME693	2	73.4568	3.69734	0.24098	50.2479	397.489
TME693	2	49.9163	2.68287	0.20226	49.6838	398.139
TME693	2	25.7114	1.04841	0.16133	49.1224	399.113
TME693	2	-0.0817	-0.8276	0.123	48.541	399.86
TME693	3	1799.34	23.0581	0.22936	52.6457	387.56
TME693	3	1499.84	22.8439	0.23385	52.4041	387.68
TME693	3	1201.43	22.0103	0.2329	52.1104	388.128
TME693	3	1001.41	21.1029	0.22368	51.7845	388.821
TME693	3	799.433	19.7728	0.21226	51.3952	389.377
TME693	3	598.051	17.7211	0.19585	50.4256	390.615
TME693	3	398.133	14.6	0.17313	50.1078	392.033
TME693	3	200.909	9.10689	0.14406	49.7926	394.967
TME693	3	149.803	6.77686	0.12053	49.203	396.249

TME693	3	98.3667	4.42937	0.09634	48.7235	397.487
TME693	3	76.5514	3.19065	0.07245	48.1784	398.176
TME693	3	49.6985	1.89498	0.05345	47.8996	398.88
TME693	3	25.9889	0.44306	0.04076	47.1152	399.74
TME693	3	-0.1852	-0.9994	0.03246	47.5958	400.348
TME693	4	1801.83	21.2795	0.17153	59.5305	388.73
TME693	4	1499.6	21.5298	0.18564	54.2972	388.634
TME693	4	1199.45	23.5558	0.24821	47.5795	387.392
TME693	4	998.13	22.6444	0.24554	43.9474	387.791
TME693	4	801.856	19.5681	0.18274	44.018	389.567
TME693	4	598.793	15.0756	0.13412	43.1625	392.107
TME693	4	398.952	9.15345	0.08413	41.7242	394.909
TME693	4	200.402	4.70847	0.0593	40.7621	397.466
TME693	4	151.771	3.54757	0.05082	40.4106	398.032
TME693	4	99.6793	2.10505	0.04724	39.8569	398.865
TME693	4	75.5804	1.38468	0.04454	39.3896	399.068
TME693	4	51.1945	0.47366	0.04207	38.9744	399.732
TME693	4	26.1276	-0.8031	0.04053	38.5869	400.339
TME7	1	1798.49	16.895	0.1597	57.744	390.909
TME7	1	1498.02	14.2062	0.11762	54.9751	392.327
TME7	1	1201.06	14.0171	0.12432	49.9908	392.539
TME7	1	1000.57	15.6437	0.15742	47.7411	391.637
TME7	1	799.055	15.7366	0.17159	45.3006	391.497
TME7	1	602.233	14.6962	0.16722	43.552	391.878
TME7	1	398.599	12.1314	0.15015	41.9672	393.559
TME7	1	200.678	6.32055	0.10543	40.9619	396.336
TME7	1	148.187	4.4125	0.07461	40.4457	397.486
TME7	1	100.584	2.62362	0.06249	40.7249	398.464
TME7	1	73.4568	1.90957	0.05516	41.0096	398.817
TME7	1	49.9163	0.7442	0.0508	40.9381	399.418
TME7	1	25.7114	-0.1753	0.04758	40.8994	399.849
TME7	1	-0.0817	-1.7811	0.04442	40.9153	400.747
TME7	2	1801.83	20.359	0.14743	61.6067	389.209
TME7	2	1499.6	21.9341	0.18837	58.6491	388.314
TME7	2	1199.45	22.1967	0.19928	52.2962	388.116
TME7	2	998.13	21.3419	0.19133	48.8945	388.516
TME7	2	801.856	19.3844	0.17622	46.7162	389.619
TME7	2	598.793	16.6216	0.15059	44.1975	391.094
TME7	2	398.952	12.0479	0.11957	42.3846	393.49
TME7	2	200.402	6.08824	0.09234	40.4604	396.524
TME7	2	151.771	4.90658	0.08131	41.4812	397.263
TME7	2	99.6793	3.02172	0.07798	40.4305	398.115
TME7	2	75.5804	2.15738	0.07641	39.5968	398.426

TME7	2	51.1945	0.3699	0.07197	41.3563	399.529
TME7	2	26.1276	-0.1773	0.07362	39.4404	399.847
TME7	2	-0.3755	-3.1097	0.06882	39.6003	401.294
TME7	3	1799.76	20.4373	0.21747	57.6609	388.947
TME7	3	1499.79	19.7848	0.2096	54.532	389.486
TME7	3	1197.77	19.0694	0.21074	49.6684	389.78
TME7	3	1001.72	18.7178	0.21089	47.3261	389.83
TME7	3	800.041	18.5362	0.21295	45.1632	390.019
TME7	3	598.513	16.5241	0.20903	43.5859	391.076
TME7	3	399.173	12.9884	0.19767	41.5591	392.706
TME7	3	200.991	7.01353	0.16892	40.1109	395.868
TME7	3	148.397	5.06552	0.13846	40.2124	396.915
TME7	3	100.741	2.7263	0.11821	40.1133	398.164
TME7	3	73.543	1.59278	0.10091	40.4484	398.775
TME7	3	50.0216	0.16919	0.09008	39.8848	399.548
TME7	3	25.8323	-1.0391	0.081	39.2623	400.187
TME7	3	-0.0654	-2.826	0.0718	39.0251	400.968
TME7	4	1802.15	22.6006	0.24383	48.7967	387.734
TME7	4	1500.74	24.0017	0.30159	44.3594	387.034
TME7	4	1199.88	22.3255	0.27453	40.9149	387.814
TME7	4	998.518	20.9752	0.23432	40.7499	388.637
TME7	4	799.967	19.2728	0.21783	39.5732	389.675
TME7	4	598.186	16.8417	0.1971	37.5268	390.889
TME7	4	398.978	12.965	0.15952	36.1637	392.957
TME7	4	200.618	6.72002	0.11816	34.9443	396.062
TME7	4	151.991	4.66623	0.08722	35.2891	397.34
TME7	4	99.7556	2.76856	0.07377	34.8239	398.288
TME7	4	75.8663	1.52949	0.06743	34.6773	398.934
TME7	4	51.5676	0.49034	0.06481	34.6251	399.452
TME7	4	24.3265	-0.4864	0.06402	34.1599	400.009
TME7	4	1.40119	-2.4075	0.06254	34.0945	400.853
TMS01/1412	1	1802.15	19.011	0.17484	50.9701	389.856
TMS01/1412	1	1500.74	17.4978	0.15651	46.6319	390.634
TMS01/1412	1	1199.88	16.0572	0.13729	42.7295	391.321
TMS01/1412	1	998.518	14.2349	0.11857	40.6736	392.366
TMS01/1412	1	799.967	13.2153	0.10952	39.5358	392.873
TMS01/1412	1	598.186	12.1159	0.10426	37.5717	393.552
TMS01/1412	1	398.978	9.9469	0.09537	35.8516	394.502
TMS01/1412	1	200.618	6.15347	0.08351	34.116	396.404
TMS01/1412	1	151.991	4.57109	0.0692	33.7275	397.387
TMS01/1412	1	99.7556	2.8576	0.06238	33.5432	398.292
TMS01/1412	1	75.8663	2.06144	0.05739	33.8206	398.88
TMS01/1412	1	51.5676	1.16607	0.05484	33.6667	399.147

TMS01/1412	1	24.3265	-0.4895	0.05267	33.5325	400.043
TMS01/1412	1	1.40119	-2.0393	0.05039	33.385	400.922
TMS01/1412	2	1798.49	21.851	0.25224	53.2303	388.212
TMS01/1412	2	1498.02	20.9746	0.21958	50.414	388.814
TMS01/1412	2	1201.06	20.0048	0.21068	47.7102	389.093
TMS01/1412	2	1000.57	19.1915	0.20132	45.4456	389.665
TMS01/1412	2	799.055	18.0677	0.18845	43.7003	390.295
TMS01/1412	2	602.233	15.9621	0.16867	42.2281	391.538
TMS01/1412	2	398.599	12.0068	0.13635	40.9046	393.402
TMS01/1412	2	200.678	6.59194	0.10013	39.9332	396.106
TMS01/1412	2	148.187	4.6893	0.07359	40.1751	397.382
TMS01/1412	2	100.584	2.46353	0.05806	39.5547	398.499
TMS01/1412	2	73.4568	1.65949	0.05	39.3678	398.999
TMS01/1412	2	49.9163	0.59432	0.04664	39.2871	399.525
TMS01/1412	2	25.7114	-0.4713	0.04504	39.2457	399.989
TMS01/1412	2	-0.0817	-2.0572	0.04322	39.2035	400.919
TMS01/1412	3	1799.76	22.6091	0.20761	61.3997	387.793
TMS01/1412	3	1499.79	23.6352	0.23562	56.129	387.404
TMS01/1412	3	1197.77	23.0104	0.25624	49.1196	387.434
TMS01/1412	3	1001.72	22.8091	0.2634	45.4291	387.647
TMS01/1412	3	800.041	21.0906	0.26362	43.3092	388.504
TMS01/1412	3	598.513	18.2658	0.25242	40.9179	389.783
TMS01/1412	3	399.173	13.601	0.2307	38.7969	392.27
TMS01/1412	3	200.991	6.97877	0.19588	36.9458	395.715
TMS01/1412	3	148.397	4.88487	0.15354	37.2526	396.978
TMS01/1412	3	100.741	2.55771	0.12304	37.6288	398.294
TMS01/1412	3	73.543	1.48224	0.10212	37.2626	398.627
TMS01/1412	3	50.0216	0.30797	0.08904	36.9247	399.51
TMS01/1412	3	25.8323	-1.1527	0.08081	36.6361	400.153
TMS01/1412	3	-0.0654	-2.9746	0.07438	36.3974	401.116
TMS01/1412	4	1801.83	20.0621	0.16978	65.0925	389.304
TMS01/1412	4	1499.6	19.7677	0.16398	61.755	389.655
TMS01/1412	4	1199.45	18.689	0.17004	55.1525	389.866
TMS01/1412	4	998.13	19.0932	0.17761	51.2214	389.848
TMS01/1412	4	801.856	18.2723	0.17804	48.5121	390.132
TMS01/1412	4	598.793	16.551	0.17246	45.7122	390.995
TMS01/1412	4	398.952	12.9235	0.15653	43.4415	392.991
TMS01/1412	4	200.402	7.37301	0.13804	41.3214	395.734
TMS01/1412	4	151.771	5.82406	0.12321	40.7175	396.646
TMS01/1412	4	99.6793	3.34426	0.11383	40.3362	397.831
TMS01/1412	4	75.5804	2.22436	0.10682	40.0671	398.407
TMS01/1412	4	51.1945	1.2977	0.1027	39.8847	398.887
TMS01/1412	4	26.1276	-0.7215	0.1	39.3742	399.945

TMS01/1412	4	-0.3755	-2.1132	0.09768	38.8136	400.659
TMS30001	2	1801.83	21.1833	0.20849	49.6035	388.571
TMS30001	2	1499.6	19.9269	0.19119	48.3374	388.915
TMS30001	2	1199.45	18.6896	0.17391	44.6683	389.839
TMS30001	2	998.13	17.9462	0.16276	42.2806	390.438
TMS30001	2	801.856	16.4073	0.15155	40.6843	391.154
TMS30001	2	598.793	14.4812	0.13791	38.5399	392.055
TMS30001	2	398.952	11.472	0.11714	37.0338	393.774
TMS30001	2	200.402	6.40577	0.08859	35.7039	396.489
TMS30001	2	151.771	4.79348	0.07327	35.2212	397.057
TMS30001	2	99.6793	3.0964	0.06493	35.0341	397.994
TMS30001	2	75.5804	2.42784	0.0584	34.918	398.512
TMS30001	2	51.1945	1.49377	0.05466	34.4869	399.033
TMS30001	2	26.1276	-0.4457	0.05222	34.0419	400.029
TMS30001	2	-0.3755	-2.0169	0.05119	33.874	400.666
TMS30001	3	1799.76	21.9891	0.19925	54.4758	388.282
TMS30001	3	1499.79	21.4675	0.19643	51.3075	388.629
TMS30001	3	1197.77	20.3502	0.1878	47.2995	389.154
TMS30001	3	1001.72	18.7055	0.17266	45.0448	389.936
TMS30001	3	800.041	17.6636	0.15844	44.0257	390.631
TMS30001	3	598.513	15.7043	0.14582	42.6733	391.466
TMS30001	3	399.173	12.7318	0.12563	40.6901	393.108
TMS30001	3	200.991	7.41266	0.10385	39.3745	395.745
TMS30001	3	148.397	5.47471	0.08062	39.437	396.945
TMS30001	3	100.741	3.23554	0.06656	39.3321	398.071
TMS30001	3	73.543	1.89256	0.05563	38.9775	398.852
TMS30001	3	50.0216	0.51201	0.04774	38.6974	399.468
TMS30001	3	25.8323	-0.9767	0.04232	38.4829	400.34
TMS30001	3	-0.0654	-2.8047	0.03731	38.3151	401.157
TMS30001	4	1802.15	25.3218	0.24775	58.3818	386.622
TMS30001	4	1500.74	24.6166	0.23652	53.7817	386.9
TMS30001	4	1199.88	23.1356	0.22414	48.8937	387.653
TMS30001	4	998.518	22.3891	0.22088	45.4337	388.056
TMS30001	4	799.967	20.4853	0.19366	44.4141	389.086
TMS30001	4	598.186	17.7917	0.17506	42.0621	390.517
TMS30001	4	398.978	14.5467	0.16415	39.6722	392.084
TMS30001	4	200.618	7.77972	0.12814	37.6656	395.64
TMS30001	4	151.991	4.59204	0.09001	40.6992	397.28
TMS30001	4	99.7556	3.14172	0.07525	38.3174	398.204
TMS30001	4	75.8663	2.1005	0.05977	37.1812	398.809
TMS30001	4	51.5676	0.63617	0.05202	37.7523	399.49
TMS30001	4	24.3265	-0.8352	0.04524	38.2623	400.311
TMS30001	4	1.40119	-2.1024	0.0417	37.5044	400.912

TMS3001	1	1798.49	23.2621	0.21131	58.1171	387.627
TMS3001	1	1498.02	22.8134	0.21547	52.9574	387.884
TMS3001	1	1201.06	22.9625	0.2457	46.9911	387.623
TMS3001	1	1000.57	21.8801	0.23649	45.0948	388.302
TMS3001	1	799.055	20.4867	0.21122	44.0193	388.896
TMS3001	1	602.233	17.9544	0.17122	43.6538	390.508
TMS3001	1	398.599	13.6029	0.13612	41.8472	392.663
TMS3001	1	200.678	7.07561	0.08942	40.389	396.15
TMS3001	1	148.187	4.87993	0.06326	40.3714	397.312
TMS3001	1	100.584	3.33051	0.05025	40.1196	398.156
TMS3001	1	73.4568	2.15187	0.04271	39.9868	398.742
TMS3001	1	49.9163	1.10444	0.03898	39.9229	399.243
TMS3001	1	25.7114	0.2347	0.03677	39.8582	399.743
TMS3001	1	-0.0817	-1.7361	0.03601	39.9149	400.674
TMS30572	1	1798.49	16.3902	0.14087	59.6832	391.348
TMS30572	1	1498.02	17.2137	0.15008	57.6795	390.867
TMS30572	1	1201.06	17.5513	0.16182	52.3355	390.675
TMS30572	1	1000.57	17.2949	0.16483	50.043	390.748
TMS30572	1	799.055	16.7854	0.1664	47.593	390.939
TMS30572	1	602.233	15.9088	0.16462	45.1649	391.529
TMS30572	1	398.599	13.6652	0.16015	42.9039	392.709
TMS30572	1	200.678	7.45073	0.14852	40.4233	395.676
TMS30572	1	148.187	5.46528	0.12209	42.4092	396.738
TMS30572	1	100.584	3.39256	0.10715	40.6369	397.734
TMS30572	1	73.4568	2.02186	0.09395	39.9345	398.545
TMS30572	1	49.9163	0.59595	0.08532	39.7483	399.282
TMS30572	1	25.7114	-0.3195	0.07836	41.5511	399.877
TMS30572	1	-0.0817	-2.4237	0.07541	40.1174	400.85
TMS30572	2	1801.1	21.3814	0.27776	54.2697	388.2
TMS30572	2	1499.3	21.1595	0.27989	53.8129	388.363
TMS30572	2	1199.33	20.372	0.28095	53.3645	388.821
TMS30572	2	999.634	19.5685	0.27437	52.9769	389.264
TMS30572	2	799.914	18.6156	0.26457	52.6137	389.728
TMS30572	2	598.121	17.1503	0.2485	52.3375	390.616
TMS30572	2	397.706	14.4931	0.21759	51.7766	392.069
TMS30572	2	197.846	8.95147	0.17354	51.1302	395.072
TMS30572	2	152.088	6.82517	0.12683	50.3133	396.266
TMS30572	2	98.8494	4.2225	0.09779	49.6606	397.593
TMS30572	2	76.2805	3.34849	0.07871	49.189	398.106
TMS30572	2	51.7481	2.05864	0.06519	48.847	398.667
TMS30572	2	26.0188	0.64646	0.05742	48.6251	399.507
TMS30572	2	-0.2702	-0.7506	0.0514	48.4232	400.199
TMS30572	3	1800.45	22.2637	0.28668	56.9761	387.929

TMS30572	3	1498.43	21.4138	0.28362	56.509	388.385
TMS30572	3	1198.24	20.4031	0.27885	55.983	388.996
TMS30572	3	999.042	19.5078	0.27174	55.5642	389.368
TMS30572	3	799.907	18.2062	0.25398	55.048	390.199
TMS30572	3	602.271	16.2348	0.22884	54.4577	391.128
TMS30572	3	401.943	13.3203	0.20128	54.2995	392.715
TMS30572	3	201.235	8.25252	0.17371	53.7883	395.283
TMS30572	3	149.225	6.42253	0.13314	53.0025	396.307
TMS30572	3	100.055	4.26257	0.08799	52.0219	397.486
TMS30572	3	76.8007	3.23376	0.06148	51.2925	398.103
TMS30572	3	48.8466	2.02038	0.04426	50.8532	398.744
TMS30572	3	22.9388	1.03709	0.03403	50.5141	399.31
TMS30572	3	-0.5419	-0.6945	0.02806	50.3048	400.213
TMS30572	4	1799.76	24.7323	0.25133	58.3168	386.68
TMS30572	4	1499.79	23.8481	0.24052	55.1568	387.186
TMS30572	4	1197.77	23.1775	0.23947	50.2754	387.568
TMS30572	4	1001.72	23.1322	0.24356	47.1547	387.554
TMS30572	4	800.041	22.043	0.24859	44.5358	388.193
TMS30572	4	598.513	20.2111	0.24824	41.6553	388.987
TMS30572	4	399.173	15.7872	0.23118	40.0641	391.151
TMS30572	4	200.991	8.34088	0.19978	39.2137	394.87
TMS30572	4	148.397	6.1499	0.16206	39.2194	396.159
TMS30572	4	100.741	3.39876	0.13856	39.0528	397.669
TMS30572	4	73.543	2.20307	0.11673	38.7263	398.436
TMS30572	4	50.0216	1.16828	0.10208	38.5973	398.824
TMS30572	4	25.8323	-0.6096	0.09251	38.3183	399.842
TMS30572	4	-0.0654	-2.3378	0.08533	38.1393	400.858
TMS96/1632	1	1802.15	23.4877	0.22722	56.3111	387.425
TMS96/1632	1	1500.74	22.5404	0.20307	55.2047	388.04
TMS96/1632	1	1199.88	20.8892	0.18307	50.7179	388.956
TMS96/1632	1	998.518	19.9809	0.16776	48.3787	389.305
TMS96/1632	1	799.967	17.9453	0.1512	47.1899	390.129
TMS96/1632	1	598.186	15.9503	0.13704	45.7093	391.489
TMS96/1632	1	398.978	12.7977	0.12456	43.2062	393.329
TMS96/1632	1	200.618	7.37942	0.10341	41.6083	395.656
TMS96/1632	1	151.991	5.62927	0.0852	41.5746	396.693
TMS96/1632	1	99.7556	3.52575	0.07205	41.4612	397.901
TMS96/1632	1	75.8663	2.05944	0.06077	41.2156	398.756
TMS96/1632	1	51.5676	0.84362	0.05156	41.215	399.215
TMS96/1632	1	24.3265	-0.894	0.04502	40.7188	400.45
TMS96/1632	1	1.40119	-3.286	0.03848	41.2564	401.398
TMS96/1632	2	1798.49	25.0088	0.27453	58.4897	386.463
TMS96/1632	2	1498.02	25.6393	0.29264	53.5273	386.199

TMS96/1632	2	1201.06	25.0951	0.29054	48.1013	386.408
TMS96/1632	2	1000.57	23.8487	0.2711	45.7361	387.105
TMS96/1632	2	799.055	21.6927	0.25384	43.8611	388.224
TMS96/1632	2	602.233	18.6633	0.22721	41.3547	389.857
TMS96/1632	2	398.599	14.1101	0.19495	39.845	392.375
TMS96/1632	2	200.678	7.29121	0.15539	38.0624	395.625
TMS96/1632	2	148.187	5.17942	0.12106	38.4577	397.071
TMS96/1632	2	100.584	2.97808	0.10193	38.378	398.103
TMS96/1632	2	73.4568	2.01298	0.09079	38.1589	398.497
TMS96/1632	2	49.9163	0.7196	0.08506	37.6631	399.327
TMS96/1632	2	25.7114	-1.1354	0.08251	37.5845	399.99
TMS96/1632	2	-0.0817	-2.5517	0.08151	37.5343	401.009
TMS96/1632	3	1799.76	36.1287	0.42379	58.8969	380.677
TMS96/1632	3	1499.79	35.1607	0.45785	53.835	381.169
TMS96/1632	3	1197.77	33.7813	0.48062	49.1632	381.792
TMS96/1632	3	1001.72	32.2596	0.48318	47.8533	382.321
TMS96/1632	3	800.041	29.4319	0.48112	46.3267	383.86
TMS96/1632	3	598.513	25.0861	0.46066	43.5436	386.056
TMS96/1632	3	399.173	18.1855	0.43257	41.4468	389.454
TMS96/1632	3	200.991	9.20802	0.38607	40.328	394.122
TMS96/1632	3	148.397	6.22407	0.32648	40.239	395.722
TMS96/1632	3	100.741	3.76247	0.27425	40.5226	397.204
TMS96/1632	3	73.543	2.18913	0.21982	40.9489	398.151
TMS96/1632	3	50.0216	0.60072	0.17569	41.2309	399.031
TMS96/1632	3	25.8323	-1.0058	0.14929	40.7916	399.933
TMS96/1632	3	-0.0654	-2.9553	0.12942	40.3816	401.115
TMS96/1632	4	1801.83	21.6144	0.20342	56.5523	388.448
TMS96/1632	4	1499.6	20.99	0.19935	52.896	388.789
TMS96/1632	4	1199.45	20.4092	0.19559	48.509	389.022
TMS96/1632	4	998.13	19.5252	0.18287	46.6637	389.594
TMS96/1632	4	801.856	17.8622	0.17187	44.3123	390.424
TMS96/1632	4	598.793	16.2976	0.16179	42.3824	391.225
TMS96/1632	4	398.952	12.6448	0.15083	40.9573	393.111
TMS96/1632	4	200.402	6.6183	0.13715	38.6485	396.103
TMS96/1632	4	151.771	4.89514	0.12505	37.9281	397.134
TMS96/1632	4	99.6793	2.71557	0.11341	40.2448	398.264
TMS96/1632	4	75.5804	1.53544	0.10565	38.3912	398.75
TMS96/1632	4	51.1945	0.04364	0.10008	37.4396	399.493
TMS96/1632	4	26.1276	-1.0159	0.0939	37.191	400.163
TMS96/1632	4	-0.3755	-2.7007	0.08793	37.0359	400.935
TMS98/0002	1	1802.15	18.5616	0.17338	63.1168	390.044
TMS98/0002	1	1500.74	18.4201	0.18245	57.6285	390.045
TMS98/0002	1	1199.88	18.2803	0.1829	51.6046	390.147

TMS98/0002	1	998.518	17.6185	0.17835	48.9217	390.399
TMS98/0002	1	799.967	16.7548	0.17546	46.2739	390.961
TMS98/0002	1	598.186	15.7051	0.17296	43.7408	391.48
TMS98/0002	1	398.978	13.2629	0.16381	41.5424	392.829
TMS98/0002	1	200.618	7.25006	0.14245	39.1137	395.799
TMS98/0002	1	151.991	5.58909	0.1226	39.3342	396.715
TMS98/0002	1	99.7556	3.28895	0.10901	39.2159	397.932
TMS98/0002	1	75.8663	2.15076	0.09668	38.6123	398.412
TMS98/0002	1	51.5676	1.02873	0.08903	38.7662	399.141
TMS98/0002	1	24.3265	-0.7748	0.08607	38.7718	399.978
TMS98/0002	1	1.40119	-2.6721	0.08265	38.3554	401.071
TMS98/0002	2	1801.83	19.664	0.19033	54.002	389.284
TMS98/0002	2	1499.6	18.9164	0.18202	50.2702	389.729
TMS98/0002	2	1199.45	18.1316	0.17245	45.9771	390.406
TMS98/0002	2	998.13	17.0691	0.16302	44.6137	390.88
TMS98/0002	2	801.856	16.0532	0.15428	43.3102	391.381
TMS98/0002	2	598.793	14.015	0.14396	41.2837	392.286
TMS98/0002	2	398.952	11.127	0.12594	39.9118	393.811
TMS98/0002	2	200.402	6.6188	0.11066	39.0709	396.202
TMS98/0002	2	151.771	5.00402	0.09477	38.6691	397.129
TMS98/0002	2	99.6793	2.65088	0.08473	38.4152	398.294
TMS98/0002	2	75.5804	1.57659	0.07814	38.1831	398.966
TMS98/0002	2	51.1945	0.31349	0.07373	38.0482	399.457
TMS98/0002	2	26.1276	-1.1768	0.07044	37.9669	400.217
TMS98/0002	2	-0.3755	-3.3229	0.06811	37.9342	401.281
TMS98/0002	3	1799.76	24.6642	0.30105	58.1767	386.792
TMS98/0002	3	1499.79	25.143	0.31063	55.0282	386.442
TMS98/0002	3	1197.77	23.9596	0.28242	52.4	387.13
TMS98/0002	3	1001.72	22.6885	0.26029	49.5217	387.883
TMS98/0002	3	800.041	20.951	0.24661	47.1889	388.731
TMS98/0002	3	598.513	18.4722	0.22101	44.9611	389.979
TMS98/0002	3	399.173	13.9367	0.18381	44.3324	392.338
TMS98/0002	3	200.991	7.19944	0.13977	42.7375	395.913
TMS98/0002	3	148.397	4.84008	0.11075	42.9502	397.258
TMS98/0002	3	100.741	2.90519	0.09216	42.8368	398.113
TMS98/0002	3	73.543	1.88083	0.08095	42.2479	398.663
TMS98/0002	3	50.0216	0.38052	0.07411	41.98	399.572
TMS98/0002	3	25.8323	-0.6109	0.06894	41.4421	400.138
TMS98/0002	3	-0.0654	-2.8921	0.06363	40.8418	401.271
TMS98/0002	4	1801.08	20.0613	0.2797	61.8972	388.996
TMS98/0002	4	1499.64	19.783	0.29321	61.7447	389.214
TMS98/0002	4	1198.05	19.064	0.29304	61.3619	389.679
TMS98/0002	4	998.038	18.4073	0.28502	61.201	390.04

TMS98/0002	4	800.181	17.5815	0.28031	60.8891	390.479
TMS98/0002	4	598.602	16.4564	0.27389	60.3773	391.134
TMS98/0002	4	398.638	14.3421	0.26489	60.1599	392.187
TMS98/0002	4	201.214	8.92541	0.25102	59.7685	394.957
TMS98/0002	4	150.003	6.69341	0.23022	59.6144	396.062
TMS98/0002	4	98.4235	4.12486	0.20494	59.3736	397.535
TMS98/0002	4	76.6201	2.95038	0.17728	58.872	398.141
TMS98/0002	4	49.843	1.3648	0.15325	58.3831	398.956
TMS98/0002	4	26.087	0.0765	0.13336	58.1895	399.59
TMS98/0002	4	-0.2327	-1.7678	0.1195	57.8443	400.567
TMS98/0581	1	1798.49	17.4492	0.20951	56.6373	390.551
TMS98/0581	1	1498.02	17.3042	0.19792	55.8052	390.666
TMS98/0581	1	1201.06	16.0921	0.18354	50.3602	391.18
TMS98/0581	1	1000.57	15.078	0.16639	47.5809	391.828
TMS98/0581	1	799.055	13.782	0.15379	45.888	392.498
TMS98/0581	1	602.233	12.0836	0.14246	43.9318	393.332
TMS98/0581	1	398.599	9.71623	0.1293	41.8203	394.586
TMS98/0581	1	200.678	5.57358	0.11607	39.975	396.621
TMS98/0581	1	148.187	4.2322	0.10461	39.45	397.29
TMS98/0581	1	100.584	2.49198	0.09653	39.069	398.237
TMS98/0581	1	73.4568	1.72675	0.08974	38.7996	398.637
TMS98/0581	1	49.9163	0.97293	0.08491	38.5944	399.245
TMS98/0581	1	25.7114	-1.0034	0.08135	38.4397	400.22
TMS98/0581	1	-0.0817	-2.1878	0.07707	38.304	400.854
TMS98/0581	2	1500.74	24.4496	0.21013	51.5126	386.944
TMS98/0581	2	1199.88	24.7038	0.26383	46.726	386.742
TMS98/0581	2	998.518	25.7014	0.2951	43.0304	386.187
TMS98/0581	2	799.967	24.9808	0.3113	41.5966	386.543
TMS98/0581	2	598.186	22.571	0.31485	39.719	387.603
TMS98/0581	2	398.978	17.1985	0.28242	38.5106	390.406
TMS98/0581	2	200.618	8.95505	0.19291	38.1388	394.658
TMS98/0581	2	151.991	6.33009	0.11575	39.0976	396.299
TMS98/0581	2	99.7556	3.82062	0.0908	38.9514	397.658
TMS98/0581	2	75.8663	2.52021	0.08078	37.9249	398.425
TMS98/0581	2	51.5676	1.03093	0.07636	37.7351	399.287
TMS98/0581	2	24.3265	-0.6281	0.0765	37.4491	399.993
TMS98/0581	2	1.40119	-2.5149	0.07674	37.1383	400.905
TMS98/0581	3	1799.76	19.3188	0.22458	59.9177	389.672
TMS98/0581	3	1499.79	19.5027	0.25862	55.4955	389.354
TMS98/0581	3	1197.77	19.7119	0.27998	49.8635	389.331
TMS98/0581	3	1001.72	18.9202	0.28543	46.9284	389.574
TMS98/0581	3	800.041	17.9476	0.27779	45.3001	390.182
TMS98/0581	3	598.513	15.961	0.26177	43.352	391.237

TMS98/0581	3	399.173	12.4439	0.22953	41.1135	392.887
TMS98/0581	3	200.991	6.56865	0.17511	40.7077	395.975
TMS98/0581	3	148.397	4.93035	0.12145	42.3545	396.959
TMS98/0581	3	100.741	2.76418	0.09393	42.0524	398.117
TMS98/0581	3	73.543	1.78792	0.07977	41.701	398.62
TMS98/0581	3	50.0216	0.52403	0.07315	41.44	399.395
TMS98/0581	3	25.8323	-0.5268	0.06822	41.208	400.131
TMS98/0581	3	-0.0654	-2.0986	0.0647	41.0181	400.777
TMS98/0581	4	1798.66	14.7737	0.15105	60.2939	392.115
TMS98/0581	4	1500.78	15.3042	0.17436	60.3956	391.865
TMS98/0581	4	1199.58	16.995	0.2114	60.7605	390.84
TMS98/0581	4	1000.16	17.974	0.23026	60.8847	390.527
TMS98/0581	4	797.917	17.87	0.23845	60.806	390.595
TMS98/0581	4	602.16	17.0904	0.24954	60.6933	390.867
TMS98/0581	4	401.378	14.464	0.25237	60.494	392.265
TMS98/0581	4	200.233	8.64288	0.24373	60.2288	395.251
TMS98/0581	4	148.108	6.54073	0.18975	59.641	396.291
TMS98/0581	4	98.952	4.19833	0.11617	58.7415	397.619
TMS98/0581	4	75.5975	2.58765	0.05241	57.8244	398.493
TMS98/0581	4	51.6872	1.35162	0.03175	57.4369	399.256
TMS98/0581	4	25.6072	0.83372	0.02551	57.4341	399.598
TMS98/0581	4	-0.5237	-0.8347	0.02273	57.6689	400.309

Table S3. Matrix with values (r) obtained from Pearson's correlation and its p-values (P) between variables measured under steady-state in cassava. Values in bold represent statistically significant correlation (P<0.05). In green, values that were highlighted in results and discussion of the main text.

		A_{sat}	$V_{cmax,ci}$	$J_{max,ci}$	V_{TPU}	g_s	g_m	$V_{cmax,\,cc}$	$J_{max,cc}$	*iWUE*	operating c_i	Chla	Chlb	Chl a/b ratio	Chl total	TSP	TSP as Rubisco	TSP/Chl	Rubisco initial activity	Rubisco total activity	Rubisco activation state	Rubisco content	Rubisco specific activity
$V_{cmax,ci}$	r	**0.222**																					
	P	**0.033**																					
$J_{max,ci}$	r	0.075	**0.819**																				
	P	0.470	**<0.0001**																				
V_{TPU}	r	0.069	**0.783**	**0.944**																			
	P	0.506	**<0.0001**	**<0.0001**																			
g_s	r	0.075	0.082	0.096	0.019																		
	P	0.471	0.436	0.355	0.855																		
g_m	r	**0.268**	**0.332**	0.174	0.213	0.144																	
	P	**0.042**	**0.013**	0.195	0.111	0.280																	
$V_{cmax,\,cc}$	r	-0.033	0.121	0.214	**0.244**	0.101	0.080																
	P	0.763	0.284	0.052	**0.026**	0.359	0.057																
$J_{max,cc}$	r	-0.041	0.197	**0.287**	0.268	0.150	0.026	**0.880**															
	P	0.711	0.080	**0.009**	0.149	0.175	0.854	**<0.0001**															
iWUE	r	-0.052	0.195	**0.269**	**0.329**	**-0.789**	0.053	-0.044	-0.091														
	P	0.612	0.060	**0.009**	**0.001**	**<0.0001**	0.691	0.689	0.412														
operating c_i	r	0.044	**-0.221**	**-0.295**	**-0.352**	**0.758**	-0.072	0.019	0.057	**-0.995**													
	P	0.670	**0.034**	**0.004**	**0.001**	**<0.0001**	0.594	0.862	0.608	**<0.0001**													
Chla	r	0.032	0.088	0.272	0.163	-0.174	-0.316	-0.085	0.163	0.077	-0.080												
	P	0.823	0.534	0.051	0.250	0.213	0.142	0.596	0.315	0.585	0.575												
Chlb	r	-0.053	0.057	0.251	0.149	-0.267	-0.370	-0.091	0.176	0.128	-0.125	**0.965**											
	P	0.707	0.688	0.072	0.291	0.055	0.082	0.572	0.278	0.366	0.376	**<0.0001**											
Chl a/b ratio	r	**0.302**	0.101	-0.012	-0.005	**0.445**	0.361	0.092	-0.083	-0.251	0.235	-0.260	**-0.502**										
	P	**0.029**	0.476	0.931	0.971	**0.001**	0.090	0.568	0.609	0.072	0.093	0.063	**0.000**										
Chl total	r	0.005	0.079	0.268	0.160	-0.205	-0.334	-0.088	0.168	0.094	-0.095	**0.997**	**0.983**	**-0.338**									
	P	0.970	0.577	0.055	0.258	0.145	0.119	0.586	0.300	0.508	0.505	**<0.0001**	**<0.0001**	**0.014**									
TSP	r	0.073	0.248	**0.368**	**0.351**	0.083	0.007	0.158	0.215	-0.025	0.022	0.220	0.161	0.115	0.204								
	P	0.624	0.094	**0.011**	**0.016**	0.577	0.974	0.358	0.216	0.865	0.881	0.136	0.281	0.442	0.170								
TSP as Rubisco	r	0.160	-0.189	-0.277	-0.283	0.108	0.095	-0.085	-0.211	-0.080	0.069	-0.103	-0.114	0.075	-0.107	**-0.774**							
	P	0.289	0.207	0.062	0.057	0.476	0.683	0.623	0.224	0.598	0.647	0.497	0.450	0.618	0.478	**<0.0001**							
TSP/Chl	r	0.079	0.227	0.279	**0.294**	0.142	0.131	0.184	0.142	-0.048	0.045	-0.139	-0.194	0.238	-0.158	**0.930**	**-0.766**						
	P	0.598	0.126	0.058	**0.045**	0.339	0.570	0.282	0.414	0.747	0.466	0.352	0.191	0.108	0.290	**<0.0001**	**<0.0001**						
Rubisco initial activity	r	**0.365**	0.267	0.273	0.229	0.263	0.324	0.260	0.179	-0.120	0.098	0.280	0.168	**0.307**	0.247	**0.517**	-0.058	**0.454**					
	P	**0.012**	0.070	0.632	0.122	0.075	0.163	0.120	0.298	0.422	0.511	0.057	0.259	**0.036**	0.094	**0.000**	0.714	**0.002**					
Rubisco total activity	r	**0.363**	0.276	0.263	0.186	0.254	0.169	0.274	0.212	-0.154	0.135	**0.370**	0.251	**0.293**	**0.336**	**0.468**	0.043	**0.372**	**0.936**				
	P	**0.011**	0.058	0.071	0.205	0.081	0.464	0.096	0.208	0.296	0.359	**0.010**	0.085	**0.043**	**0.020**	**0.001**	0.780	**0.013**	**<0.0001**				
Rubisco activation state	r	0.102	0.254	0.247	0.211	0.208	0.281	0.249	0.151	-0.120	0.113	0.073	0.025	0.170	0.059	**0.483**	-0.265	**0.467**	**0.753**	**0.484**			
	P	0.494	0.085	0.094	0.154	0.161	0.230	0.138	0.378	0.423	0.448	0.626	0.868	0.252	0.696	**0.001**	0.086	**0.002**	**<0.0001**	**0.001**			
Rubisco content	r	**0.460**	**0.374**	**0.417**	**0.353**	**0.330**	0.039	0.277	0.312	-0.174	0.147	**0.416**	**0.297**	0.258	**0.382**	**0.616**	-0.029	**0.483**	**0.850**	**0.911**	**0.446**		
	P	**0.001**	**0.009**	**0.003**	**0.014**	**0.022**	0.862	0.093	0.060	0.236	0.318	**0.003**	**0.040**	0.076	**0.007**	**<0.0001**	0.848	**0.001**	**<0.0001**	**<0.0001**	**0.002**		
Rubisco specific activity	r	-0.083	-0.005	-0.055	-0.112	0.125	-0.010	0.168	0.077	-0.173	0.181	0.012	-0.031	0.187	-0.001	-0.063	0.114	-0.052	**0.476**	**0.522**	**0.316**	0.130	
	P	0.578	0.973	0.715	0.455	0.402	0.966	0.320	0.656	0.246	0.224	0.936	0.839	0.207	0.994	0.683	0.461	0.738	**0.001**	**0.000**	**0.032**	0.382	
Rca total	r	-0.055	-0.145	-0.049	-0.045	0.079	-0.124	0.040	-0.037	-0.072	0.079	**-0.339**	-0.273	-0.053	**-0.321**	**-0.343**	0.218	-0.237	-0.236	-0.176	-0.171	**-0.315**	0.177
	P	0.712	0.330	0.743	0.766	0.600	0.592	0.816	0.833	0.630	0.596	**0.020**	0.063	0.723	**0.028**	**0.020**	0.151	0.112	0.132	0.258	0.279	**0.037**	0.257

Table S4. Chlorophyll a (Chl a, mg m^{-2}), chlorophyll b (Chl b, mg m^{-2}) and chlorophyll total (Chl total, mg m^{-2}) contents, chlorophyll a/b ratio, total soluble protein content (TSP, g m^{-2}), fraction of total soluble protein present as Rubisco (TSP as Rubisco, w/w), and ratio of total soluble protein to chlorophyll content (TSP/Chl) of cassava cultivars. Values represent mean ± SE. *n*=3-4. Different letters represent statistically significant differences ($P<0.05$) among the cultivars. TSP as Rubisco did not present statistically significant differences.

Cutivar	Chl a	Chl b	Chl a/b ratio	Chl total	TSP	TSP as Rubisco	TSP/Chl
**Mbundumali**	263.2 ± 19.43ab	112.42 ± 8.11ab	2.34 ± 0.02c	375.63 ± 27.49bc	3.79 ± 0.18ab	0.34 ± 0.04a	10.31 ± 1.12ab
**TME3**	252.79 ± 10.92ab	99.34 ± 4.57abc	2.55 ± 0.02ab	352.13 ± 15.46abc	4.9 ± 0.85ab	0.4 ± 0.07a	14.5 ± 2.39ab
**TME419**	257.7 ± 9.65ab	107.85 ± 5.52ab	2.39 ± 0.03ac	365.55 ± 15.16abc	4.95 ± 0.43ab	0.34 ± 0.02a	13.93 ± 1.69ab
**TME693**	239.34 ± 6.33ab	92.28 ± 1.85abc	2.59 ± 0.06b	331.63 ± 7.39abc	3.55 ± 0.56ab	0.52 ± 0.07a	10.71 ± 1.7ab
**TME7**	220.79 ± 11.58ab	89.44 ± 5.59bc	2.47 ± 0.03abc	310.23 ± 17.17ab	5.71 ± 0.74a	0.32 ± 0.03a	17.42 ± 2.08a
**TMS01/1412**	280.13 ± 2.57a	115.75 ± 0.36a	2.42 ± 0.02ac	395.88 ± 2.69a	3.3 ± 0.24b	0.46 ± 0.02a	8.36 ± 0.54b
**TMS30001**	260.21 ± 4.96ab	106.67 ± 3.09ab	2.44 ± 0.03abc	366.88 ± 8.01abc	4.58 ± 0.36ab	0.39 ± 0.01a	12.49 ± 0.9ab
**TMS30572**	254.31 ± 15.39ab	104.11 ± 5.96abc	2.44 ± 0.01abc	358.42 ± 21.34abc	3.07 ± 0.12b	0.46 ± 0.05a	8.63 ± 0.47b
**TMS96/1632**	267.84 ± 7.97a	110.07 ± 3.34ab	2.43 ± 0.01ac	377.9 ± 11.31bc	4.57 ± 0.64ab	0.43 ± 0.06a	12.01 ± 1.42ab
**TMS97/2205**	249.72 ± 23.07ab	102 ± 8.21abc	2.44 ± 0.03abc	351.72 ± 31.28abc	3.15 ± 0.66ab	0.42 ± 0.1a	9.2 ± 1.25ab
**TMS98/0002**	251.15 ± 13.88ab	104.17 ± 4.3abc	2.41 ± 0.03ac	355.32 ± 18.17abc	4.22 ± 0.76ab	0.43 ± 0.09a	11.74 ± 1.79ab
**TMS98/0505**	236.48 ± 3.89ab	93.77 ± 1.71abc	2.52 ± 0.03ab	330.25 ± 5.38abc	4.39 ± 0.54ab	0.37 ± 0.03a	13.22 ± 1.45ab
**TMS98/0581**	205.49 ± 5.83b	80.87 ± 3.36c	2.55 ± 0.04ab	286.35 ± 9.19c	3.06 ± 0.46ab	0.49 ± 0.06a	10.85 ± 1.99ab

Table S5. The effect of a three-fold acceleration of stomata response on carbon assimilation rate (*A*, μmol $m^{-2}s^{-1}$) and water use efficiency (*WUE*, μmol CO_2 mol H_2O^{-1}) during light induction from deep shade (50 μmol m^{-2} s^{-1}), and during low (150 μmol m^{-2} s^{-1}) and high (1500 μmol m^{-2} s^{-1}) light periods in contrasting cassava cultivars. Numbers between brackets indicate the time interval (min) considered in the simulations. Results were normalized by measured stomata parameters.

Cultivars	Induction from deep shade				Low light				High light			
	**[0,10]**	**[10,20]**	**[20,30]**	**[30,40]**	**[40,50]**	**[50,60]**	**[60,70]**	**[70,80]**	**[80,90]**	**[90,100]**	**[100,110]**	**[110,120]**
	A											
**TME693 k_i*3**	1.11	1.03	1.01	1.00	1.00	1.00	1.00	1.00	1.03	1.01	1.00	1.00
**TME693 k_i*3 k_d*3**	1.11	1.03	1.01	1.00	0.99	0.99	0.99	0.99	1.03	1.01	1.00	1.00
**TMS01/1412 k_i*3**	1.07	1.01	1.00	1.00	1.00	1.00	1.00	1.00	1.03	1.00	1.00	1.00
**TMS01/1412 k_i*3 k_d*3**	1.07	1.01	1.00	1.00	0.99	1.00	1.00	1.00	1.03	1.00	1.00	1.00
	WUE											
	**Induction from deep shade**				**Low light**				**High light**			
	**[0,10]**	**[10,20]**	**[20,30]**	**[30,40]**	**[40,50]**	**[50,60]**	**[60,70]**	**[70,80]**	**[80,90]**	**[90,100]**	**[100,110]**	**[110,120]**
**TME693 k_i*3**	0.77	0.84	0.93	0.97	0.98	0.99	0.99	1.00	0.87	0.90	0.96	0.98
**TME693 k_i*3 k_d*3**	0.77	0.84	0.93	0.97	1.16	1.29	1.20	1.11	0.88	0.91	0.96	0.98
**TMS01/1412 k_i*3**	0.82	0.94	0.99	1.00	1.00	1.00	1.00	1.00	0.88	0.96	0.99	1.00
**TMS01/1412 k_i*3 k_d*3**	0.82	0.94	0.99	1.00	1.20	1.06	1.01	1.00	0.88	0.96	0.99	1.00

Notes S1. Detailed description of cassava photosynthesis model

Cassava photosynthesis model

To estimate the influence of stomata and Rubisco response on dynamic photosynthesis rate, a cassava photosynthesis metabolic model was developed. The model was constructed based on the C_3 photosynthesis model (Zhu et al. 2007), a simplified light reaction model, a Rubisco activase model (Mate et al. 1996; Zhu et al. 2013), and a dynamic stomatal conductance model (Vialet-Chabrand et al. 2017). The model was implemented in MATLAB. A brief description of each one the models used here follows this. Our prior model (Zhu et al. 2007) is a general C_3 photosynthesis model, which includes the reactions of the Calvin-Benson cycle and carbohydrate synthesis. In the model, the rate of change of the concentration of each metabolite over time is represented by ordinary differential equation (ODE):

$$\frac{dC}{dt} = V_p - V_u$$

where: C is metabolite concentration; V_p is the total rate of the reaction that produces C, and V_u is the total rate of C consumption. Rate equations of each reaction were developed based on standard Michaelis-Menten equations, except where the assumptions of this formulation were not met, notably in the case of Rubisco. The alternative equations to meet these situations followed Zhu et al. (2007).

The model was parameterized using V_{cmax}, J_{max}, k_i, k_d, Ball-Berry slope and intercept from measured photosynthetic and stomata parameters of cassava (Table A). The measured V_{cmax} was used as the maximum Rubisco activity in the metabolic model. A, transpiration (T), c_i, and g_s were estimated under various light conditions. The predicted water use efficiency (WUE) was calculated dividing A by T.

Table A. Input parameters of the cassava model

Cultivar	Vcmax25	Jmax25	K_d (s^{-1})	K_i (s^{-1})	Ball-Berry Slope	Ball-Berry Intercept
Mbundumali	100.1	169.4	0.0026	0.0017	5.50	0.079
TME3	101.8	165.4	0.0017	0.0019	6.36	0.087
TME419	107.8	167.6	0.0034	0.0022	5.32	0.078
TME693	110.3	171.3	0.0011	0.0017	6.50	0.072
TME7	104.8	163.4	0.0030	0.0020	5.80	0.070
TMS01/1412	106.3	175.9	0.0035	0.0031	6.24	0.072
TMS30001	117.2	169.7	0.0036	0.0018	6.43	0.047
TMS30572	95.2	154.5	0.0010	0.0015	6.63	0.069
TMS96/1632	102.7	163.2	0.0029	0.0019	5.48	0.110
TMS98/0002	91.7	149.1	0.0039	0.0016	5.00	0.096
TMS98/0581	99.3	148.7	0.0035	0.0021	7.29	0.068

C_3 photosynthesis metabolic model

Description

Based on the C_3 photosynthesis model (Zhu et al., 2007), the model parameters and equations used in cassava model followed as described below (Tables B, C and D). The subscripts of v1, v2 ……v131 correspond to the numbers in Zhu et al., (2007).

Table B. The Michaelis-Menten constants, inhibition constants and activation constants of the enzymes in the Calvin cycle, starch synthesis and triose phosphate export (Zhu et al. 2007).

RN	Reaction	Par	Value (mM)	Description
**1**	$RuBP+CO_2 \rightarrow 2PGA$	K_{M11}	0.0115	CO_2
**1**	$RuBP+CO_2 \rightarrow 2PGA$	K_{M12}	0.222	O_2
**1**	$RuBP+CO_2 \rightarrow 2PGA$	K_{M13}	0.020	RuBP

**1**	$RuBP+CO_2 \rightarrow 2PGA$	K_{I11}	0.84	PGA
**1**	$RuBP+CO_2 \rightarrow 2PGA$	K_{I12}	0.04	FBP
**1**	$RuBP+CO_2 \rightarrow 2PGA$	K_{I13}	0.075	SBP
**1**	$RuBP+CO_2 \rightarrow 2PGA$	K_{I14}	0.9	Pi
**1**	$RuBP+CO_2 \rightarrow 2PGA$	K_{I15}	0.07	NADPH
**2**	PGA+ATP ↔ADP + DPGA	K_{M21}	0.240	PGA
**2**	PGA+ATP ↔ADP + DPGA	K_{M22}	0.390	ATP
**2**	PGA+ATP ↔ADP + DPGA	K_{M23}	0.23	ADP
**2**	PGA+ATP↔ADP + DPGA	K_{E2}	7.6×10^{-4}	Equ. Const.
**3**	$DPGA+NADPH+H^+ \leftrightarrow GAP + Pi+NADP$	K_{M31}	0.004	BPGA
**3**	$DPGA+NADPH +H^+ \leftrightarrow GAP + Pi+NADP$	K_{M32}	0.100	NADPH
**4**	DHAP ↔GAP	K_{E4}	0.05	Equ. Const.
**5**	GAP+DHAP ↔FBP	K_{M51}	0.3	GAP
**5**	GAP+DHAP ↔FBP	K_{M52}	0.4	DHAP
**5**	GAP+DHAP ↔FBP	K_{M53}	0.02	FBP
**5**	GAP+DHAP ↔FBP	K_{E5}	7.1	Equ. Const.
**6**	FBP→F6P+Pi	K_{M61}	0.033	FBP
**6**	FBP→F6P+Pi	K_{I61}	0.7	F6P
**6**	FBP→F6P+Pi	K_{I62}	12	Pi
**6**	FBP→F6P+Pi	K_{E6}	6.7×10^5	Equ Const.
**7**	F6P+GAP→E4P+Xu5P	K_{M71}	0.1	Xu5P
**7**	F6P+GAP→E4P+Xu5P	K_{M72}	0.1	GAP
**7**	F6P+GAP→E4P+Xu5P	K_{E7}	10	Equ. Const.
**8**	E4P+DHAP→SBP	K_{M8}	0.02	SBP
**8**	E4P+DHAP→SBP	K_{M81}	0.4	DHAP
**8**	E4P+DHAP→SBP	K_{M82}	0.2	E4P
**8**	E4P+DHAP→SBP	K_{E8}	1.07	Equ. Const.
**9**	SBP→S7P+Pi	K_{M9}	0.05	SBP
**9**	SBP→S7P+Pi	K_{I9}	12	Pi
**9**	SBP→S7P+Pi	K_{E9}	6.7×10^5	Equ. Const.
**10**	S7P+GAP→Ri5P+Xu5P	K_{M101}	0.118	P5P
**10**	S7P+GAP→Ri5P+Xu5P	K_{M102}	0.072	GAP
**10**	S7P+GAP→Ri5P+Xu5P	K_{M103}	0.46	S7P
**10**	S7P+GAP→Ri5P+Xu5P	K_{M104}	1.54	F6P
**10**	S7P+GAP→Ri5P+Xu5P	K_{E10}	1.17	Equ. Const.
**11**	Ri5P↔Ru5P	K_{E11}	0.4	Equ. Const.
**12**	Xu5P↔Ru5P	K_{E12}	0.67	Equ. Const.

**13**	Ru5P+ATP→RuBP+ADP	K_{M131}	0.05	Ru5P
**13**	Ru5P+ATP→RuBP+ADP	K_{M132}	0.059	ATP
**13**	Ru5P+ATP→RuBP+ADP	K_{I131}	2	PGA
**13**	Ru5P+ATP→RuBP+ADP	K_{I132}	0.7	RuBP
**13**	Ru5P+ATP→RuBP+ADP	K_{I133}	4	Pi
**13**	Ru5P+ATP→RuBP+ADP	K_{I134}	2.5	ADP
**13**	Ru5P+ATP→RuBP+ADP	K_{E13}	0.4	ADP
**13**	Ru5P+ATP→RuBP+ADP	K_{I135}	6846	Equ. Const.
**16**	ADP+Pi→ATP	K_{M161}	0.014	ADP
**16**	ADP+Pi→ATP	K_{M162}	0.3	Pi
**16**	ADP+Pi→ATP	K_{M163}	0.3	ATP
**16**	ADP+Pi→ATP	K_{E16}	5.7	Equ. Const.
**21**	F6P↔G6P	K_{E21}	2.3	Equ. Const.
**22**	G6P↔G1P	K_{E22}	0.058	Equ. Const.
**23**	G1P+ATP+Gn→PPi+ADP+G_{n+1}	K_{M231}	0.08	G1P
**23**	G1P+ATP+Gn→PPi+ADP+G_{n+1}	K_{M232}	0.08	ATP
**23**	G1P+ATP+Gn→PPi+ADP+G_{n+1}	K_{A231}	0.1	PGA
**23**	G1P+ATP+Gn→PPi+ADP+G_{n+1}	K_{A232}	0.02	F6P
**23**	G1P+ATP+Gn→PPi+ADP+G_{n+1}	K_{A233}	0.02	FBP
**23**	G1P+ATP+Gn→PPi+ADP+G_{n+1}	K_{I23}	10	ADP
**31**	P_{ext} +$DHAP_i$→Pi+$DHAP_o$	K_{M311}	0.077	DHAP
**31**	P_{ext} +$DHAP_i$→Pi+$DHAP_o$	K_{M312}	0.63	Pi
**31**	P_{ext} +$DHAP_i$→Pi+$DHAP_o$	K_{M313}	0.74	P_{ext}
**32**	P_{ext} +PGA_i→Pi+PGA_o	K_{M32}	0.25	PGA
**33**	P_{ext} +GAP_i→Pi+GAP_o	K_{M33}	0.075	GAP
**112**	2-PGCA +H_2O → GCA + Pi	K_{M112}	0.026	PGCA
**112**	2-PGCA +H_2O → GCA + Pi	K_{I1121}	94	GCA, competitive with PGCA
**112**	2-PGCA +H_2O → GCA + Pi	K_{I1122}	2.55	Pi, competitive with PGCA
**113**	GCEA + ATP→ PGA + ADP	K_{M1131}	0.21	ATP
**113**	GCEA + ATP→ PGA + ADP	K_{M1132}	0.25	GCEA
**113**	GCEA + ATP→ PGA + ADP	K_{I113}	0.36	PGA, competitive with ATP

**113**	GCEA + ATP→ PGA + ADP	K_{E113}	300	Equil. Const.
**121**	$GCA_C+0_2 \rightarrow H_2O_2+GOAc$	K_{M121}	0.1	GCAc
**122**	GOAc + SERc→ HPRc + GLYc	K_{M1221}	0.15	GOAc
**122**	GOAc + SERc→ HPRc + GLYc	K_{M1222}	2.7	SERc
**122**	GOAc + SERc→ HPRc + GLYc	K_{I1221}	33	GLYc, competitive with SERc
**122**	GOAc + SERc→ HPRc + GLYc	K_{E122}	0.24	Equil. Const.
**123**	HPRc + NADc→ NADHc + GCEAc	K_{M123}	0.09	HPRc
**123**	HPRc + NADc→ NADHc + GCEAc	K_{I123}	12	HPRc, self inibition
**123**	HPRc + NADc→ NADHc + GCEAc	K_{E123}	2.5×10^5	Equil. Const.
**124**	GOAc + GLUc→ KGc + GLYc	K_{M1241}	0.15	GOAc
**124**	GOAc + GLUc→ KGc + GLYc	K_{M1242}	1.7	GLUc
**124**	GOAc + GLUc→ KGc + GLYc	K_{I124}	2	GLYc competitive with GLU
**124**	GOAc + GLUc→ KGc + GLYc	K_{E124}	607	Equi. Const.
**131**	GLYc + NADc→ CO2 + NH3 + SERc +NADHc	K_{M1311}	6	GLYc
**131**	GLYc + NADc→ CO2 + NH3 + SERc +NADHc	K_{I1311}	4	SERc, competitive with GLYc
**101a**	GCEAc → GCEA	K_{M1011}	0.39	GCEA
**101a**	GCEAc→GCEA	K_{I1011}	0.28	GCA, competitive with GCEA
**101b**	GCA→GCAc	K_{M1012}	0.2	GCA
**101b**	GCA→GCAc	K_{I1012}	0.22	GCEA, competitive with GCA
**51**	DHAPc + PGAc ↔ FBPc	K_{m511}	.020	FBPc
**51**	DHAPc + PGAc ↔ FBPc	K_{m512}	.300	GAPc
**51**	DHAPc + PGAc ↔ FBPc	K_{m513}	.400	DHAPc
**51**	DHAPc + PGAc ↔ FBPc	K_{m514}	.014	SBPc
**51**	DHAPc + PGAc ↔ FBPc	K_{E51}	12	
**52**	FBPc ↔ F6Pc + Pic	K_{m521}	.0025	FBPc
**52**	FBPc ↔ F6Pc + Pic	K_{I521}	.7	F6Pc

**52**	FBPc ↔ F6Pc + Pic	K_{I522}	12	Pic
**52**	FBPc ↔ F6Pc + Pic	K_{I523}	$7*10^{-5}$	F26BPc
**52**	FBPc ↔ F6Pc + Pic	K_{E52}	6663	
**55**	G1Pc + UTPc↔GDPc +UDPGc	K_{m551}	.14	G1Pc
**55**	G1Pc + UTPc↔GDPc +UDPGc	K_{m552}	.1	UTPc
**55**	G1Pc + UTPc↔GDPc +UDPGc	K_{m553}	.11	OPOPc
**55**	G1Pc + UTPc↔GDPc+UDPGc	K_{m554}	.12	UDPGc
**55**	G1Pc + UTPc↔ GDPc+UDPGc	K_{E55}	0.31	Equi
**56**	UDPGc + F6Pc ↔ SUCPc + UDPc	K_{m561}	0.8	F6Pc
**56**	UDPGc + F6Pc ↔ SUCPc + UDPc	K_{I562}	.8	FBPc
**56**	UDPGc + F6Pc ↔ SUCPc + UDPc	K_{I563}	0.4	SUCPc
**56**	UDPGc + F6Pc ↔ SUCPc + UDPc	K_{I564}	11	Pic
**56**	UDPGc + F6Pc ↔ SUCPc + UDPc	K_{I565}	50	Sucrose
**56**	UDPGc + F6Pc ↔ SUCPc + UDPc	K_{E56}	10	Equl. Const.
**57**	SUCPc ↔ Pic + SUCc	K_{m571}	.35	SUCPc
**57**	SUCPc ↔ Pic + SUCc	K_{i572}	80	SUCc
**57**	SUCPc ↔ Pic + SUCc	K_{E57}	780	Equil. Const.
**58**	F26BPc ↔ F6Pc + Pic	K_{m581}	.032	F26BPc
**58**	F26BPc ↔ F6Pc + Pic	K_{I581}	.1	F6Pc
**58**	F26BPc ↔ F6Pc + Pic	K_{I582}	.5	Pic
**59**	F6Pc + ATPc ↔ F26BPc + ADPc	K_{m591}	0.5	ATPc
**59**	F6Pc + ATPc ↔ F26BPc + ADPc	K_{m592}	.021	F26BPc
**59**	F6Pc + ATPc ↔ F26BPc + ADPc	K_{m593}	0.5	F6Pc
**59**	F6Pc + ATPc ↔ F26BPc + ADPc	K_{I591}	.16	ADPc
**59**	F6Pc + ATPc ↔ F26BPc + ADPc	K_{I592}	0.7	DHAPc
**59**	F6Pc + ATPc ↔ F26BPc + ADPc	K_{E59}	590	
**60**	F6Pc + ATPc ↔ F26BPc + ADPc	K_{m601}	0.042	ADPc
**60**	F6Pc + ATPc ↔ F26BPc + ADPc	K_{m602}	1.66	ATPc
**60**	F6Pc + ATPc ↔ F26BPc + ADPc	K_{m603}	0.28	UDPc
**60**	F6Pc + ATPc ↔ F26BPc + ADPc	K_{m604}	16	UTPc
**60**	F6Pc + ATPc ↔ F26BPc + ADPc	K_{E60}	16	Equili.
**61**	SUCPc ↔ SUCc + Pic	K_{E61}	$1.2*10^{7}$	Equili.
**62**	SUCc ↔ Sink	K_{m621}	5	Sucrose

Table C. The maximum rate of each enzyme (V_m) normalized on maximum Rubisco carboxylation activity (V_{cmax}).

Maximum Velocity	Enzyme	Reaction	V_m/V_1
V_1	Rubisco	$RuBP+CO_2 \rightarrow 2PGA$	1
V_2	PGA Kinase	$PGA+ATP \rightarrow ADP + DPGA$	10.3
V_3	GAP dehydragenase	$DPGA+NADPH \rightarrow GAP + Pi+NADP$	1.39
V_5	FBP Aldolase	$GAP+DHAP \rightarrow FBP$	0.42
V_6	FBPase	$FBP \rightarrow F6P+Pi$	0.25
V_7	Transketolase	$F6P+GAP \rightarrow E4P+Xu5P$	1.07
V_8	Aldolase	$E4P+DHAP \rightarrow SBP$	0.42
V_9	SBPase	$SBP \rightarrow S7P+Pi$	0.11
V_{10}	Transketolase	$S7P+GAP \rightarrow Ri5P+Xu5P$	1.07
V_{13}	Ribulosebiphosphate kinase	$Ru5P+ATP \rightarrow RuBP+ADP$	3.71
V_{16}	ATP synthase	$ADP+Pi \rightarrow ATP$	5.5
V_{23}	ADP-glucose pyrophosphorylase and Starch Synthase	$ADPG+G_n \rightarrow G_{(n+1)}+ADP$	0.1
V_{31}	Phosphate translocator	$DHAP_i \rightarrow DHAP_o$	0.3
V_{32}	Phosphate translocator	$PGA_i \rightarrow PGA_o$	0.3
V_{33}	Phosphate translocator	$GAP_i \rightarrow GAP_o$	0.3
V_{111}	Rubisco	$RuBP + O_2 \rightarrow PGA + PGCA$	0.24
V_{112}	Phosphoglycolate phosphatase	$2\text{-}PGCA + H_2O \rightarrow GCA + Pi$	18.0
V_{113}	Glycerate kinase	$GCEA + ATP \rightarrow PGA + ADP$	1.96
V_{121}	Glycolate oxidase	$GCA_C+0_2 \rightarrow H_2O_2+GOAc$	0.45
V_{122}	Serine glyoxylate aminotransferase	$GOAc + SERc \rightarrow HPRc + GLYc$	1.13
V_{123}	NADH-hydroxypyruvate reductase	$HPRc + NADc \rightarrow NADHc + GCEAc$	3.44
V_{124}	Glutamate glyoxylate aminotransferase (GGAT)	$GOAc + GLUc \rightarrow KGc + GLYc$	0.94
V_{131}	Glycine decarboxylase	$GLYc + NADc \rightarrow CO_2 + NH3 + SERc + NADHc$	0.86
V_{1T}	Glycerate/glycolate transporter	$GCEAc \leftrightarrow GCEA$	0.4
V_{2T}	Glycerate/glycolate transporter	$GCAc \leftrightarrow GCA$	0.4
V_{51}		$DHAPc + PGAc \leftrightarrow FBPc$	0.037
V_{52}		$FBPc \leftrightarrow F6Pc + Pic$	0.022

V_{55}	G1Pc + UTPc ↔ GDPc + UDPGc	0.040
V_{56}	UDPGc + F6Pc ↔ SUCPc + UDPc	0.019
V_{57}	SUCPc ↔ Pic + SUCc	0.19
V_{58}	F26BPc ↔ F6Pc + Pic	0.007
V_{59}	F6Pc + ATPc ↔ F26BPc + ADPc	0.002
V_{60}	ATPc + UDPc ↔ UTPc + ADPc	1

Table D. Initial concentrations of metabolites

Metabolite name	Localization	Model default (mmol l^{-1})
**RuBP**	Chl	2.000
**PGA**	Chl	2.400
**DPGA**	Chl	0.0011
**GAP**	Chl	0.02
**DHAP**	Chl	0.48
**FBP**	Chl	0.670
**E4P**	Chl	0.050
**S7P**	Chl	2.0
**SBP**	Chl	0.30
**ATP**	Chl	0.68
**NADPH**	Chl	0.21
**CO_2**	Chl	0.012
**O_2**	Chl	0.26
**HexP**	Chl	2.2
**PenP**	Chl	0.25
**Pi**	Chl	5
**CP**	Chl	15
**CA**	Chl	1.5
**CN**	Chl	0.5
**P_{ext}**	Chl	0.5
**NADH**	Chl	0.22
**NADHc**	Cyto	0.47
**NAD**	Chl	0.08
**NADc**	Cyt	0.02

**ATP**	Chl	0.68
**ATPc**	Cyt	0.36
**ADP**	Chl	0.82
**ADPc**	Cyt	0.64
**GLUc**	Cyt	24
**KGc**	Cyt	0.4
**Pic**	Chl	5
**SERc**	Cyt	7.5
**GLYc**	Cyt	1.8
**PGA**	Chl	4.3
**GOAc**	Cyt	0.028
**GCA**	Chl	0.36
**GCAc**	Cyt	0.36
**PGCA**	Chl	0.003
**HPRc**	Cyt	0.004
**GCEA**	Chl	0.18
**GCEAc**	Cyt	0.18
**TPc**	Cyt	2.3
**FBPc**	Cyt	2
**F26BPc**	Cyt	7×10^{-6}
**UTc**	Cyt	1
**HexPc**	Cyt	6
**UDPG**	Cyt	0.6
**PTc**	Cyt	15
**ATc**	Cyt	1

<u>Equations from the model</u>

1) The rate equations of the photosynthetic carbon metabolism (Zhu et al., 2007).

$$v_1 = \frac{RuBP \times Wc \times \min(1, \frac{RuBP}{E_t})}{(RuBP + K_r(1 + \frac{PGA}{K_{I11}} + \frac{FBP}{K_{I12}} + \frac{SBP}{K_{I13}} + \frac{Pi}{K_{I14}} + \frac{NADPH}{K_{I15}}))}$$

$$W_C = \frac{V_{Cmax} \times CO_2}{CO_2 + K_{M11}(1 + \frac{O_2}{K_{M12}})}$$

$$v_2 = \frac{V_2 \times (PGA \times ATP - \frac{DPGA \times ADP}{K_{E2}})}{(PGA + K_{M21})(ATP + K_{M22}(1 + \frac{ADP}{K_{M23}}))}$$

$$v_3 = \frac{V_3 \times DPGA \times NADPH}{(DPGA + K_{M31})(NADPH + K_{M32})}$$

$$v_5 = \frac{V_5 \times (GAP \times DHAP - \frac{FBP}{K_{E5}})}{K_{M51}K_{M52}(1 + \frac{GAP}{K_{M51}} + \frac{DHAP}{K_{M52}} + \frac{FBP}{K_{M53}} + \frac{GAP \times DHAP}{K_{M51}K_{M52}})}$$

$$v_6 = \frac{V_6 \times (FBP - \frac{F6P \times Pi}{K_{E6}})}{FBP + K_{M61}(1 + \frac{F6P}{K_{I61}} + \frac{Pi}{K_{I62}})}$$

$$TEMP1 = Xu5P \times (1 + \frac{E4P \times Ri5P}{Km71}) + E4P + Ri5P$$

$$Den = 1 + (1 + \frac{GAP}{K_{M72}}) \times (\frac{F6P}{K_{M104}} + \frac{S7P}{K_{M103}}) + \frac{GAP}{K_{M102}} + \frac{TEMP1}{K_{M101}}$$

$$v_7 = \frac{V_7 \times (F6P \times GAP \times K_{E7} - Xu5P \times E4P)}{(K_{M71} \times K_{M101} \times Den)}$$

$$v_8 = \frac{V_8 \times (DHAP \times E4P - \frac{SBP}{K_{E8}})}{(E4P + K_{M82})(DHAP + K_{M81})}$$

$$v_9 = \frac{V_9 \times (SBP - \frac{Pi \times S7P}{K_{E9}})}{SBP + K_{M9}(1 + \frac{Pi}{K_{I9}})}$$

$$v_{10} = \frac{V_{10} \times (GAP \times S7P \times K_{E10} - Ri5P \times Xu5P)}{(K_{M71} \times K_{M101} \times Den)}$$

$$v_{13} = \frac{V_{13} \times (ATP \times Ru5P - \frac{ADP \times RuBP}{K_{E13}})}{(ATP \times (1+\frac{ADP}{K_{I134}}) + K_{M132}(1+\frac{ADP}{K_{I135}}))(Ru5P + K_{M131}(1+\frac{GAP}{K_{I131}} + \frac{RuBP}{K_{I132}} + \frac{Pi}{K_{I133}}))}$$

$$v_{16} = \frac{V_{16} \times (ADP \times Pi - \frac{ATP}{K_{E13}})}{(ADP + K_{M161})(Pi + K_{M162})}$$

$$v_{23} = \frac{V_{23} \times G1P \times ATP}{(G1P + K_{M231})((1+\frac{ADP}{K_{I23}})(ATP + K_{M232}) + \frac{K_{M232} \times Pi}{K_{A231} \times PGA + K_{A232} \times F6P + K_{A233} \times FBP})}$$

$$N = 1 + (1+\frac{K_{M313}}{P_{ext}})(\frac{Pi}{K_{M312}} + \frac{PGA}{K_{M32}} + \frac{GAP}{K_{M33}} + \frac{DHAP}{K_{M311}})$$

$$v_{31} = \frac{V_{31} \times DHAP}{K_{M311}N}$$

$$v_{32} = \frac{V_{32} \times PGA}{K_{M32}N}$$

$$v_{33} = \frac{V_{33} \times GAP}{K_{M33}N}$$

$$v_{111} = \frac{RuBP \times W_o \times \min(1, \frac{RuBP}{E_t})}{(RuBP + K_r(1+\frac{PGA}{K_{I11}} + \frac{FBP}{K_{I12}} + \frac{SBP}{K_{I13}} + \frac{PI}{K_{I14}} + \frac{NADPH}{K_{I15}}))}$$

$$W_o = \frac{V_{111} \times O_2}{O_2 + K_O(1+\frac{CO_2}{k_C})}$$

$$v_{112} = \frac{V_{112} \times PGCA}{PGCA + K_{m112}(1 + GCA/K_{i1121})(1 + Pi/K_{I1122})}$$

$$v_{113} = \frac{V_{113} \times (ATP \times GCEA - \frac{ADP \times PGA}{K_{E113}})}{(ATP + Km_{1131}(1+\frac{PGA}{K_{i113}}))(GCEA + K_{m1132})}$$

$$v_{121} = \frac{V_{121} \times GCAc}{GCAc + K_{m121}}$$

$$v_{122} = \frac{V_{122} \times (GOAc \times SERc - \frac{HPRc \times GLYc}{K_{E122}})}{(GOAc + K_{m1221})(SERc + K_{m1222}(1+\frac{GLYc}{K_{i1221}}))}$$

$$v_{55} = \frac{V_{55} \times (UTPc \times G1Pc - \frac{UDPGc \times OPOPc}{K_{E551}})}{K_{m551}K_{m552}(1 + \frac{UTPc}{K_{m551}} + \frac{G1Pc}{K_{m552}} + \frac{UDPGc}{K_{m553}} + \frac{OPOPc}{K_{m554}} + \frac{UTPc \times G1Pc}{K_{m551}K_{m552}} + \frac{UDPGc \times OPOPc}{K_{m553}K_{m554}})}$$

$$v_{56} = \frac{V_{56} \times (F6Pc \times UDPGc - \frac{SUCPc \times UDPc}{K_{E56}})}{(F6Pc + K_{m561}(1 + \frac{FBPc}{K_{I562}}))(UDPGc + K_{m562}(1 + \frac{UDPc}{K_{I561}})(1 + \frac{SUCPc}{K_{I563}})(1 + \frac{SUCc}{K_{I565}})(1 + \frac{Pic}{K_{I564}}))}$$

$$v_{57} = \frac{V_{57} \times (SUCPc - \frac{SUCc \times Pic}{K_{E57}})}{SUCPc + K_{m571} \times (1 + \frac{SUCc}{K_{m572}})}$$

$$v_{58} = \frac{V_{58} \times F26BPc}{K_{m581} \times (1 + \frac{F26BPc}{K_{m581}})(1 + \frac{Pic}{K_{I582}})(1 + \frac{F6Pc}{K_{I581}})}$$

$$v_{59} = \frac{V_{59} \times (ATPc \times F6Pc - \frac{ADPc \times F26BPc}{K_{E59}})}{(F6Pc + K_{m593} \times (1 + \frac{F26BPc}{K_{m592}})(1 + \frac{DHAPc}{K_{I592}}))((ATPc + K_{m591} \times (1 + \frac{ADPc}{K_{I591}}))}$$

$$v60 = \frac{V_{60} \times (ATPc \times UDPc - ADPc \times UTPc/KE60)}{K_{m602}K_{m603} \times (1 + \frac{ATPc}{K_{m602}} + \frac{UDPc}{K_{m603}} + \frac{ATPc \times UDPc}{K_{m602} \times K_{m603}} + \frac{ADPc}{K_{m601}} + \frac{UTPc}{K_{m604}} + \frac{ADPc \times UTPc}{K_{m601}K_{m604}})}$$

$$v_{123} = \frac{V_{123} \times (HPRc \times NADHc - \frac{NADc \times Gcea}{K_{E123}})}{HPRc + K_{m1231}(1 + HPRc/K_{I123})}$$

$$v_{124} = \frac{V_{124} \times (GOAc \times GLUc - \frac{KGc \times GLYc}{K_{E124}})}{(GOAc + K_{m1241})(GLUc + K_{m1242}(1 + GLYc/K_{I124}))}$$

$$v_{131} = \frac{V_{131} \times GLYc}{GLYc + K_{m1311}(1 + SERc/K_{I1311})}$$

$$v_{1in} = V_{1T}\frac{GCEAc}{GCEAc + K_{M1011}(1+\frac{GCAc}{K_{I1011}})}$$

$$v_{1out} = V_{1T}\frac{GCEA}{GCEA + K_{M1011}(1+\frac{GCA}{K_{I1011}})}$$

$$v_{2out} = V_{2T}\frac{GCA}{GCA + K_{M1012}(1+\frac{GCEA}{K_{I1012}})}$$

$$v_{2in} = V_{2T}\frac{GCAc}{GCAc + K_{M1012}(1+\frac{GCEAc}{K_{I1012}})}$$

$$GAP = \frac{T3P}{1 + K_{e4}}$$

$$DHAP = \frac{K_{e3} \times T3P}{1 + K_{e4}}$$

$$Ru5P = \frac{Pent}{\frac{1}{K_{E11}} + \frac{1}{K_{E12}} + 1}$$

$$Ri5P = \frac{\frac{1}{K_{E11}} \times Pent}{\frac{1}{K_{E11}} + \frac{1}{K_{E12}} + 1}$$

$$Xu5P = \frac{\frac{1}{K_{E12}} \times Pent}{\frac{1}{K_{E11}} + \frac{1}{K_{E12}} + 1}$$

$$G6P = \frac{HexP}{\frac{1}{K_{E21}} + K_{E22} + 1}$$

$$F6P = \frac{\frac{1}{KE21} \times HexP}{\frac{1}{K_{E21}} + K_{E22} + 1}$$

$$G1P = \frac{K_{E22} \times HexP}{\frac{1}{K_{E21}} + K_{E22} + 1}$$

$[CA] = [ADP] + [ATP]$

$[CN] = [NADP] + [NADPH]$

$[CP] = [Pi] + [PGA] + 2[BPGA] + [GAP] + [DHAP] + 2[FBP] + [F6P] + [E4P] + 2[SBP]$

$+ [S7P] + [Xu5P] + [Ri5P] + [Ru5P] + 2[RuBP] + [G6P] + [G1P] + [ATP] + [PGCA]$

$[CA_c] = [ADP_c] + [ATP_c]$

$[CP_c] = 2 * ([FBP_c] + [F26BP_c]) + [PGA_c] + [T3P_c] + [HexP_c] + [SUCP] + [UTP_c] + [ATP_c] + [Pi]$

$[CN_c] = [NAD_c] + [NADH_c]$

$[CU_c] = [UDP_c] + [UTP_c]$

T3P = GAP + DHAP

Pent = Ru5P + Ri5P + Xu5P

HexP = G6P + F6P + G1P

2) Differential equations to describe rates of changes in each intermediate of carbon metabolism

$$\frac{d[RuBP]}{dt} = v_{13} - v_1 - v_{111};$$

$$\frac{d[PGA]}{dt} = 2v_1 - v_2 - v_{32} + v_{111} + v_{113};$$

$$\frac{d[DPGA]}{dt} = v_2 - v_3;$$

$$\frac{d[T3P]}{dt} = v_3 - 2\,v_5 - v_7 - v_8 - v_{10} - v_{31} - v_{33};$$

$$\frac{d[FBP]}{dt} = v_5 - v_6;$$

$$\frac{d[E4P]}{dt} = v_7 - v_8;$$

$$\frac{d[S7P]}{dt} = v_9 - v_{10};$$

$$\frac{d[SBP]}{dt} = v_8 - v_9;$$

$$\frac{d[ATP]}{dt} = v_{16} - v_2 - v_{23} - v_{13} - v_{113};$$

$$\frac{d[HexP]}{dt} = v_6 - v_7 - v_{23};$$

$$\frac{d[PenP]}{dt} = v_7 + 2v_{10} - v_{13};$$

$$\frac{d[GOAc]}{dt} = v_{121} - v_{122} - v_{124}$$

$$\frac{d[SER\,c]}{dt} = v_{131} - v_{122}$$

$$\frac{d[GLY\,c]}{dt} = v_{122} + v_{124} - 2\,v_{131}$$

$$\frac{d[HPRc]}{dt} = v_{122} - v_{123}$$

$$\frac{d[GCEAc]}{dt} = v_{123} - v_{1\,in} + v_{1\,out}$$

$$\frac{d[GCEA]}{dt} = v_{1in} - v_{113} - v_{1out}$$

$$\frac{d[GCA]}{dt} = v_{112} - v_{2out} + v_{2in}$$

$$\frac{d[PGCA]}{dt} = v_{111} - v_{112}$$

$$\frac{d[GCAc]}{dt} = v_{2out} - v_{121} - v_{2in}$$

$$\frac{d[OPOPc]}{dt} = v_{55} - v_{61}$$

$$\frac{d[UTPc]}{dt} = v_{60} - v_{55}$$

$$\frac{d[SUCPc]}{dt} = v_{56} - v_{57}$$

$$\frac{d[SUCc]}{dt} = v_{57} - v_{62}$$

$$\frac{d[PGAc]}{dt} = v_{32} - v_{pga\_use}$$

$$\frac{d(T3Pc)}{dt} = v_{31} + v_{33} - 2v_{51}$$

$$\frac{d(FBPc)}{dt} = v_{51} - v_{52}$$

$$\frac{d[HexPc]}{dt} = v_{52} - v_{55} - v_{59} + v_{58} - v_{56}$$

$$\frac{d[F26BPc]}{dt} = v_{59} - v_{58}$$

$$\frac{d[UDPGc]}{dt} = v_{55} - v_{56}$$

$$\frac{d[ATPc]}{dt} = v_{atpf} - v_{59} - v_{60}$$

Light reaction model

Description and Equations

The electron transport rate of the light reactions used in the model followed von Caemmerer (2000):

$$I_2 = \frac{1}{2} I \cdot \alpha \cdot (1 - f)$$

$$J = \frac{I_2 + J_{max} \pm \sqrt{(I_2 + J_{max})^2 - 4 I_2 J_{max}}}{2\theta}$$

$$V_{ATPsyn} = \min(\beta \cdot J, V_{E\_ATPsyn})$$

where: I_2 is the light absorbed by PS_{II}, J_{max} is the maximum electron transport rate, θ is an empirical curvature factor (0.7 was assumed following the value in a C_3 plant (Evans 1989)), β is

0.752 which is the convert constant of electron to ATP. α is leaf absorptance (0.85 after von Caemmerer (2000)), *f* corrects for spectral quality (0.15 according to Evans (1987)), V_{E_ATPsyn} is the ATP synthesis rate calculated from Michaelis-Menten equation of ATP synthase.

Parameters and values

Parameters and its respective values used in the model are described in the Table E.

Table E. Parameters of light reaction model

Parameters	Values	Reference
α	0.85	von Caemmerer (2000)
β	0.752	von Caemmerer (2000)
f	0.15	Evans (1987)
J_{max}	Same as in Table A	
θ	0.7	Evans (1989)

Rubisco activation model

Description

The detailed model describing the reactions involved in Rubisco activation and deactivation are described in Zhu et al. (2013).

Parameters and values

Parameters and its respective values used in the Rubisco activation model as well as the initial concentration of metabolites are described, respectively, in Tables F and G.

Table F. The rate constants of the rubisco activation model (Zhu et al., 2013)

Parameters	Values	Description
k1	1.5e-04	The rate constant of the activation of the Rubisco bound with RuBP
kn1	1.6e-03	The rate constant of E inactivation by binding of RuBP
km1	2 e-05	The Michaelis-Menton constant for RuBP with E
Ke2	0.1	Mate et al (1996)
Ke3	1.6	Mate et al (1996)
k6	3.75	Rubisco activity
kc	0.016	Michaelis-Menton constant for CO2
ko	0.448	Michaelis-Menton constant for O2
k7	k6 * 10	The rate constant for ECM to ECMR
kr	2 e-02	The apparent Michaelis-Menton constant for RuBP
RAC	0.006	Rubisco activase activity

Table G. The initial concentrations of metabolites

Parameters	Values	Description
ER	0.8	The concentration of inactive ER
Eaf	0.2	he total concentration of E, EC, AND ECM
ECMR	0.2	The concentration of ECMR
RuBP	2	The concentration RuB

<u>Equations from the model</u>

CA = 1

$$CB = K_{rae3} + \frac{K_{rae2} \times K_{rae3}}{[C]} + [Eaf] - MT$$

$$CC = - MT * (K_{rae3} + \frac{K_{rae2} * K_{rae3}}{[C]});$$

$$[Mg^{2+}] = \frac{-CB + \sqrt{CB^2 - 4CA \times CC}}{2CA}$$

$$EC = \frac{[Eaf]}{1 + \frac{K_{rae2}}{[C]} + \frac{[Mg^{2+}]}{K_{rae3}}}$$

$$E = \frac{EC \times K_{rae2}}{[C]}$$

$$ECM = \frac{EC * [Mg^{2+}]}{K_{rae3}}$$

$$LT = \frac{0.0216[Rubisco]}{[activase]}$$

$$RCA = \frac{1}{60 \times LT}$$

$$RDP = \frac{[ADP]}{[ATP]}$$

$$f_{ATP} = 1 - \frac{RDP}{3}$$

$$v_{ra1} = RCA \times [ER] \times f_{ATP}$$

$$v_{ran1} = k_{ran1} \times [E] \times [RuBP]$$

$$v_{ra7} = k_{ra7} \times [ECM] \times [RuBP]$$

$$v_{ran7} = k_{ran7} \times [ECMR]$$

$$k_{ran7} = k_{ra7} \times k_{r}$$

$$v_{ra6\_1} = \frac{k_{ra6} \times [ECMR] \times [C]}{([C] + k_c \times (1 + \frac{[O]}{k_o}))}$$

$$v_{ra6\_2} = \frac{k_{ra6} \times [ECMR] \times [O]}{([O] + k_o \times (1 + \frac{[C]}{k_c}))}$$

ET= ER+Eaf+ECMR

Percent = 1-ER/ET

$$\frac{d[ER]}{dt} = v_{ran1} - v_{ra1}$$

$$\frac{d[Eaf]}{dt} = v_{ra1} - v_{ra7} + v_{ran7} + v_{ra6\_1} + v_{ra6\_2} - v_{ran1}$$

$$\frac{d[ECMR]}{dt} = v_{ra7} - v_{ran7} - v_{ra6\_1} - v_{ra6\_2}$$

$$\frac{d[RuBP]}{dt} = v_{ra1} - v_{ra7} + v_{ran7} + v_{13} - v_{ran1}$$

Dynamic stomatal conductance response model

Description and Equations

Dynamic stomata conductance was calculated by the following ODE:

$$\frac{dg_s}{dt} = k(g_{steady} - g_s)$$

where: g_{steady} is calculated by the Ball-Berry model (Ball et al. 1987); k is k_i or k_d calculated from measured stomata dynamics in cassava.

Ball-Berry model parameters for predicting stomatal conductance (Ball et al. 1987) were obtained from light response curves measured in each one of the cultivars. The Ball-Berry model correlates stomatal conductance with net photosynthesis (A), relative humidity (RH_s) and CO_2 concentration at the leaf surface (Ca):

$$gs = mA\frac{RH_s}{Ca} + g_0$$

where: g_0 is the residual stomatal conductance when photosynthesis rate is zero, *m* is the slope of the relationship between g_s and *A*RHs/Ca*. g_0 and *m* were estimated by linear curve fitting.

Parameters and values

Parameters and values of *ki*, *kd*, Ball-Berry Slope and intercept were based on the values from Table A.

Cassava leaf model

Description and Equations

At the leaf level, the metabolic model and Rubisco activase model was integrated with leaf level models of stomatal physiology, and energy balance based on the method of (Nikolov et al. 1995).

Leaf boundary layer conductance

The leaf internal air space is assumed to be saturated and the saturation vapor pressure as a function of temperature:

$$\mathrm{East(Tleaf)} = 611\mathrm{exp}(\frac{17.502Tleaf}{Tleaf + 240.97})$$

$$Eair = RH\ Esat(Tair)$$

The boundary layer conductance is given as:

$$g_b = \max(g_{\mathrm{bforced}}, g_{\mathrm{bfree}})$$

$$g_{\mathrm{bforced}} = C_{forced}\mathrm{k}\mathrm{T}_{airk}^{0.56}\sqrt{(T_{airk} + 120)\frac{(v_w/l_d)}{1011325}}$$

$$g_{\mathrm{bfree}} = C_{free}\mathrm{T}_{airk}^{0.56}\sqrt{\frac{(T_{airk} + 120)}{1011325}\frac{(\Delta T)}{l_d}^{0.25}}$$

$$\Delta T = \frac{T_{leafk}}{1.0 - \frac{0.378E'_b}{101325}} - \frac{T_{airk}}{1.0 - \frac{0.378E_a}{101325}}$$

$$E_b = \frac{g_s E_i + g_{\mathrm{bforced}} E_a}{g_s + g_{\mathrm{bforced}}}$$

$$C_b = C_i - \frac{1.37 A_{net}}{g_b}$$

Leaf energy balance

The leaf energy balance equation under steady state equilibrium:

$$\mathrm{PAR} + \mathrm{NIR} + \mathrm{LW} = \mathrm{H} + \mathrm{LE} + \mathrm{E} + \mathrm{Me}$$

$$\mathrm{H} = 2C_p \cdot 0.924 g_b (T_{leaf} - T_{air})$$

$$LE = C_{LV} \frac{g_b g_s}{g_b + g_s} \frac{E_{sat} - E_{air}}{101325}$$

$$E = 2\varepsilon\sigma(T_{leafk})^4$$

$$\varepsilon = 1.72(\frac{E_{air}}{T_{airk}})^{1/7}$$

$$M_e = 0.506 A_{net}$$

Parameters and values

Parameters and its respective values used in this model are described in Table H.

Table H. Environmental parameters, values and units used in the cassava leaf model

Environmental condition	Value	Unit
Relative humidity	0.6	
Leaf dimension	0.06	m
Wind speed (v_w)	5	m s^{-1}
Temperature	25	^{o}C
Atmospheric pressure	101325	Pa
C_p	29.3	J mol−1 ◦C−1
C_{LV}	44000.0	J $mole^{-1}$
σ	5.6697E-8	W $m^{-2}K^{-4}$

REFERENCES

Ball J, Woodrow I, Berry J.1987. A model predicting stomatal conductance and its contribution to the control of photosynthesis under different environmental conditions. In: Biggins J (ed) *Progress in photosynthesis research.* Springer, Dordrech, pp 221-224

Evans J. 1989. Photosynthesis and nitrogen relationship in leaves of C_3 plants. *Oecologia* 7**8**: 9-19

Evans JR. 1987. The dependence of quantum yield on wavelength and growth irradiance. *Functional Plant Biology* **14**: 69-79

Mate C, von Caemmerer S, Evans JR, Hudson G, Andrews T. 1996. The relationship between CO_2-assimilation rate, Rubisco carbamylation and Rubisco activase content in activase-deficient transgenic tobacco suggests a simple model of activase action. *Planta* **198**: 604-613

Nikolov NT, Massman WJ, Schoettle AW. 1995. Coupling biochemical and biophysical processes at the leaf level: an equilibrium photosynthesis model for leaves of C_3 plants. *Ecological Modelling* **80**: 205-235

Vialet-Chabrand S, Matthews J, McAusland L, Blatt M, Griffiths H, Lawson T. 2017. Temporal dynamics of stomatal behavior" modelling and inplications for photosynthesis and water use. *Plant Physiology* **174**: 603-613

von Caemmerer S. 2000. *Biochemical models of leaf photosynthesis*. CSIRO Publishing, Collingwood, Australia

Zhu X-G, de Sturler E, Long SP. 2007. Optimizing the distribution of resources between enzymes of carbon metabolism can dramatically increase photosynthetic rate: A numerical simulation using an evolutionary algorithm. *Plant Physiology* **145**: 513-526

Zhu XG, Wang Y, Ort D, Long SP. 2013. e-photosynthesis: a comprehensive dynamic mechanistic model of C_3 photosynthesis: from light capture to sucrose synthesis. *Plant, Cell & Environment* **36**: 1711-1727
